# The Body Weight Alteration and Incidence of Neoplasm in Patients With Type 2 Diabetes: A Meta-Analysis of Randomized Controlled Trials

**DOI:** 10.3389/fendo.2020.541699

**Published:** 2020-12-23

**Authors:** Chu Lin, Xiaoling Cai, Wenjia Yang, Fang Lv, Lin Nie, Linong Ji

**Affiliations:** ^1^Department of Endocrinology and Metabolism, Peking University People’s Hospital, Beijing, China; ^2^Department of Endocrinology and Metabolism, Beijing Airport Hospital, Beijing, China

**Keywords:** body-weight trajectory, neoplasms, type 2 diabetes, hypoglycemic agents, cancer

## Abstract

**Objective:**

Whether hypoglycemic treatments with weight-alternating effects influence the incidence of neoplasm in type 2 diasbetes (T2D) remains uncertain. Therefore, we performed a meta-analysis to assess the association between the weight alteration and incidence of neoplasm in patients with T2D.

**Research Design and Methods:**

Systematic searches were conducted for studies published between the inception of 1950s and September 2019. Randomized controlled trials conducted in T2D patients with at least 48-week follow-up, significant weight change difference between treatment arms and reports of neoplasm events were included. Fixed-effects model and meta-regression analysis were accordingly used.

**Results:**

In all, 46 studies were included. Analysis indicated weight reduction was not associated with a decreased incidence of neoplasm (OR = 1.01, 95% CI, 0.96 to 1.07, *I*^2^ = 17%) and weight elevation was not associated with an increased incidence of neoplasm (OR = 0.91, 95% CI, 0.76 to 1.09, *I*^2^ = 0%). Meta-regression analysis showed a slower weight reduction rate (β = −5.983, 95% CI, −11.412 to 0.553, *P* = 0.03) instead of weight change difference (β = −0.030, 95% CI, −0.068 to 0.007, *P* = 0.115) was significantly associated with reduced risk of neoplasm in patients with T2D. Moreover, a decreased incidence of prostate, bladder, and uterine neoplasm was observed in T2D patients with weight reduction difference while an increased incidence of thyroid neoplasm was found in glucagon-like peptide-1 receptor analog (GLP-1RA) users with weight reduction difference.

**Conclusions:**

Additional weight change achieved by current hypoglycemic agents or strategies in short and medium periods was not associated with incidence of most neoplasm in patients with T2D. However, a decreased incidence of prostate, bladder, and uterine neoplasm was shown in T2D patients with weight reduction difference while an increased risk of thyroid neoplasm was observed in T2D patients on GLP-1RA treatments with weight reduction difference. A more sustained and persistent weight reduction process may confer reduced risk of neoplasm in patients with T2D.

## Highlights

1. What is already known about this subject?
  Epidemiological evidence suggested that obesity might be responsible for greater risk of neoplasm in patients with type 2 diabetes (T2D). However, evidence about interventional weight control effects on incidence of neoplasm in T2D is limited.
2. What are the new findings?
  By using data of long-term randomized controlled trials, we found that additional weight change achieved by current hypoglycemic agents or strategies in short and medium periods was not associated with incidence of most neoplasm in patients with T2D. However, A more sustained and persistent weight reduction process may confer reduced risk of neoplasm in patients with T2D.
3. How might these results change the focus of research or clinical practice?
  A more sustained and persistent weight reduction process may be more beneficial for patients with T2D in terms of neoplasm prevention. Studies with longer periods are encouraged to further validate the potential clinical benefit.

## Introduction

Type 2 diabetes (T2D) and cancer are two common non-communicable diseases with increasing prevalence all over the world (1). The linkage between T2D and cancer has become a heated interdisciplinary agenda involving both Endocrinology and Oncology. The increased incidence of cancer in patients with T2D has been reported in many epidemiological studies (2–4). A pooled analysis of cohort study enrolling 771,000 individuals in Asia also indicated baseline diabetes status was significantly associated with an increased risk of death from any cancer. Increased mortality was later confirmed in colorectum, liver, bile duct, gallbladder, pancreas, breast, endometrium, ovary, prostate, kidney, and thyroid cancer as well as lymphoma (5). In addition to hyperglycemia, obesity is recognized as one of the leading risk factors for multiple types of cancers in patients with T2D. Overweight and obesity are quite common comorbidities for T2D. There is sufficient level evidence supporting the association between overweight and 11 types of cancer, including colorectum and postmenopausal breast cancer (6). Hypothesis was proposed that the increased incidence of cancer and cancer mortality observed in T2D patients were possibly mediated by overweight and obesity. Underlying mechanisms include but not limit to insulin resistance, persistent inflammation, and oxidative DNA damage due to the excess adipose tissue (7). The interrupted endogenous environment may facilitate the occurrence and development of neoplasm, especially cancer.

However, an umbrella review of meta-analyses of observational studies evaluated the validity of the evidence between T2D and cancer as well as cancer mortality. It was found that only a minority of these associations (extrahepatic and gallbladder cancer) had robust supporting evidence without hints of bias (8). On the other hand, the evidence from the intervention studies associated with body weight change and the incidence of neoplasms in patients with T2D was quite limited. So far, the association between body weight and incidence of neoplasm in T2D patients remains uncertain.

In the blooming era of new hypoglycemic agents, such as sodium glucose cotransporter 2 inhibitor (SGLT2i) and glucagon-like peptide-1 receptor analog (GLP-1RA), glucose-lowering treatments in randomized controlled trials (RCTs) usually accompanied with weight-loss effects, which allowed us to evaluate the relationship between weight reduction and the incidence of neoplasms by intervention studies. Moreover, data from RCTs with traditional therapies such as insulin, thiazolidinedione (TZD), and sulfonylurea (SU) associated with body weight gain made it possible to evaluate the relationship between weight elevation and the incidence of neoplasms by intervention studies. Therefore, this meta-analysis of RCTs was designed to clarify the association between the alteration of body weight achieved by current hypoglycemic agents or strategies and incidence of neoplasm in patients with T2D.

## Methods

### Data Sources and Searches

According to recommendations from the Cochrane Handbook for Systematic Reviews for meta-analysis, two independent investigators (CL and NL) conducted systematic searches of Medline, Embase, and the Cochrane Central Register of Controlled Trials for studies published between the inception of 1950s (when metformin was studied in clinical trials and come into the market) and September 2019. The search terms were as follows: T2D; cardiovascular outcome trials (CVOTs); renal outcome trials (ROTs); intensive treatment; standard treatment; efficacy; placebo controlled; RCTs. The search strategies were as follows: 1) T2D and CVOTs or ROTs and RCTs; 2) T2D and intensive treatment and standard treatment and RCTs; 3) T2D and efficacy and RCTs; 4) T2D and placebo controlled and RCTs.

### Study Selection

The inclusion criteria for this meta-analysis were as follows: 1) studies with the reports of neoplasm events; 2) follow-up duration more than 48 weeks; 3) studies with significant weight change difference or weight change difference over 3 kg between treatments arms; 4) RCTs that fulfilled with at least one of the followings: a. cardiovascular studies with intensive treatment *versus* standard treatment in each arm, or b. efficacy evaluation studies with intensive active agent *versus* placebo or active control treatment in each arm, or c. CVOTs or ROTs with active agent *versus* placebo or active control treatment in each arm. The exclusion criteria for this meta-analysis were as follows: 1) studies conducted in type 1 diabetes patients or pre-diabetes patients; 2) follow-up duration less than 48 weeks; 3) studies without significant weight change difference between treatment arms or reports of neoplasm events.

### Data Extraction and Quality Assessment

One investigator (CL) abstracted data of all studies, including the publication data, study design, baseline characteristics, treatment arms, study duration, body weight change, and incidence of neoplasm, and assessed study quality. Another investigator (WY) checked abstractions and assessments for accuracy. The risk of bias was evaluated using the Cochrane risk of bias tool. Neoplasm events would be extracted from *clinicaltrial.gov* website with unique registered RCT number if the data was absent in both articles and supplementary materials. RCTs of the weight reduction group or weight elevation group was defined as that compared with the control group, weight change in the experimental group was decreased or increased respectively. Any disagreement was resolved by discussions with a third independent researcher (FL).

### Data Synthesis and Analysis

Continuous outcomes were evaluated by computing the weighted mean differences (WMDs) and 95% confidence intervals (CIs). Categorical outcomes were evaluated by computing the relative risks (RRs) or odds ratios (ORs) and accompanying 95% CIs. Higgins *I*^2^ statistics were used to evaluate the between-study heterogeneity, with an *I*^2^ value >50% indicating high level of heterogeneity. Fixed-effects model was used for low level of heterogeneity and random-effects model was used for high level of heterogeneity.

As for follow-up duration, we defined those <3 years as short period, those between 3 and 5 years as medium period, those >5 years as long period. It is different from the definition of “short-term” and “long-term” used in diabetes RCT design since we consider over 48 weeks as “long-term” for diabetes RCTs.

Furthermore, we defined a new variable as weight change difference rate, that is, weight change difference divided by follow-up duration in week unit. The variable represented the mean weight change difference in follow-up duration and indirectly reflected the extent of persistence for weight-changing status.

Meta-regression analysis was performed to evaluate whether the weight change differences and weight change difference rate were associated with the incidence of neoplasm. Publication bias was assessed *via* a funnel plot test. Statistical significance was considered at *P* < 0.05.

Statistical analyses were primarily performed by using the STATA statistical software package (version 11.0, Stata Corp, College Station, Texas, USA) and the Review Manager statistical software package (version 5.3, Nordic Cochrane Centre, Copenhagen, Denmark). Analyses were conducted according to the Preferred Reporting Items for Systematic Reviews and Meta-Analyses (PRISMA) guidelines for conducting and reporting meta-analyses of RCTs.

## Results

### Characteristics of Included Studies

**Figure 1:**
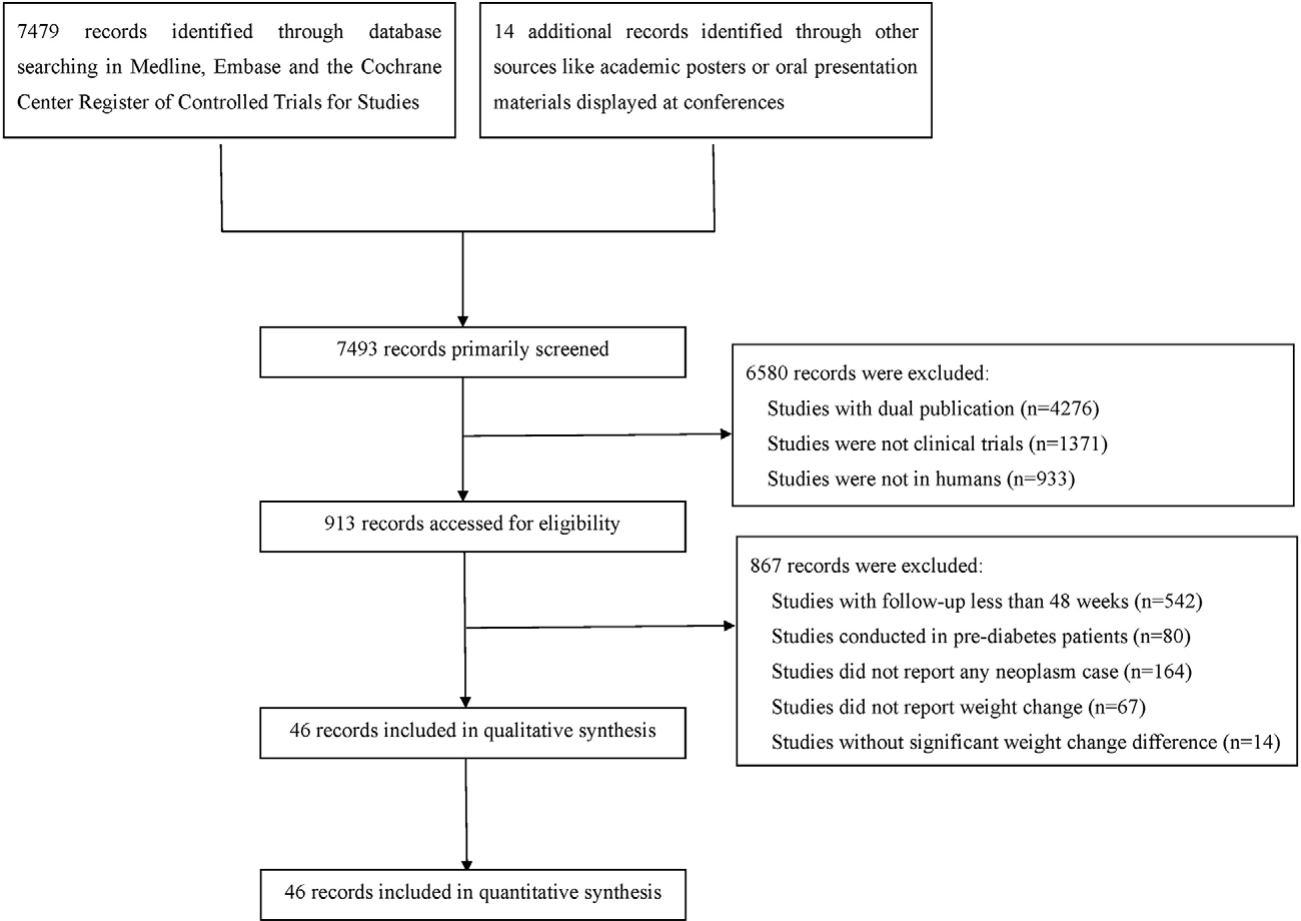
Flow diagram of the included studies.

After comprehensive literature search and selection, 46 eligible RCTs with 69061 participants in the experimental group and 59431 participants in the control group were included in this meta-analysis (**Figure 1**). There were 43 RCTs with weight reduction difference and 3 RCTs with weight elevation difference. In weight reduction group, there were 32 efficacy evaluation trials and 11 CVOTs or ROTs while all trials in weight evaluation group were comparisons between intensive and standard glucose-lowering treatments. The baseline characteristics of included RCTs were summarized in **Table S1**. Most individuals in these included studies were middle-aged subjects with overweight status or obesity. Among RCTs with available baseline BMI data, only three RCTs were with baseline BMI below 30 kg/m^2^ (two in weight reduction group and one in weight elevation group). The rest RCTs were all studies with baseline BMI over 30 kg/m^2^. All three RCTs with weight elevation difference were studies with long periods over 5 years. For RCTs with weight reduction difference, only two RCTs were with long period follow up, seven RCTs with medium periods while the rest 34 belonged to short period classification.

The risk of bias was evaluated by the Cochrane instrument, which suggested the overall risk of bias and the selective reporting was low (**Table S2**). The publication bias was accessed by funnel plot test (**Figure S1**).

### Weight Reduction and Incidence of Neoplasm

**Figure 2:**
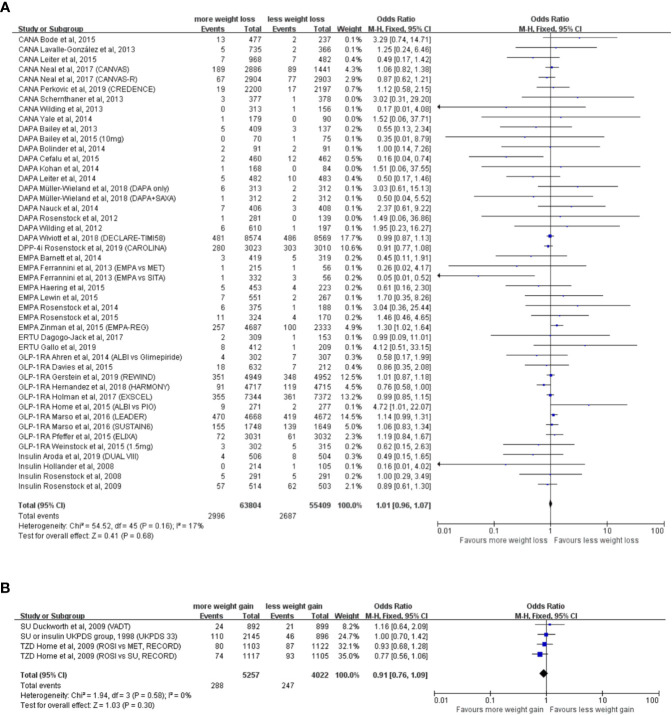
Weight alteration and incidence of neoplasm in randomized clinical trials of patients with type 2 diabetes. **(A)** Randomized controlled trials with weight reduction difference. CANA, canagliflozin; DAPA, dapagliflozin; EMPA, empagliflozin; ERTU, ertugliflozin; SAXA, saxagliptin; SITA, sitagliptin; GLP-1RA, glucagon-like peptide-1 receptor agonist; ALBI, albiglutide; DPP-4i, dipeptidyl-peptidase-4 inhibitors; MET, metformin; PIO, pioglitazone; PBO, placebo; CANVAS, Canagliflozin and Cardiovascular and Renal Events in Type 2 Diabetes; CANVAS-R, CANVAS-Renal; CREDENCE, Canagliflozin and Renal Outcomes in Type 2 Diabetes and Nephropathy; DECLARE-TIMI 58, The Dapagliflozin Effect on Cardiovascular Events-Thrombolysis in Myocardial Infarction; CAROLINA, Cardiovascular Outcome Study of Linagliptin *versus* Glimepiride in Patients with Type 2 Diabetes; EMPA-REG, Empagliflozin, Cardiovascular Outcomes, and Mortality in Type 2 Diabetes; HARMONY, Albiglutide and Cardiovascular Outcomes in Patients with Type 2 Diabetes and Cardiovascular Disease; REWIND, Dulaglutide and Cardiovascular Outcomes in Type 2 Diabetes; LEADER: Liraglutide and Cardiovascular Outcomes in Type 2 Diabetes; SUSTAIN6, Semaglutide and Cardiovascular Outcomes in Patients with Type 2 Diabetes; ELIXA, Lixisenatide in Patients with Type 2 Diabetes and Acute Coronary Syndrome; DUAL VIII, Durability of insulin degludec plus liraglutide versus insulin glargine U100 as initial injectable therapy in type 2 diabetes. **(B)** Randomized controlled trials with weight elevation difference. SU, sulfonylurea; TZD, thiazolidinedione; VADT, Veterans Affairs Diabetes Trial; ACCORD, The Action to Control Cardiovascular Risk in Diabetes; UKPDS, The UK Prospective Diabetes Study; RECORD, Rosiglitazone evaluated for cardiovascular outcomes in oral agent combination therapy for type 2 diabetes.

The weight reduction group consisted of 43 RCTs, with 63,804 participants in the experimental group and 55,409 participants in the control group. Analysis indicated that weight reduction was not associated with reduced incidence of neoplasm (OR = 1.01, 95% CI, 0.96 to 1.07, *I*^2^ = 17%) (**Figure 2A****)**, although statistically significant weight reduction difference was achieved (WMD = −2.43kg, 95% CI, −2.62 to −2.24 kg, *P* < 0.001).

Subgroup analysis according to the types of hypoglycemic agents including GLP-1RA, DPP-4i, SGLT2i, and insulin showed that none of these hypoglycemic agents were associated with decreased incidence of neoplasm in weight reduction group (**Figure S2**). Subgroup analysis based on different study types, including efficacy evaluation trials and CVOTs or ROTs, suggested no significant relationships between the weight reduction and incidence of neoplasm either (**Figure S3****)**.

We labeled those RCTs with reports of neoplasm events in original articles or supplementary materials and performed the subgroup analysis. Nevertheless, the conclusion still turned out to be negative regardless of the source of neoplasm events (**Figure S4**).

Baseline body mass index (BMI) is a critical variable which represents the magnitude of the excess of weight at the beginning of the enrollment. However, the sensitivity analysis stratified by baseline BMI indicated that the degree of baseline BMI did not influence the association between weight reduction difference and incidence of neoplasm in T2D patients (**Figure S5**).

No significant relationships were observed in further subgroup analyses stratified by treatment design (exclusive or add-on design), hypoglycemic agents used in control groups, patient age, male percentage, follow-up duration and diabetes duration **(****Figures S6**–**11****)**.

### Weight Elevation and Incidence of Neoplasm

The weight elevation group comprised three RCTs, with 5,257 participants in the experimental group and 4,022 participants in the control group. With a statistically significant weight elevation difference (WMD = 4.53 kg, 95%CI, 3.01 to 6.05 kg, *P *< 0.001) between active and control groups, weight elevation was not associated with increased incidence of neoplasm (OR = 0.98, 95% CI, 0.90 to 1.06, *I*^2^ = 0%) (**Figure 2B**).

### Overall Meta-Regression Analysis

**Figure 3:**
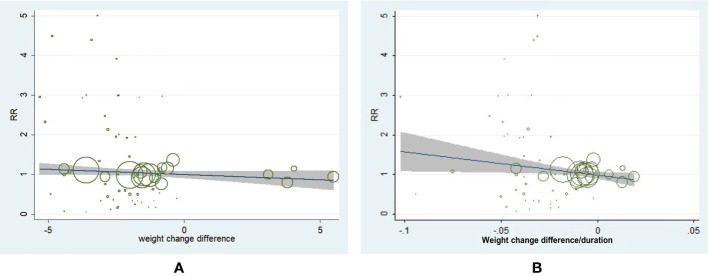
Meta-regression analysis of weight change difference and change rate with incidence of neoplasm in patients with type 2 diabetes. **(A)** Weight change difference and incidence of neoplasm (β = −0.030, 95% CI, −0.068 to 0.007, *P* = 0.115). **(B)** Weight change rate and incidence of neoplasm (β = −5.983, 95% CI, −11.412 to 0.553, *P* = 0.031).

We performed the meta-regression analysis and the result showed that weight change difference value was not associated with incidence of neoplasm in T2D (β = −0.030, 95% CI, −0.068 to 0.007, *P* = 0.115) (**Figure 3A**). However, a slower weight reduction rate was associated with a decreased incidence of neoplasm in T2D (β = −5.983, 95% CI, −11.412 to 0.553, *P* = 0.03) (**Figure 3B**). We further conducted the adjustments by patient age, male percentage, baseline BMI, and diabetes duration for meta-regression analysis of weight change difference as well as weight change difference rate with incidence of neoplasm. It turned out the corresponding outcomes remained consistent, which further indicated the adjusted demographic variables did not influence the main outcomes.

### Analysis by Neoplasm Sites

**Figure 4:**
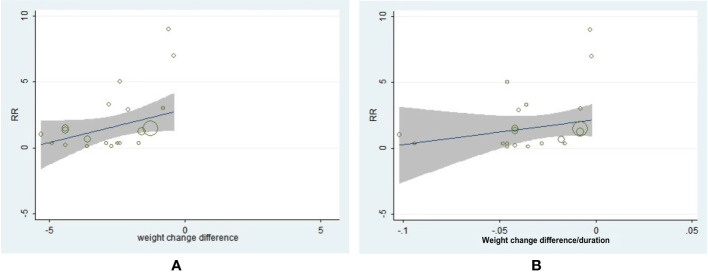
The association between weight reduction difference or change rate and the incidence of uterine neoplasm. **(A)** Weight reduction difference and incidence of uterine neoplasm (β = 0.8743, 95% CI, 0.2206 to 1.5280, *P* = 0.012). **(B)** Weight reduction rate and incidence of uterine neoplasm (β = 34.062, 95% CI, −6.3617 to 74.4857, *P* = 0.094).

The neoplasm-site data of 17 pre-specific neoplasm were also collected and analyzed (**Table S3**). Interestingly, a decreased incidence of prostate (OR = 0.77, 95% CI, 0.61 to 0.97, *I*^2^ = 0%) and bladder neoplasm (OR = 0.72, 95%CI, 0.56 to 0.95, *I*^2^ = 0%) were exhibited in patients with T2D with significant weight reduction difference (**Table S3**). The following drug-type analysis suggested such reduced risk of prostate neoplasm may be driven by the utilization of GLP-1RA (OR = 0.68, 95% CI, 0.49 to 0.95, *I*^2^ = 0%). Interestingly, we also observed two-fold risk of thyroid neoplasm in GLP-1RA users with weight reduction difference (OR = 2.00, 95% CI, 1.10 to 3.63, *I*^2^ = 0%). Further meta-regression analysis by specific neoplasm sites indicated greater weight reduction difference was associated with lower incidence of uterine neoplasm (β = 0.8743, 95% CI, 0.2206 to 1.5280, *P* = 0.012) (**Figure 4A**), but no similar association was observed with weight reduction difference rate (**Figure 4B**). As for the meta-regression analyses for the rest neoplasm sites, no further significant associations were confirmed, regardless of assessments for weight change difference or weight change difference rate (**Figures S12**–**27**).

## Discussion

This is the first and the largest meta-analysis elucidating the association between the alteration of body weight and incidence of neoplasm in T2D. By using data from RCTs with longer study duration, we found that the additional weight alteration achieved by current glucose-lowering agents or strategies was not associated with decreased or increased incidence of most obesity-related or diabetes-related neoplasm in T2D patients. However, a slower weight reduction rate may confer reduced risk of neoplasm in patients with T2D in general.

So far, most of studies focusing on obesity and neoplasm or cancer were cross-sectional or cohort studies (9–11). Moreover, prospective studies evaluating the association between weight control and its effects on neoplasm were lacking or non-specific. Previous meta-analyses mostly assessed the effects of weight loss interventions for adults with obesity instead of T2D (12). A systematic review focusing on bariatric surgery in patients with obesity causing body weight reduction did show a significant reduction in the risk of cancer with randomized evidence (13). Another meta-analysis showed very low quality of evidence for an effect of weight loss on cancer mortality and new-onset cancer (14). However, individuals in these reviews were non-diabetes patients or population comprising more than patients with T2D, or studies enrolled were all cohort studies, or the number of trials was small. Plus, rare studies evaluated the weight change rate with the risk of cancer in patients with T2D. Therefore, based on the past evidence, it was not clear whether the weight reduction was associated with decreased incidence of neoplasm especially in patients with T2D.

In our meta-analysis, RCTs in T2D patients with significant weight change difference (or over 3 kg) between active and control arms and over 48-week follow up were included. Such eligible studies were RCTs with the primary aim to evaluate efficacy and safety or to assess cardiovascular and renal benefit for hypoglycemic agents. Unlike the agents specifically designed for weight loss, the proportion of a durable weight loss of at least 5 kg or 5% of the basal body weight achieved by hypoglycemic agents was pretty low. In fact, we screened out all RCTs in T2D with report of weight change, and the range for weight change difference absolute value was from 0 to 5.3kg. Only about 20% of RCTs could achieve over 3 kg weight change difference. It suggests the weight-alteration capacity of hypoglycemic agents is different from weight-loss agents such as orlistat. There may be a “ceiling effect” for weight alteration achieved by hypoglycemic agents in current follow-up duration. Therefore, we adjusted the enrollment criteria as RCTs with significant weight change difference or weight change difference over 3 kg for this analysis.

As we know, the period since one or more risk factors appear until a specific type of neoplasm is diagnosed is variable but usually very long. Therefore, we excluded the studies with follow-up duration below 48 weeks since they may dilute the outcomes. However, among RCTs conducted in T2D patients, few of them could last for over 5 years. Therefore, we enrolled relatively “long-term” RCTs (over 48 weeks) in term of study design and differentiated short, medium, and long period duration for sensitivity analyses. It would be a great pity if the potential clinical value of such RCTs in T2D was ignored and left unassessed. All these factors taken into consideration, our meta-analysis still showed that although additional weight change difference achieved by current hypoglycemic agents or strategies was not associated with incidence of most neoplasm in T2D patients, prostate, bladder, and uterine neoplasm were less frequent in T2D patients with significant weight reduction difference. Furthermore, a slower weight reduction rate was associated reduced incidence of neoplasm, which means a more sustained and persistent weight reduction process might be more beneficial for neoplasm prevention.

There are some confounders that might be associated with the incidence of neoplasm. Since participants in these RCTs received hypoglycemic treatments, the body weight change of these patients was accompanied by improved glucose control, or recognized as reduced HbA1c in these RCTs. However, a previous meta-analysis using data from large RCTs of intensified glycemic control suggested that cancer risk was not reduced by improving glycemic control in patients with T2D (15). So far, there is no solid evidence proofing that hyperglycemia is causally linked to increased cancer risk. The patient age is another influencing factor to consider. Generally, elderly patients are exposed to greater risk of neoplasm. However, a controlled cohort study found that obesity-related cancer could be attributed to overweight at any age (16). In our subgroup analysis, we did not notice any difference between elderly patients (≥60 years old) and middle-age or younger patients (<60 years old) either. Sex differences in the risk of developing obesity-related cancer were also noted in previous studies (16). Greater risk of colorectal cancer has been reported in men than in women possibly due to the lower circulating level of adiponectin in men (17). However, this difference did not sustain in the subgroup comparison in our study, which might be associated with the blurry division based on the male percentage in this meta-analysis. More accurate division is needed to confirm this phenomenon.

Higher BMI and longer duration of overweight are more susceptible obesity-related cancers. Arnord et al. validated an increased incidence of obesity-related cancer in patients with longer duration of overweight by multivariate cox models and random effects analyses (18). They further pointed out that the degree of overweight experienced during adulthood might play an important role in the risk of developing cancer (18). Correspondingly, we conducted sensitivity analyses according to the degree of BMI and duration of T2D instead of obesity but no significant effects on the incidence of neoplasm were observed in patients with higher baseline BMI (>30 kg/m^2^). Likewise, T2D patients with longer diabetes duration did not show significant difference in incidence of neoplasm either. One important thing to note, since the majority of baseline BMI in included RCTs were over 30 kg/m^2^, we did not have much information about individuals with normal range or lower BMI. The conclusion from this meta-analysis may not apply to that specific population.

For different sites of neoplasm, previous epidemiological studies also unveiled certain linkages. In April 2016, the International Agency for Research on Cancer (IARC) reassessed the preventive effects of weight control on cancer risk and released an official report on cancer-preventive effect of the absence of excess body fatness (19). The following cancer sites or types were with sufficient strength of evidence in humans: esophagus adenocarcinoma, gastric cardia, colon and rectum, liver, gallbladder, pancreas, breast (postmenopausal), corpus uteri, ovary, kidney (renal-cell), meningioma, thyroid and multiple myeloma (19). Besides, higher BMI was reported with increased risk of prostate and bladder cancer in multiple cohort studies (20–22). Hence, 17 types of neoplasm of interest were accordingly chosen for subgroup analyses. Using meta-regression method, we found that more weight reduction would significantly lower the risk of uterine neoplasm in patients with T2D. Actually, previous studies have repeatedly confirmed elevation in BMI would increase the risk of developing endometrial cancer in women and morbid obesity was associated with higher cancer mortality (23). Weight loss intervention may reduce risk of endometrial cancer by resolving the chronic inflammation and redressing the hormone imbalance since adipose tissue could process estrogen conversion and increase circulating estrogen level (24). Our result further supported the weight reduction for uterine neoplasm prevention, especially for endometrial cancer.

Moreover, the incidence of prostate and bladder neoplasm was also decreased in patients with reduction of weight change. However, the subgroup results shown in **Table S3** were not that consistent with meta-regression analysis. It is possible that neoplasm events were not be fully evaluated, which may influence the results. Moreover, some subgroup results may be influenced by agents with weight reducing effect, such as GLP-RA in prostate neoplasm. The inconsistent results indicated the reduced neoplasm risk may not be mediated by weight reduction but drug innate properties instead. Therefore, the results of pre-specific neoplasm sites should be interpreted with caution.

In sub-analysis upon different drug-types, we found that GLP-1RA was associated with decreased incidence of prostate neoplasm in weight reduction group. With generally only 5% expression of GLP-1R on prostate adenocarcinoma (25), GLP-1RA was likely to repress the development of prostate neoplasm beyond GLP-1R. Cell-level experiments suggested exendin (Ex)-4, a GLP-1RA, attenuated prostate cancer growth through the inhibition of extracellular signal–regulated kinase (ERK)-mitogen-activated protein kinase (MAPK) activation (26), which may be responsible for this result. Interestingly, we observed an increased risk of thyroid neoplasm in patients with T2D on GLP-1RA treatments. Although animal experiments showed GLP-1RA could induce thyroid C-cell carcinogenicity in rodents, no relevant events were ever reported in human yet (27). More studies are needed to enrich the drug safety profile.

In terms of SGLT2i treatment, Tang et al. assessed the risk of cancer and utilization of SGLT2i and indicated non-significantly increased incidence of neoplasm in patients with T2D (28), which was consistent with our findings. For insulin users, a five-country cohort study found a few increased or decreased risks of cancers in men and women but no evidence of consistent differences was shown between analogues and human insulin (29). In our study, we did not observe any significant association with neoplasm in insulin users. A Cochrane meta-analysis of RCTs did not find any significant beneficial effects of SU on cancer (30), which was consistent with our meta-analysis. As for TZD, a recent meta-analysis of case-control and cohort studies found that TZD were associated with a reduced risk of colorectal and liver cancer and an increased risk of bladder cancer (31). However, neoplasm-site data were not available in the included RCT with TZD in our study, we were unable make any conclusion.

### Strengths and Limitations

Our study has several strengths. It is a very comprehensive meta-analyses of almost 50 RCTs with more than 60,000 participants. Existing high-level evidence has been synthesized to unprecedentedly demonstrate the association between weight change by hypoglycemic agents and incidence of neoplasm in patients with T2D. It was proposed that a durable weight reduction process may receive greater clinical benefit for neoplasm prevention.

There are also some limitations in our study. First, the RCTs enrolled in this meta-analysis were all anti-diabetic medication trials. Actually, we did screen out RCTs with simply lifestyle intervention known as diet and exercise. However, these lifestyle intervention oriented RCTs did not report neoplasm events, such as Cardiovascular Effects of Intensive Lifestyle Intervention in Type 2 Diabetes (lookAHEAD) (32) and Treatment Options for Type 2 Diabetes in Adolescents and Youth (TODAY) (33), which were excluded in eligibility assessment phase. Besides, some large-scale RCTs such as Effect of Sitagliptin on Cardiovascular Outcomes in Type 2 Diabetes (TECOS) (34), The Cardiovascular and Renal Microvascular Outcome Study with Linagliptin (CARMELINA) (35), and Basal Insulin and Cardiovascular and Other Outcomes in Dysglycemia (ORIGIN) (36) were also excluded due to no reports of body weight change or without significant weight change difference between two arms. We also screen for agents which can reduce body weight without obvious glycemic control, including orlistat (37–41), Lorcaserin (42), Naltrexone Sustained-Release/Bupropion Sustained-Release (43). However, no RCTs with these agents in T2D patients reported neoplasm events. Thus, they were also excluded.

Second, none of these RCTs were taking incidence of neoplasm as their primary endpoints. Hence, the reported data about neoplasm events was probably incomplete. Specific-site neoplasm data was missing in some RCTs.

We did not distinguish malignant and benign neoplasm in further analysis since the benign neoplasm was rarely reported within these RCTs. A convincing conclusion was unable to be made based on such limited data.

We did not have access to individual-level data, but it did not influence the primary conclusion of this meta-analysis. In order to avoid the dilution effect of short-term RCTs, we only selected RCTs with follow-up visit over 48 weeks. Still, more large-scale RCTs with longer follow-up periods should be included to make a definite conclusion.

We defined weight change difference rate under the hypothesis that weight changed in a uniform speed. However, body weight may not always change in a linear status along the time. This variable only represents a mean weight change rate during the studies. Future studies need to record weight changes in a dynamic timeline and depict them with accurate models.

### Recommendations for Future Research

For future study design, we should evaluate overweight and obesity in multiple dimensions and introduce more indicators in RCTs such as waist-to-hip ratio. Our study has already showed a more sustained weight reduction may confer a reduced risk of neoplasm in T2D patients. With longer follow-up duration, we may portray the track of weight change to assess the association between peak weight change difference and incidence of neoplasm, and possibly, explore the potential weight change threshold for neoplasm prevention in patients with T2D.

## Conclusions

According to our meta-analysis, additional weight change achieved by current hypoglycemic agents or strategies in short and medium periods was not associated with incidence of most neoplasm in patients with T2D. However, a decreased incidence of prostate, bladder, and uterine neoplasm was shown in T2D patients with weight reduction difference while an increased risk of thyroid neoplasm was observed in T2D patients on GLP-1RA treatments with weight reduction difference. A more sustained and persistent weight reduction process may confer reduced risk of neoplasm in patients with T2D.

## Data Availability Statement

All data relevant to the study are included in the article or uploaded as **Supplementary Material**. No more additional data are available.

## Author Contributions

LJ and XC conceptualized this study and designed the systematic review protocol. CL, WY, FL, and LN performed the study selection and data extraction. CL and XC performed the statistical analyses. CL and XC prepared the outlines and wrote the manuscript. All authors contributed to the article and approved the submitted version.

## Funding

This work was supported by National Key Research and Development Program of China (No.2016YFC1304901) and National Natural Science Foundation of China (81970698). The funding agencies had no roles in the study design, data collection or analysis, decision to publish, or preparation of the manuscript.

## Conflict of Interest

LJ has received fees for lecture presentations and for consulting from AstraZeneca, Merck, Metabasis, MSD, Novartis, Eli Lilly, Roche, Sanofi-Aventis, and Takeda.

The remaining authors declare that the research was conducted in the absence of any commercial or financial relationships that could be construed as a potential conflict of interest.

## Abbreviations

T2D, type 2 diabetes; SGLT2i, sodium glucose cotransporter 2 inhibitor; GLP-1RA, glucagon-like peptide-1 receptor analog; RCT, randomized controlled trial; TZD, thiazolidinedione; SU, sulfonylurea; CVOT, cardiovascular outcome trials; ROT, renal outcome trials; WMD, weighted mean difference; CI, confidence interval; RR, relative risk; OR, odds ratio; PRISMA, Preferred Reporting Items for Systematic Reviews and Meta-Analyses; HARMONY, Albiglutide and Cardiovascular Outcomes in Patients with Type 2 Diabetes and Cardiovascular Disease; CAROLINA, Cardiovascular Outcome Study of Linagliptin *versus* Glimepiride in Patients with Type 2 Diabetes; BMI, body mass index; IARC, the International Agency for Research on Cancer; ERK, extracellular signal–regulated kinase; MAPK, mitogen-activated protein kinase; lookADEAD, Cardiovascular Effects of Intensive Lifestyle Intervention in Type 2 Diabetes; TODAY, Treatment Options for Type 2 Diabetes in Adolescents and Youth; TECOS, Effect of Sitagliptin on Cardiovascular Outcomes in Type 2 Diabetes; CARMELINA, The Cardiovascular and Renal Microvascular Outcome Study with Linagliptin; ORIGIN, Basal Insulin and Cardiovascular and Other Outcomes in Dysglycemia.
