# Supplementary material for: The Body Weight Alteration and Incidence of Neoplasm in Patients With Type 2 Diabetes: A Meta-Analysis of Randomized Controlled Trials
Source: Front Endocrinol (Lausanne). 2020 Dec 23;11:541699. doi: 10.3389/fendo.2020.541699 (PMC7793753; doi:10.3389/fendo.2020.541699)
Supplement: Supplementary file 1 [file Table_1.docx]

**SUPPLEMENTARY MATERIALS**

**Supplement Table legends**

Table S1. Baseline characteristics of studies included in this meta-analysis

Table S2. Risk of bias for included studies in this meta-analysis

Table S3. Association between incidence of neoplasm and weight reduction difference stratified by neoplasm sites

**Supplement Figure legends**

Figure S1. Funnel plot figure of publication bias for randomized clinical trials

Figure S2. The association between weight reduction difference and the incidence of neoplasm stratified by drug types

Figure S3. The association between weight reduction difference and the incidence of neoplasm stratified by study types

Figure S4. The association between weight reduction difference and the incidence of neoplasm stratified by source of neoplasm events

Figure S5. The association between weight reduction difference and the incidence of neoplasm stratified by patient body mass index

Figure S6. The association between weight reduction difference and the incidence of neoplasm stratified by treatment design

Figure S7. The association between weight reduction difference and the incidence of neoplasm stratified by control agents

Figure S8. The association between weight reduction difference and the incidence of neoplasm stratified by patient age

Figure S9. The association between weight reduction difference and the incidence of neoplasm stratified by male percentage

Figure S10. The association between weight reduction difference and the incidence of neoplasm stratified by follow-up duration

Figure S11. The association between weight reduction difference and the incidence of neoplasm stratified by duration of diabetes

Figure S12. The association between weight reduction difference or change rate and the incidence of gastric neoplasm

Figure S13. The association between weight reduction difference or change rate and the incidence of colon/rectal neoplasm

Figure S14. The association between weight reduction difference or change rate and the incidence of lung neoplasm

Figure S15. The association between weight reduction difference or change rate and the incidence of pancreatic neoplasm

Figure S16. The association between weight reduction difference or change rate and the incidence of skin neoplasm

Figure S17. The association between weight reduction difference or change rate and the incidence of prostate neoplasm

Figure S18. The association between weight reduction difference or change rate and the incidence of breast neoplasm

Figure S19. The association between weight reduction difference or change rate and the incidence of bladder neoplasm

Figure S20. The association between weight reduction difference or change rate and the incidence of brain neoplasm

Figure S21. The association between weight reduction difference or change rate and the incidence of hematologic neoplasm

Figure S22. The association between weight reduction difference or change rate and the incidence of thyroid neoplasm

Figure S23. The association between weight reduction difference or change rate and the incidence of ovarian neoplasm

Figure S24. The association between weight reduction difference or change rate and the incidence of renal neoplasm

Figure S25. The association between weight reduction difference or change rate and the incidence of hepatic neoplasm

Figure S26. The association between weight reduction difference or change rate and the incidence of gallbladder/bile duct neoplasm

Figure S27. The association between weight reduction difference or change rate and the incidence of head and neck neoplasm

**Table S1. Baseline characteristics of studies included in this meta-analysis**

| **Author, year** | **Study duration** | **Treatment group** | **No. of patients** | **Age (years)** | **Men (%)** | **BMI (kg/m2)** | **Weight(kg)** | **Duration of diabetes**  **(years)** | **Baseline HbA1c (%)** |
| --- | --- | --- | --- | --- | --- | --- | --- | --- | --- |
| **Intensive vs standard treatment** | | | | | | | | | |
| Turner, 1998^1^  (UKPDS 33) | 10 years | Intensive | 2729 | 53.2±8.6 | 60.6 | 27.5±5.1 | 77.3±15.4 | / | 7.09±1.5 |
|  |  | Conventional | 1138 | 53.4±8.6 | 61.9 | 27.8±5.5 | 78.1±16.3 | / | 7.05±1.4 |
| Duckworth, 2009^2^  (VADT) | 78 months | Intensive | 892 | 60.5±9.0 | 97.1 | 31.3±3.0 | 96.3±16.2 | 11.5±8.0 | 9.4±2.0 |
|  |  | Standard | 899 | 60.3±9.0 | 97.1 | 31.3±4.0 | 96.3±16.2 | 11.5±7.0 | 9.4±2.0 |
| Home, 2009^3^  (RECORD) | 5.5 years | Rosiglitazone + MET | 1117 | 57.0±8.0 | 53.8 | 32.8±5.0 | 93.5±16.5 | 6.1±4.2 | 7.8±0.7 |
|  |  | SU + MET | 1105 | 57.2±8.1 | 52.9 | 32.7±5.2 | 93.3±16.3 | 6.3±4.4 | 7.8±0.7 |
|  |  | Rosiglitazone + SU | 1103 | 59.8±8.3 | 49.0 | 30.3±4.1 | 85.0±14.5 | 7.9±5.5 | 8.0±0.7 |
|  |  | MET + SU | 1122 | 59.7±8.2 | 50.6 | 30.1±4.3 | 84.3±14.4 | 7.9±5.2 | 8.0±0.7 |
| Active agent vs placebo treatment in efficacy evaluation | | | | | | | | | |
| Insulin | | | | | | | | | |
| Hollander, 2008^4^ | 52 weeks | Detemir | 214 | 59±11.0 | 60.7 | 31.5±4.7 | 93.3±17.5 | 13.6±8.1 | 8.6±1.0 |
|  |  | Glargine | 105 | 58±11.0 | 52.4 | 31.7±4.7 | 91.5±17.9 | 13.4±7.8 | 8.8±1.1 |
| Rosenstock, 2008^5^ | 52 weeks | Detemir | 291 | 58.4±10.2 | 57.0 | 30.6±4.8 | 87.4±16.6 | 9.1±6.1 | 8.64±0.8 |
|  |  | Glargine | 291 | 59.4±9.6 | 58.8 | 30.5±4.6 | 87.4±17.4 | 9.1±6.4 | 8.62±0.8 |
| Rosenstock, 2009^6^ | 5 years | Glargine | 513 | 54.9±8.8 | 54.2 | 34.5±7.2 | 100.2±22.7 | 10.7±6.9 | 8.41±1.4 |
|  |  | NPH | 504 | 55.3±8.5 | 53.6 | 34.1±7.2 | 98.7±22.3 | 10.8±6.7 | 8.31±1.4 |
| Aroda, 2019^7^ | 104 weeks | Degludec + liraglutide | 506 | 56.8±10.0 | 55.0 | 32.0±6.2 | 89.7±20.5 | 10.0±6.2 | 8.4±1.0 |
| (DUAL VIII) |  | Glargine | 506 | 56.4±10.1 | 54.0 | 31.9±5.8 | 89.0±20.1 | 10.2±6.1 | 8.6±1.0 |
| GLP-1RA |  |  |  |  |  |  |  |  |  |
| Ahren, 2014^8^ | 104 weeks | Albiglutide | 302 | 54.3±10.1 | 44.7 | 32.7±5.6 | 90.3±19.1 | 6.0±4.3 | 8.1±0.8 |
|  |  | Sitagliptin | 302 | 54.3±9.8 | 46 | 32.5±5.4 | 91.8±20.4 | 5.8±4.8 | 8.1±0.8 |
|  |  | glimepiride | 307 | 54.4±10 | 51.5 | 32.5±5.5 | 89.6±18.4 | 6.0±4.8 | 8.1±0.8 |
|  |  | PBO | 101 | 56.1±10 | 49.5 | 32.8±5.4 | 91.6±19.3 | 6.7±6.6 | 8.2±0.9 |
| Weinstock, 2015^9^ | 2 years | Dulaglutide 1.5mg | 304 | 54±10 | 48.0 | 31.0±5.0 | 86.0±17.0 | 7.0±6.0 | 8.1±1.1 |
|  |  | Dulaglutide 0.75mg | 302 | 54±10 | 44.0 | 31.0±4.0 | 87.0±17.0 | 7.0±5.0 | 8.2±1.1 |
|  |  | Sitagliptin | 315 | 54±10 | 48.0 | 31.0±4.0 | 86.0±18.0 | 7.0±5.0 | 8.1±1.1 |
|  |  | PBO | 177 | 55±9 | 51.0 | 31.0±4.0 | 87.0±17.0 | 7.0±5.0 | 8.1±1.1 |
| Davies, 2015^10^ | 56 weeks | Liraglutide 3.0mg | 423 | 55.0±10.8 | 52.0 | 37.1±6.5 | 105.7±21.9 | 7.5±5.65 | 7.9±0.8 |
|  |  | Liraglutide 1.8mg | 211 | 54.9±10.7 | 51.2 | 37.0±6.9 | 105.8±21.0 | 7.4±5.16 | 8.0±0.8 |
|  |  | PBO | 212 | 54.7±9.8 | 45.8 | 37.4±7.1 | 106.5±21.3 | 6.7±5.07 | 7.9±0.8 |
| Home, 2015^11^ | 156 weeks | Albiglutide + MET + SU | 271 | 54.5±9.5 | 49.8 | 32.4±5.5 | 90.9±20.2 | 8.5±6.3 | 8.19±0.9 |
|  |  | PIO + MET + SU | 277 | 55.7±9.4 | 53.4 | 32.5±5.7 | 91.0±21.2 | 9.2±6.1 | 8.29±0.9 |
|  |  | PBO + MET + SU | 115 | 55.7±9.6 | 60.9 | 31.8±4.9 | 89.9±18.8 | 9.3±6.1 | 8.26±1.0 |
| SGLT2 inhibitors |  |  |  |  |  |  |  |  |  |
| Wilding, 2012^12^ | 48 weeks | Dapagliflozin2.5mg + INS | 202 | 59.8±7.6 | 49.5 | 33.0±5.0 | 93.0±16.7 | 13.6±6.6 | 8.46±0.8 |
|  |  | Dapagliflozin 5mg + INS | 211 | 59.3±7.9 | 47.4 | 33.0±5.3 | 93.3±17.4 | 13.1±7.8 | 8.62±1.0 |
|  |  | Dapagliflozin 10mg + INS | 194 | 59.3±8.8 | 44.8 | 33.4±5.1 | 94.5±16.8 | 14.2±7.3 | 8.57±0.8 |
|  |  | PBO + INS | 193 | 58.8±8.6 | 49.2 | 33.1±5.9 | 94.5±19.8 | 13.5±7.3 | 8.47±0.8 |
| Rosenstock, 2012^13^ | 48 weeks | Dapagliflozin 5mg + PIO | 141 | 53.2±10.9 | 55.3 | / | 87.8±20.7 | 5.64±5.36 | 8.40±1.0 |
|  |  | Dapagliflozin 10mg + PIO | 140 | 53.8±10.4 | 42.1 | / | 84.8±22.2 | 5.75±6.44 | 8.37±1.0 |
|  |  | PBO + PIO | 139 | 53.5±11.4 | 51.1 | / | 86.4±21.3 | 5.07±5.05 | 8.34±1.0 |
| Schernthaner, 2013^14^ | 52 weeks | Canagliflozin 300mg | 377 | 56.6±9.6 | 54.9 | 31.5±6.9 | 87.4±23.2 | 9.4±6.1 | 8.1±0.9 |
|  |  | Sitagliptin 100 mg | 378 | 56.7±9.3 | 56.9 | 31.7±6.9 | 89.1±23.2 | 9.7±6.3 | 8.1±0.9 |
| Wilding, 2013^15^ | 52 weeks | Canagliflozin 100mg | 157 | 57.4±10.5 | 51.6 | 33.3±6.3 | 93.8±22.6 | 9.0±5.7 | 8.1±0.9 |
|  |  | Canagliflozin 300mg | 156 | 56.1±8.9 | 44.2 | 33.2±6.3 | 93.5±22.0 | 9.4±6.4 | 8.1±0.9 |
|  |  | PBO | 156 | 56.8±8.3 | 51.3 | 32.7±6.8 | 91.2±22.6 | 10.3±6.7 | 8.1±0.9 |
| Lavalle-González, 2013^16^ | 52 weeks | Canagliflozin 100mg | 368 | 55.5±9.4 | 47.3 | 31.4±6.3 | 88.8±22.2 | 6.7±5.4 | 7.9±0.9 |
|  |  | Canagliflozin 300mg | 367 | 55.3±9.2 | 45.0 | 32.4±6.4 | 85.4±20.9 | 7.1±5.4 | 7.9±0.9 |
|  |  | Sitagliptin | 366 | 55.5±9.6 | 47.0 | 32.0±6.1 | 87.7±21.6 | 6.8±5.2 | 7.9±0.9 |
|  |  | PBO/ Sitagliptin | 183 | 55.3±9.8 | 51.4 | 31.1±6.1 | 86.6±22.4 | 6.8±5.3 | 8.0±0.9 |
| Bailey, 2013^17^ | 102 weeks | Dapagliflozin2.5mg + MET | 137 | 55±25 | 51.1 | / | 84.9±17.8 | / | 7.99±0.9 |
|  |  | Dapagliflozin5mg + MET | 137 | 54.3±21.3 | 50.4 | / | 84.73±16.3 | / | 8.17±1.0 |
|  |  | Dapagliflozin10mg + MET | 135 | 52.7±23.7 | 57 | / | 86.28±17.5 | / | 7.92±0.8 |
|  |  | PBO + MET | 137 | 53.7±23.7 | 55.5 | / | 87.74±19.2 | / | 8.12±1.0 |
| Ferrannini, 2013^18^ | 78 weeks | Empagliflozin 10mg | 106 | 59 (30-76) | 46.2 | 28.9(20.3-39.2) | 82.9±16.4 | / | 7.89±0.87 |
|  |  | Empagliflozin 25mg | 109 | 59 (35-79) | 52.3 | 28.1(19.3-40.0) | 84.6±18.1 | / | 8.00±0.87 |
|  |  | MET | 56 | 58 (35-73) | 50.0 | 28.6(22.4-39.3) | 85.8±15.6 | / | 8.15±0.95 |
|  |  | Empagliflozin 10mg + MET | 166 | 60 (33-77) | 50.0 | 30.2(21.5-39.7) | 89.6±15.0 | / | 7.88±0.74 |
|  |  | Empagliflozin 25mg + MET | 166 | 60 (34-79) | 53.0 | 30.3(20.4-40.2) | 89.5±16.2 | / | 7.91±0.8 |
|  |  | Sitagliptin + MET | 56 | 60 (32-75) | 51.8 | 30.0(20.4-40.2) | 88.6±14.9 | / | 8.03±0.9 |
| Barnett, 2014-1^19^ | 52 weeks | Empagliflozin25mg CKD2 | 97 | 62.0±8.4 | 62.9 | 31.3±5.8 | 88.1±21.7 | / | 7.96±0.7 |
|  |  | Empagliflozin10mg CKD2 | 98 | 63.2±8.5 | 61.2 | 32.4±5.4 | 92.1±21.4 | / | 8.02±0.8 |
|  |  | PBO CKD2 | 95 | 62·6±8.1 | 58.9 | 30.8±5.6 | 86.0±20.0 | / | 8.09±0.8 |
| Barnett, 2014-2^19^ | 52 weeks | Empagliflozin25mg CKD3 | 187 | 64.6±8.9 | 57.2 | 30.2±5.3 | 83.2±19.5 | / | 8.02±0.8 |
|  |  | PBO CKD3 | 187 | 65.1±8.2 | 56.7 | 30.3±5.3 | 82.5±18.0 | / | 8.09±0.80 |
| Barnett, 2014-3^19^ | 52 weeks | Empagliflozin25mg CKD4 | 37 | 65.4±10.2 | 56.8 | 29.0±4.9 | 77.9±16.4 | / | 8.06±1.1 |
|  |  | PBO CKD4 | 37 | 62.9±11.9 | 51.4 | 31.8±6.0 | 84.1±21.1 | / | 8.16±1.0 |
| Rosenstock, 2014^20^ | 52 weeks | Empagliflozin 10mg + INS | 186 | 56.7±8.7 | 52.0 | 34.7±3.8 | 96.7±17.9 | / | 8.39±0.7 |
|  |  | Empagliflozin 25mg + INS | 189 | 58.0±9.4 | 44.0 | 35.0±4.0 | 95.9±17.3 | / | 8.29±0.7 |
|  |  | PBO + INS | 188 | 55.3±10.1 | 40.0 | 34.7±4.3 | 95.5±17.5 | / | 8.33±0.7 |
| Yale, 2014^21^ | 52 weeks | Canagliflozin 100mg | 90 | 69.5±8.2 | 64.4 | 32.4±5.5 | 90.5±18.4 | 15.6±7.4 | 7.9±0.9 |
|  |  | Canagliflozin 300mg | 89 | 67.9±8.2 | 53.9 | 33.4±6.5 | 90.2±18.1 | 17.0±7.8 | 8.0±0.8 |
|  |  | PBO | 90 | 68.2±8.4 | 63.3 | 33.1±6.5 | 92.8±17.4 | 16.4±10.1 | 8.0±0.9 |
| Bolinder, 2014^22^ | 2 years | Dapagliflozin10mg + MET | 89 | 60.6 ± 8.2 | 55.1 | 32.1 ± 3.9 | 92.1 ± 14.1 | 6.0 ± 4.5 | 7.19±0.44 |
|  |  | PBO + MET | 91 | 60.8 ± 6.9 | 56.0 | 31.7 ± 3.9 | 90.9 ± 13.7 | 5.5 ± 5.3 | 7.16±0.5 |
| Nauck, 2014^23^ | 104 weeks | Dapagliflozin + MET | 406 | 58.1±9.37 | 55.3 | 31.71±5.1 | / | / | 7.69±0.9 |
|  |  | Glipizide + MET | 408 | 58.6±9.80 | 54.9 | 31.23±5.1 | / | / | 7.74±0.9 |
| Kohan, 2014^24^ | 104 weeks | Dapagliflozin 5mg | 83 | 66±8.9 | 66.3 | / | 95.2±20.9 | 16.9±9.0 | 8.30±1.0 |
|  |  | Dapagliflozin 10mg | 85 | 68±7.7 | 65.9 | / | 93.2±17.3 | 18.2±10.1 | 8.22±1.0 |
|  |  | PBO | 84 | 67±8.6 | 63.1 | / | 89.6±20.0 | 15.7±9.5 | 8.53±1.3 |
| Leiter, 2014^25^ | 52 weeks | Dapagliflozin 10mg | 480 | 63.9±7.6 | 66.9 | 33.0±5.3 | 94.5±17.8 | 13.5±8.2 | 8.0±0.8 |
|  |  | PBO | 482 | 63.6±7.0 | 67.0 | 32.7±5.7 | 93.2±16.8 | 13.0±8.4 | 8.1±0.8 |
| Bode, 2015^26^ | 104 weeks | Canagliflozin 100mg | 241 | 64.3±6.5 | 51.5 | 31.4±4.4 | 88.4±15.6 | 12.3±7.8 | 7.8±0.8 |
|  |  | Canagliflozin 300mg | 236 | 63.4±6.0 | 54.7 | 31.5±4.6 | 88.8±17.1 | 11.3±7.2 | 7.7±0.8 |
|  |  | PBO | 237 | 63.2±6.2 | 60.3 | 31.8±4.7 | 91.1±17.5 | 11.4±7.3 | 7.8±0.8 |
| Cefalu, 2015^27^ | 52weeks | Dapagliflozin + OAD/INS | 455 | 62.8±7.0 | 67.9 | 32.6±5.9 | 92.6±20.5 | 12.6±8.7 | 8.18±0.8 |
|  |  | PBO + OAD/INS | 459 | 63.0±7.7 | 68.6 | 32.9±6.1 | 93.6±19.5 | 12.3±8.2 | 8.08±0.8 |
| Haering, 2015^28^ | 76 weeks | Empagliflozin 10mg | 225 | 57.0±9.2 | 50.2 | 28.3±5.4 | 77.1±18.3 | / | 8.1±0.8 |
|  |  | Empagliflozin 25mg | 216 | 57.4±9.3 | 52.8 | 28.3±5.5 | 77.5±18.8 | / | 8.1±0.8 |
|  |  | PBO | 225 | 56.9±9.2 | 49.8 | 27.9±4.9 | 76.2±16.9 | / | 8.2±0.8 |
| Lewin, 2015^29^ | 52 weeks | Empagliflozin  25mg + linagliptin | 134 | 54.2±10.0 | 52.2 | 31.8±5.3 | 87.9±18.2 | / | 7.99±1.0 |
|  |  | Empagliflozin  10mg + linagliptin | 135 | 55.2±9.8 | 54.1 | 31.5±5.6 | 87.3±18.4 | / | 8.04±1.0 |
|  |  | Empagliflozin 25mg | 133 | 56.0±9.3 | 57.9 | 31.2±5.7 | 86.7±19.7 | / | 7.99±1.0 |
|  |  | Empagliflozin 10mg | 132 | 53.9±10.5 | 48.5 | 31.5±5.7 | 87.8±24.0 | / | 8.05±1.0 |
|  |  | Linagliptin 5mg | 133 | 53.8±11.5 | 56.4 | 31.9±5.9 | 89.5±20.1 | / | 8.05±0.9 |
| Rosenstock, 2015^30^ | 78 weeks | Empagliflozin 10mg | 169 | 58.6 ± 9.8 | 55.0 | 32.1 ± 5.8 | 91.6 ± 20.1 | / | 8.3 ± 0.8 |
|  |  | Empagliflozin 25mg | 155 | 59.9 ± 10.5 | 60.0 | 32.7 ± 5.9 | 94.7 ± 20.7 | / | 8.3 ± 0.8 |
|  |  | PBO | 170 | 58.1 ± 9.4 | 53.0 | 31.8 ± 6.0 | 90.5 ± 22.5 | / | 8.2 ± 0.8 |
| Leiter, 2015^31^ | 104 weeks | Canagliflozin 100mg | 483 | 56.4±9.5 | 52.2 | 31.0±5.3 | 86.9±20.1 | 6.5±5.5 | 7.8±0.8 |
|  |  | Canagliflozin 300mg | 485 | 55.8±9.2 | 49.7 | 31.2±5.4 | 86.6±19.5 | 6.7±5.5 | 7.8±0.8 |
|  |  | Glimepiride | 482 | 56.3±9.0 | 54.6 | 30.9±5.5 | 86.5±19.8 | 6.6±5.0 | 7.8±0.8 |
| Bailey, 2015^32^ | 102 weeks | Dapagliflozin2.5mg | 65 | 53.0±11.7 | 55.4 | / | 90.8±22.8 | 2.1±3.2 | 7.92±0.9 |
|  |  | Dapagliflozin 5mg | 64 | 52.6±10.9 | 48.4 | / | 87.6±17.1 | 1.0±1.6 | 7.86±0.9 |
|  |  | Dapagliflozin10mg | 70 | 50.6±10.0 | 48.6 | / | 94.2±18.7 | 2.3±3.7 | 8.01±1.0 |
|  |  | PBO + low-dose MET | 75 | 52.7±10.3 | 41.3 | / | 88.8±19.0 | 2.1±3.1 | 7.84±0.9 |
| Dagogo-Jack, 2017^33^ | 52 weeks | Ertugliflozin 5mg | 156 | 59.2±9.3 | 51.9 | 31.2±5.5 | 87.6±18.6 | 9.9±6.1 | 8.1±0.9 |
|  |  | Ertugliflozin 15mg | 153 | 59.7±8.6 | 53.6 | 30.9±6.1 | 86.6±19.5 | 9.2±5.3 | 8.0±0.8 |
|  |  | PBO | 153 | 58.3±9.2 | 65.4 | 30.3±6.4 | 86.4±20.8 | 9.4±5.6 | 8.0±0.9 |
| Müller-Wieland, 2018^34^ | 52 weeks | Dapagliflozin + MET | 314 | 57.4±9.4 | 64.3 | 33.1±5.2 | 97.7±18.9 | 6.9±5.2 | 8.3±0.7 |
|  |  | Dapagliflozin + Saxagliptin + MET | 312 | 59.2±7.9 | 60.9 | 32.5±5.1 | 95.3±17.4 | 7.3±5.9 | 8.3±0.7 |
|  |  | Glimepiride + MET | 313 | 58.6±8.4 | 66.5 | 33.0±5.1 | 97.3±17.9 | 6.7±5.1 | 8.3±0.8 |
| Silvina Gallo, 2019^35^ | 104 weeks | Ertugliflozin 5 mg | 207 | 56.6±8.2 | 46.9 | 31.1±4.8 | 84.8±17.2 | 7.87±6.08 | 8.06±0.9 |
|  |  | Ertugliflozin 15 mg | 205 | 56.9±9.4 | 45.4 | 31.4±4.6 | 85.3±16.5 | 8.07±5.52 | 8.13±0.9 |
|  |  | PBO | 209 | 56.5±8.7 | 46.9 | 31.0±4.7 | 84.5±17.1 | 8.04±6.34 | 8.17±0.9 |
| Active agent vs placebo treatment in CVOTs or ROTs | | | | | | | | | |
| Pfeffer, 2015^36^  (ELIXA) | 25 months | Lixisenatide | 3034 | 59.9±9.7 | 69.6 | 30.1±5.6 | 84.6±19.2 | 9.2±8.2 | 7.7 ± 1.3 |
|  |  | PBO | 3034 | 60.6 ± 9.6 | 69.1 | 30.2 ± 5.8 | 85.1±19.6 | 9.4±8.3 | 7.6±1.3 |
| Zinman, 2015^37^  (EMPA-REG) | 3.1 years | Empagliflozin 10mg | 2345 | 63.0 ± 8.6 | 70.5 | 30.6±5.2 | 85.9±18.8 | / | 8.07±0.8 |
|  |  | Empagliflozin 25mg | 2342 | 63.2±8.6 | 71.2 | 30.6±5.3 | 86.5±19.0 | / | 8.06±0.8 |
|  |  | PBO | 2333 | 63.2 ± 8.8 | 72.0 | 30.7±5.2 | 86.6±19.1 | / | 8.08± 0.8 |
| Marso, 2016^38^  (LEADER) | 3.8 years | Liraglutide | 4668 | 64.2 ± 7.2 | 64.5 | 32.5±6.3 | 91.9±21.2 | 12.8±8.0 | 8.7±1.6 |
|  |  | PBO | 4672 | 64.2 ± 7.2 | 64.9 | 32.5±6.3 | 91.6±20.8 | 12.9±8.1 | 8.7±1.5 |
| Marso, 2016^39^  (SUSTAIN6) | 2.1 years | Semaglutide 0.5 mg | 826 | 64.6 ± 7.3 | 40.1 | / | 91.8±20.3 | 14.3±8.2 | 8.7±1.4 |
|  |  | Semaglutide 1.0 mg | 822 | 64.7 ± 7.1 | 37.0 | / | 92.9±21.1 | 14.1±8.2 | 8.7±1.5 |
|  |  | PBO 0.5 mg | 824 | 64.8 ± 7.6 | 41.5 | / | 91.8±20.3 | 14.0±8.5 | 8.7±1.5 |
|  |  | PBO 10 mg | 825 | 64.4 ± 7.5 | 38.5 | / | 91.9±20.8 | 13.2 ± 7.4 | 8.7 ± 1.5 |
| Holman,2017^40^  (EXSCEL) | 3.2 years | Exenatide | 7356 | / | 62.0 | / | / | / | / |
|  |  | PBO | 7396 | / | 62.0 | / | / | / | / |
| Neal, 2017^41^  (CANVAS) | 188 weeks | Canagliflozin | 5644 | 63.2±8.3 | 64.9 | 31.9±5.9 | / | 13.5±7.7 | 8.2±0.9 |
|  |  | PBO | 4231 | 63.4 ± 8.2 | 63.3 | 32.0 ± 6.0 | / | 13.7 ± 7.8 | 8.2±0.9 |
| Hernandez,2018^42^ | 28months | Albiglutide | 4731 | 64.1±8.7 | 70.0 | 32.3±5.9 | / | 14.1±8.6 | 8.76±1.5 |
| (HARMONY) |  | PBO | 4732 | 64.2±8.7 | 69.0 | 32.3±5.9 | / | 14.2±8.9 | 8.72±1.5 |
| Wiviott,2018^43^ | 206weeks | Dapagliflozin | 8582 | 63.9±6.8 | 63.1 | 32.1±6.0 | / | 11.0±5 | 8.3±1.2 |
| (DECLARE-TIMI 58) |  | PBO | 8578 | 64.0±6.8 | 62.1 | 32.0±6.1 | / | 10.1±5 | 8.3±1.2 |
| Perkovic, 2019^44^ | 2.62 years | Canagliflozin | 2202 | 62.9±9.2 | 65.4 | 31.4±6.2 | / | 15.5±8.7 | 8.3±1.3 |
| (CREDENCE) |  | PBO | 2199 | 63.2±9.2 | 66.7 | 31.3±6.2 | / | 16.0±8.6 | 8.3±1.3 |
| Gerstein, 2019^45^ | 5.4 years | Dulaglutide | 4949 | 66.2±6.5 | 53.4 | 32.3±5.7 | / | 10.5±7.3 | 7.3±1.1 |
| (REWIND) |  | PBO | 4952 | 66.2±6.5 | 53.9 | 32.3±5.8 | / | 10.6±7.2 | 7.4±1.1 |
| Marx, 2019^46^  (CAROLINA) | 6.3 years | Linagliptin | 3023 | 63.9±9.5 | 60.8 | 30.2±5.2 | 84.3±18.0 | 6.3 (3.0, 11.1) | 7.2±0.6 |
|  |  | Glimepiride | 3010 | 64.2±9.5 | 59.2 | 30.0±5.1 | 83.6±17.9 | 6.2 (2.9, 10.9) | 7.2±0.16 |

Values are expressed as absolute number or mean±standard deviationor mean (minimal value, maximal value). MET, metformin; SU, sulfonylurea TZD,thiazolidinedione; PIO, pioglitazone;PBO, placebo; OAD, oral anti-diabetic drugs.

UKPDS: The UK Prospective Diabetes Study

VADT: Veterans Affairs Diabetes Trial

RECORD: Rosiglitazone Evaluated for Cardiovascular Outcomes in Oral Agent Combination Therapy for Type 2 Diabetes

ELIXA: Lixisenatide in Patients with Type 2 Diabetes and Acute Coronary Syndrome

EMPA-REG: Empagliflozin, Cardiovascular Outcomes, and Mortality in Type 2 Diabetes

LEADER: Liraglutide and Cardiovascular Outcomes in Type 2 Diabetes

SUSTAIN6: Semaglutide and Cardiovascular Outcomes in Patients with Type 2 Diabetes

EXSCEL: Exenatide Study of Cardiovascular Event Lowering

CANVAS: Canagliflozin and Cardiovascular and Renal Events in Type 2 Diabetes

HARMONY: Albiglutide and Cardiovascular Outcomes in Patients with Type 2 Diabetes and Cardiovascular Disease

DECLARE-TIMI 58: The Dapagliflozin Effect on Cardiovascular Events-Thrombolysis in Myocardial Infarction

CREDENCE: Canagliflozin and Renal Outcomes in Type 2 Diabetes and Nephropathy

REWIND: Dulaglutide and Cardiovascular Outcomes in Type 2 Diabetes

DUAL VIII: Durability of Insulin Degludec plus Liraglutide versus Insulin Glargine U100 as Initial Injectable Therapy in Type 2 Diabetes

CAROLINA: Cardiovascular Outcome Study of Linagliptin versus Glimepiride in Patients with Type 2 Diabetes

**Table S2. Risk of bias for included studies in this meta-analysis.**

| **Author, year** | **Adequate randomization sequence generation** | **Adequate**  **allocation concealment** | **Blinding**  **of participants and caregivers** | **Binding of**  **outcome assessors**  **and adjudicators** | **Free of infrequent**  **missing outcome data** | **Free of selective outcome reporting** | **Free of other bias** |
| --- | --- | --- | --- | --- | --- | --- | --- |
| **Intensive vs standard treatment** | | | | | | | |
| Turner, 1998^1^  (UKPDS 33) | **Definitely yes**  Using a computer-generated therapy allocation | **Probably no**  Randomized, open label | **Definitely no**  Open label | **Definitely yes** | **Definitely yes**  There were 4.47% (122/2729) and 3.95% (45/1138) patients in intensive and standard groups with missing outcome data, respectively; missing outcome data were generally balanced across treatment groups, with similar reasons for missing data across groups | **Definitely yes** | **Probably yes**  Generally balanced baseline  characteristics across groups |
| Duckworth, 2009^2^  (VADT) | **Definitely yes**  Using randomization codes | **Probably no**  Randomized, open label | **Definitely no**  Open label | **Definitely yes** | **Probably no**  There were 13.5% (120/892) and 15.5% (139/899) patients in intensive and standard groups with missing outcome data, respectively; missing outcome data were generally balanced across treatment groups, with similar reasons for missing data across groups | **Definitely yes** | **Probably yes**  Generally balanced baseline  characteristics across groups |
| Home, 2009^3^  (RECORD) | **Definitely yes**  Using random-permuted blocks | **Probably no**  Randomized, open label | **Definitely no**  Open label | **Definitely yes** | **Probably no**  There were 17.5% (385/2200) and 19.3% (429/2227) patients in intensive and standard groups with missing outcome data, respectively; missing outcome data were generally balanced across treatment groups, with similar reasons for missing data across groups | **Definitely yes** | **Probably yes**  Generally balanced baseline  characteristics across groups |
| Active agent vs placebo treatment in efficacy evaluation | | | | | | | |
| Insulin | | | | | | | |
| Hollander, 2008^4^ | **Definitely yes**  Using a telephone randomization system  prepared by Clinical Trial Supplies | **Probably no**  Randomized, open label | **Definitely no**  Open label | **Definitely yes** | **Probably no**  There were 19.9% (43/216) and 21.5% (23/107) patients in detemir and glargine groups with missing outcome data, respectively; missing outcome data were generally balanced across treatment groups, with similar reasons for missing data across groups | **Definitely yes** | **Probably yes**  Generally balanced baseline  characteristics across groups |
| Rosenstock, 2008^5^ | **Definitely yes**  Using an automatic  telephone response system | **Probably no**  Randomized, open label | **Definitely no**  Open label | **Definitely yes** | **Probably no**  There were 20.1% (60/291) and 13.4% (39/291) patients in detemir and glargine groups with missing outcome data, respectively; missing outcome data were generally balanced across treatment groups, with similar reasons for missing data across groups | **Definitely yes** | **Probably yes**  Generally balanced baseline  characteristics across groups |
| Rosenstock, 2009^5^ | **Definitely yes**  Using the centralized interactive voice response system (IVRS) | **Probably no**  Randomized, open label | **Definitely no**  Open label | **Definitely yes** | **Definitely no**  There were 27.1% (139/513) and 27.8% (140/504) patients in detemir and glargine groups with missing outcome data, respectively; missing outcome data were generally balanced across treatment groups, with similar reasons for missing data across groups | **Definitely yes** | **Probably yes**  Generally balanced baseline  characteristics across groups |
| Aroda, 2019^7^  (DUAL VIII) | **Definitely yes**  Using an interactive web response system anda simple sequential allocation randomization schedulewith block size of four | **Probably no**  Randomized, open label | **Definitely no**  Open label | **Definitely yes** | **Definitely no**  There were 38.1% (193/506) and 67.2% (340/506) patients in IDegLira and IGlar U100 groups with missing outcome data, respectively; missing outcome data were generally balanced across treatment groups, with similar reasons for missing data across groups | **Definitely yes** | **Probably yes**  Generally balanced baseline  characteristics across groups |
| GLP-1RA | | | | | | | |
| Ahren, 2014^8^ | **Probably yes**  Randomized,  double-blind | **Probably yes**  Randomized, double-blind | **Definitely yes**  Double-blind  (participant,  investigator) | **Definitely yes** | **Definitely no**  There were 31.7% (100/315) and 32.9% (242/734) patients in GLP-1RA and placebo groups with missing outcome data,  respectively, with similar reasons for missing data across groups | **Definitely yes** | **Probably yes**  Generally balanced baseline  characteristics across groups |
| Weinstock, 2015^9^ | **Probably yes**  Randomized,  double-blind | **Probably yes**  Randomized, double-blind | **Probably yes**  Double-blind  (details not reported) | **Definitely yes** | **Definitely no**  There were 38.0% (230/606) and 42.9% (211/492) patients in GLP-1RA and placebo groups with missing outcome data, respectively | **Definitely yes** | **Probably yes**  Generally balanced baseline  characteristics across groups |
| Davies, 2015^10^ | **Definitely yes**  Using a centralized manner via an interactive voice/web response system | **Probably yes**  Randomized, double-blind | **Definitely yes**  Double-blind  (participant,  investigator) | **Definitely yes** | **Definitely no**  There were 26.8% (170/634) and 34.0% (72/212) patients in GLP-1RA and placebo groups with missing outcome data, respectively, with similar reasons for missing data across groups | **Definitely yes** | **Probably yes**  Generally balanced baseline  characteristics across groups |
| Home, 2015^11^ | **Definitely yes**  Using an interactive voice response system | **Probably yes**  Randomized, double-blind | **Definitely yes**  Double-blind  (participant,  investigator) | **Definitely yes** | **Definitely no**  There were 18.1% (51/281) and 22.0% (89/404) patients in GLP-1RA and placebo groups with missing outcome data,  respectively, with similar reasons for missing data across groups | **Definitely yes** | **Probably yes**  Generally balanced baseline  characteristics across groups |
| SGLT2 inhibitors | | | | | | | |
| Wilding, 2012^12^ | **Definitely yes**  Using a computer-generated, stratified, block-randomization schedule | **Probably yes**  Randomized, double-blind | **Definitely yes**  Double-blind  (participant,  investigator) | **Definitely yes** | **Probably no**  There were 11.0% (67/610) and 14.7% (29/197) patients in SGLT-2i and placebo groups with missing outcome data, respectively; missing outcome data were generally balanced across treatment groups, with similar reasons for missing data across groups | **Definitely yes** | **Probably yes**  Generally balanced baseline  characteristics across groups |
| Rosenstock, 2012^13^ | **Probably yes**  Randomized,  double-blind | **Probably yes**  Randomized, double-blind | **Definitely yes**  Double-blind  (participant,  investigator) | **Definitely yes** | **Probably no**  There were 10.7% (30/281) and 16.5% (23/139) patients in SGLT-2i and placebo groups with missing outcome data, respectively; missing outcome data were generally balanced across treatment groups, with similar reasons for missing data across groups | **Definitely yes** | **Probably yes**  Generally balanced baseline  characteristics across groups |
| Schernthaner, 2013^14^ | **Definitely yes**  Using an Interactive Voice Response System/  Interactive Web Response System | **Probably yes**  Randomized, double-blind | **Definitely yes**  Double-blind  (participant,  investigator) | **Definitely yes** | **Definitely no**  There were 32.5% (123/378) and 44.4% (123/378) patients in SGLT-2i and placebo groups with missing outcome data, respectively; missing outcome data were generally balanced across treatment groups, with similar reasons for missing data across groups | **Definitely yes** | **Probably yes**  Generally balanced baseline  characteristics across groups |
| Wilding, 2013^15^ | **Definitely yes**  Using an Interactive Voice Response System/  Interactive Web Response System | **Probably yes**  Randomized, double-blind | **Definitely yes**  Double-blind  (participant,  investigator) | **Definitely yes** | **Definitely no**  There were 29.7% (93/313) and42.3 % (66/156) patients in SGLT-2i and placebo groups with missing outcome data, respectively; missing outcome data were generally balanced across treatment groups, with similar reasons for missing data | **Definitely yes** | **Probably yes**  Generally balanced baseline  characteristics across groups |
| Lavalle-González, 2013^16^ | **Definitely yes**  Using a computer-generated schedule | **Probably yes**  Randomized, double-blind | **Definitely yes**  Double-blind  (participant,  investigator) | **Definitely yes** | **Definitely no**  There were 18.8% (138/735) and 23.0% (126/549) patients in SGLT-2i and placebo groups with missing outcome data, respectively; missing outcome data were generally balanced across treatment groups, with similar reasons for missing data across groups | **Definitely yes** | **Probably yes**  Generally balanced baseline  characteristics across groups |
| Bailey, 2013^17^ | **Probably yes**  Randomized,  double-blind | **Probably yes**  Randomized,  double-blind | **Probably yes**  Double-blind  (details not reported) | **Definitely yes** | **Definitely no**  There were 35.0% (143/7409) and 46.7% (64/137) patients in SGLT-2i and placebo groups with missing outcome data, respectively; missing outcome data were generally balanced across treatment groups, with similar reasons for missing data across groups | **Definitely yes** | **Probably yes**  Generally balanced baseline  characteristics across groups |
| Ferrannini, 2013^18^ | **Probably yes**  Randomized | **Probably no**  Randomized, open label | **Definitely no**  Open label | **Definitely yes** | **Probably no**  There were 17.5% (48/275) and 12.7% (7/55) patients in SGLT-2i and placebo groups with missing outcome data, respectively; missing outcome data were generally balanced across treatment groups, with similar reasons for missing data across groups | **Definitely yes** | **Probably yes**  Generally balanced baseline  characteristics across groups |
| Barnett, 2014-1^19^ | **Definitely yes**  Using a computer-generated random sequence | **Probably yes**  Randomized, double-blind | **Definitely yes**  Double-blind  (participant,  investigator) | **Definitely yes** | **Probably no**  There were 9.2% (18/195) and 8.2% (8/97) patients in SGLT-2i and placebo groups with missing outcome data, respectively; missing outcome data were generally balanced across treatment groups, with similar reasons for missing data across groups | **Definitely yes** | **Probably yes**  Generally balanced baseline  characteristics across groups |
| Barnett, 2014-2^19^ | **Definitely yes**  Using a computer-generated random sequence | **Probably yes**  Randomized, double-blind | **Definitely yes**  Double-blind  (participant,  investigator) | **Definitely yes** | **Probably no**  There were 12.2% (23/188) and 11.2% (21/187) patients in SGLT-2i and placebo groups with missing outcome data, respectively; missing outcome data were generally balanced across treatment groups, with similar reasons for missing data across groups | **Definitely yes** | **Probably yes**  Generally balanced baseline  characteristics across groups |
| Barnett, 2014-3^19^ | **Definitely yes**  Using a computer-generated random sequence | **Probably yes**  Randomized, double-blind | **Definitely yes**  Double-blind  (participant,  investigator) | **Definitely yes** | **Definitely no**  There were 29.7% (11/37) and 32.4% (12/37) patients in SGLT-2i and placebo groups with missing outcome data, respectively; missing outcome data were generally balanced across treatment groups, with similar reasons for missing data across groups | **Definitely yes** | **Probably yes**  Generally balanced baseline  characteristics across groups |
| Rosenstock, 2014^20^ | **Definitely yes**  Using an interactive voice-and web-response system | **Probably yes**  Randomized, double-blind | **Definitely yes**  Double-blind  (participant,  investigator) | **Definitely yes** | **Probably no**  There were 15.6% (59/377) and 16.9% (32/189) patients in SGLT-2i and placebo groups with missing outcome data, respectively; missing outcome data were generally balanced across treatment groups, with similar reasons for missing data across groups | **Definitely yes** | **Probably yes**  Generally balanced baseline  characteristics across groups |
| Yale, 2014^21^ | **Probably yes**  Randomized | **Probably yes**  Randomized,  double-blind | **Probably yes**  Double-blind  (details not reported) | **Definitely yes** | **Probably no**  There were 20.1% (36/179) and 28.9% (26/90) patients in SGLT-2i and placebo groups with missing outcome data, respectively; missing outcome data were generally balanced across treatment groups, with similar reasons for missing data across groups | **Definitely yes** | **Probably yes**  Generally balanced baseline  characteristics across groups |
| Bolinder, 2014^22^ | **Definitely yes**  Using a predefined computer-generated randomization scheme provided by AstraZeneca | **Probably yes**  Randomized,  double-blind | **Definitely yes**  Double-blind  (participant,  investigator) | **Definitely yes** | **Probably no**  There were 24.2% (22/91) and 22.0% (20/91) patients in SGLT-2i and placebo groups with missing outcome data, respectively; missing outcome data were generally balanced across treatment groups, with similar reasons for missing data across groups | **Definitely yes** | **Probably yes**  Generally balanced baseline  characteristics across groups |
| Nauck, 2014^23^ | **Probably yes**  Randomized | **Probably yes**  Randomized, double-blind | **Probably yes**  Double-blind  (details not reported) | **Definitely yes** | **Definitely no**  There were 43.8% (178/406) and 50.0% (204/408) patients in SGLT-2i and placebo groups with missing outcome data, respectively; missing outcome data were generally balanced across treatment groups, with similar reasons for missing data across groups | **Definitely yes** | **Probably yes**  Generally balanced baseline  characteristics across groups |
| Kohan, 2014^24^ | **Probably yes**  Randomized | **Probably yes**  Randomized,  double-blind | **Probably yes**  Double-blind  (details not reported) | **Definitely yes** | **Definitely no**  There were 42.9% (72/168) and 48.8% (41/84) patients in SGLT-2i and placebo groups with missing outcome data, respectively; missing outcome data were generally balanced across treatment groups, with similar reasons for missing data across groups | **Definitely yes** | **Probably yes**  Generally balanced baseline  characteristics across groups |
| Leiter, 2014^25^ | **Definitely yes**  Using a computer-generated random sequence via an interactive voice or web response system | **Probably yes**  Randomized,  double-blind | **Probably yes**  Double-blind  (details not reported) | **Definitely yes** | **Definitely no**  There were 31.2% (302/968) and 34.9% (168/482) patients in SGLT-2i and placebo groups with missing outcome data, respectively; missing outcome data were generally balanced across treatment groups, with similar reasons for missing data across groups | **Definitely yes** | **Probably yes**  Generally balanced baseline  characteristics across groups |
| Bode, 2015^26^ | **Probably yes**  Randomized,  double-blind | **Probably yes**  Randomized, double-blind | **Definitely yes**  Double-blind  (participant,  investigator) | **Definitely yes** | **Probably no**  There were 15.4% (69/447) and 14.8% (35/237) patients in SGLT-2i and placebo groups with missing outcome data, respectively; missing outcome data were generally balanced across treatment groups, with similar reasons for missing data across groups | **Definitely yes** | **Probably yes**  Generally balanced baseline  characteristics across groups |
| Cefalu, 2015^27^ | **Probably yes**  Randomized,  double-blind | **Probably yes**  Randomized, double-blind | **Definitely yes**  Double-blind  (participant,  investigator) | **Definitely yes** | **Definitely yes**  There were 1.1% (5/460) and 0.6% (3/462) patients in SGLT-2i and placebo groups with missing outcome data, respectively; missing outcome data were generally balanced across treatment groups, with similar reasons for missing data across groups | **Definitely yes** | **Probably yes**  Generally balanced baseline  characteristics across groups |
| Haering, 2015^28^ | **Probably yes**  Randomized,  double-blind | **Probably yes**  Randomized, double-blind | **Definitely yes**  Double-blind  (participant,  investigator) | **Definitely yes** | **Definitely no**  There were 32.4% (144/444) and 43.6% (98/225) patients in SGLT-2i and placebo groups with missing outcome data, respectively, with similar reasons for missing data across groups | **Definitely yes** | **Probably yes**  Generally balanced baseline  characteristics across groups |
| Lewin, 2015^29^ | **Probably yes**  Randomized,  double-blind | **Probably yes**  Randomized, double-blind | **Probably yes**  Double-blind  (details not reported) | **Definitely yes** | **Probably no**  There were 16.7% (45/269) and 14.1% (19/135) patients in empagliflozin and linagliptin groups with missing outcome data, respectively; missing outcome data were generally balanced across treatment groups, with similar reasons for missing data across groups | **Definitely yes** | **Probably yes**  Generally balanced baseline  characteristics across groups |
| Rosenstock, 2015^30^ | **Definitely yes**  Using a third-party interactive voice and web response system | **Probably yes**  Randomized, double-blind | **Definitely yes**  Double-blind  (participant,  investigator) | **Definitely yes** | **Definitely no**  There were 25.3% (82/324) and 30.6% (52/170) patients in SGLT-2i and placebo groups with missing outcome data, respectively; missing outcome data were generally balanced across treatment groups, with similar reasons for missing data across groups | **Definitely yes** | **Probably yes**  Generally balanced baseline  characteristics across groups |
| Leiter, 2015^31^ | **Probably yes**  Randomized,  double-blind | **Probably yes**  Randomized, double-blind | **Definitely yes**  Double-blind  (participant,  investigator) | **Definitely yes** | **Definitely no**  There were 31.2% (302/968) and 34.9% (168/482) patients in SGLT-2i and placebo groups with missing outcome data, respectively; missing outcome data were generally balanced across treatment groups, with similar reasons for missing data across groups | **Definitely yes** | **Probably yes**  Generally balanced baseline  characteristics across groups |
| Bailey, 2015^32^ | **Definitely yes**  Using an Interactive  Voice Response System | **Probably yes**  Randomized, double-blind | **Definitely yes**  Double-blind  (participant,  investigator) | **Definitely yes** | **Probably no**  There were 16.6% (33/199) and 17.3% (13/75) patients in SGLT-2i and placebo groups with missing outcome data, respectively; missing outcome data were generally balanced across treatment groups, with similar reasons for missing data across groups | **Definitely yes** | **Probably yes**  Generally balanced baseline  characteristics across groups |
| Dagogo-Jack, 2017^33^ | **Definitely yes**  Using a computer-generated randomization schedule | **Probably yes**  Randomized, double-blind | **Definitely yes**  Double-blind  (participant,  investigator) | **Definitely yes** | **Probably no**  There were 11.6% (35/301) and 14.4% (22/153) patients in SGLT-2i and placebo groups with missing outcome data, respectively; missing outcome data were generally balanced across treatment groups, with similar reasons for missing data across groups | **Definitely yes** | **Probably yes**  Generally balanced baseline  characteristics across groups |
| Müller-Wieland, 2018^34^ | **Probably yes**  Randomized,  double-blind | **Probably yes**  Randomized,  double-blind | **Probably yes**  Double-blind  (details not reported) | **Definitely yes** | **Probably no**  There were 7.5% (47/626) and 8.0% (25/313) patients in dapagliflozinand glimepiride groups with missing outcome data, respectively; missing outcome data were generally balanced across treatment groups, with similar reasons for missing data across groups | **Definitely yes** | **Probably yes**  Generally balanced baseline  characteristics across groups |
| Gallo, 2019^35^ | **Probably yes**  Randomized,  double-blind | **Probably yes**  Randomized,  double-blind | **Probably yes**  Double-blind  (details not reported) | **Definitely yes** | **Probably no**  There were 18.9% (78/412) and 25.8% (54/209) patients in dapagliflozinand glimepiride groups with missing outcome data, respectively; missing outcome data were generally balanced across treatment groups, with similar reasons for missing data across groups | **Definitely yes** | **Probably yes**  Generally balanced baseline  characteristics across groups |
| Active agent vs placebo treatment in CVOTs or ROTs | | | | | | | |
| Pfeffer, 2015^36^  (ELIXA) | **Definitely yes**  Using a centralized assignment system | **Probably yes**  Randomized, double-blind | **Definitely yes**  Double-blind  (participant,  investigator) | **Definitely yes** | **Probably yes**  There were 5.7% (204/3034) and 7.8% (231/3034) patients in active agent and placebo groups with missing outcome data, respectively; missing outcome data were generally balanced across treatment groups, with similar reasons for missing data across groups | **Definitely yes** | **Probably yes**  Generally balanced baseline  characteristics across groups |
| Zinman, 2015^37^  (EMPA-REG) | **Definitely yes**  Using a computer-generated random-sequence and interactive voice-and web-response system | **Probably yes**  Randomized, double-blind | **Definitely yes**  Double-blind  (participant,  investigator) | **Definitely yes** | **Definitely yes**  There were 3.1% (144/4687) and 2.9% (67/2333) patients in active agent and placebo groups with missing outcome data, respectively; missing outcome data were generally balanced across treatment groups, with similar reasons for missing data across groups | **Definitely yes** | **Probably yes**  Generally balanced baseline  characteristics across groups |
| Marso, 2016^38^  (LEADER) | **Definitely yes**  Using the interactive voice/web response system | **Probably yes**  Randomized, double-blind | **Probably yes**  Double-blind  (details not reported) | **Definitely yes** | **Definitely yes**  There were 1.5% (25/1648) and 2.4% (40/1649) patients in active agent and placebo groups with missing outcome data, respectively; missing outcome data were generally balanced across treatment groups, with similar reasons for missing data across groups | **Definitely yes** | **Probably yes**  Generally balanced baseline  characteristics across groups |
| Marso, 2016^39^  (SUSTAIN6) | **Probably yes**  Randomized,  double-blind | **Probably yes**  Randomized, double-blind | **Probably yes**  Double-blind  (details not reported) | **Definitely yes** | **Definitely yes**  There were 3.0% (139/4668) and 3.4% (159/4672) patients in active agent and placebo groups with missing outcome data, respectively; missing outcome data were generally balanced across treatment groups, with similar reasons for missing data across groups | **Definitely yes** | **Probably yes**  Generally balanced baseline  characteristics across groups |
| Holman,2017^40^ (EXSCEL) | **Definitely yes**  Using an interactive voice-response system | **Probably yes**  Randomized, double-blind | **Definitely yes**  Double-blind  (participant,  investigator) | **Definitely yes** | **Definitely yes**  There were 3.6% (262/7356) and 4.1% (303/7396) patients in active agent and placebo groups with missing outcome data, respectively; missing outcome data were generally balanced across treatment groups, with similar reasons for missing data across groups | **Definitely yes** | **Probably yes**  Generally balanced baseline  characteristics across groups |
| Neal, 2017^41^  (CANVAS) | **Definitely yes**  Using an interactive web-based response system | **Probably yes**  Randomized, double-blind | **Definitely yes**  Double-blind  (participant,  investigator) | **Definitely yes** | **Definitely yes**  There were 4.2% (184/5795) and 3.9% (224/4347) patients in active agent and placebo groups with missing outcome data, respectively; missing outcome data were generally balanced across treatment groups, with similar reasons for missing data across groups | **Definitely yes** | **Probably yes**  Generally balanced baseline  characteristics across groups |
| Hernandez, 2018^42^  (HARMONY) | **Definitely yes**  Using a computer-generated randomization code | **Probably yes**  Randomized, double-blind | **Definitely yes**  Double-blind  (participant,  investigator) | **Definitely yes** | **Definitely yes**  There were 2.5% (111/4371) and 3.3% (154/4732) patients in active agent and placebo groups with missing outcome data, respectively; missing outcome data were generally balanced across treatment groups, with similar reasons for missing data across groups | **Definitely yes** | **Probably yes**  Generally balanced baseline  characteristics across groups |
| Wiviott, 2018^43^  (DECLARE-TIMI 58) | **Definitely yes**  Using an interactive-voice/web response system | **Probably yes**  Randomized, double-blind | **Definitely yes**  Double-blind  (participant,  investigator) | **Definitely yes** | **Definitely yes**  There were 1.3% (109/8582) and 1.7% (145/8578) patients in active agent and placebo groups with missing outcome data, respectively; missing outcome data were generally balanced across treatment groups, with similar reasons for missing data across groups | **Definitely yes** | **Probably yes**  Generally balanced baseline  characteristics across groups |
| Perkovic, 2019^44^ (CREDENCE) | **Definitely yes**  Using an interactive web response system with a computer-generated randomization schedule | **Probably yes**  Randomized, double-blind | **Definitely yes**  Double-blind  (participant,  investigator) | **Definitely yes** | **Definitely yes**  There were 0.68% (15/2202) and 1.1% (25/2174) patients in active agent and placebo groups with missing outcome data, respectively; missing outcome data were generally balanced across treatment groups, with similar reasons for missing data across groups | **Definitely yes** | **Probably yes**  Generally balanced baseline  characteristics across groups |
| Gerstein, 2019^45^  (REWIND) | **Probably yes**  Randomized,  double-blind | **Probably yes**  Randomized, double-blind | **Definitely yes**  Double-blind  (participant,  investigator) | **Definitely yes** | **Definitely yes**  There were 2.7% (132/4949) and 3.2% (159/4935) patients in active agent and placebo groups with missing outcome data, respectively; missing outcome data were generally balanced across treatment groups, with similar reasons for missing data across groups | **Definitely yes** | **Probably yes**  Generally balanced baseline  characteristics across groups |
| Rosenstock, 2019^46^  (CAROLINA) | **Definitely yes**  Using a computer-generated random sequence and an interactive voice and web response system | **Probably yes**  Randomized, double-blind | **Definitely yes**  Double-blind  (participant,  investigator) | **Definitely yes** | **Definitely no**  There were 37.3% (1127/3023) and 39.1% (1178/3010) patients in SGLT-2 inhibitor and control groups with missing outcome data, respectively | **Definitely yes** | **Probably yes**  Generally balanced baseline  characteristics across groups |

UKPDS: The UK Prospective Diabetes Study

VADT: Veterans Affairs Diabetes Trial

RECORD: Rosiglitazone Evaluated for Cardiovascular Outcomes in Oral Agent Combination Therapy for Type 2 Diabetes

ELIXA: Lixisenatide in Patients with Type 2 Diabetes and Acute Coronary Syndrome

EMPA-REG: Empagliflozin, Cardiovascular Outcomes, and Mortality in Type 2 Diabetes

LEADER: Liraglutide and Cardiovascular Outcomes in Type 2 Diabetes

SUSTAIN6: Semaglutide and Cardiovascular Outcomes in Patients with Type 2 Diabetes

EXSCEL: Exenatide Study of Cardiovascular Event Lowering

CANVAS: Canagliflozin and Cardiovascular and Renal Events in Type 2 Diabetes

HARMONY: Albiglutide and Cardiovascular Outcomes in Patients with Type 2 Diabetes and Cardiovascular Disease

DECLARE-TIMI 58: The Dapagliflozin Effect on Cardiovascular Events-Thrombolysis in Myocardial Infarction

CREDENCE: Canagliflozin and Renal Outcomes in Type 2 Diabetesand Nephropathy

REWIND: Dulaglutide and Cardiovascular Outcomes in Type 2 Diabetes

DUAL VIII: Durability of Insulin Degludec plus Liraglutide versus Insulin Glargine U100 as Initial Injectable Therapy in Type 2 Diabetes

CAROLINA: Cardiovascular Outcome Study of Linagliptin versus Glimepiride in Patients with Type 2 Diabetes

**Table S3. Association between incidence of neoplasm and weight reduction difference stratified by neoplasm sites**

| **Neoplasm site** | **Incidence of neoplasm** | | | | **Weight change** | | | |
| --- | --- | --- | --- | --- | --- | --- | --- | --- |
|  | **No. of participants** | **OR** | **95% CI** | ***P* value** | **No. of participants** | **WMD** | **95% CI** | ***P* value** |
| **Gastric neoplasm** | | | | | | | | |
| Total | 32737/27034 | 1.09 | 0.70 to 1.70 | 0.71 | 29196/27795 | -2.45 | -2.79 to -2.11 | <0.001 |
| GLP-1RA | 17397/16215 | 0.88 | 0.48 to 1.61 | 0.67 | 16996/17042 | -2.25 | -2.69 to -1.80 | <0.001 |
| DPP-4 inhibitor | 3023/3010 | 1.79 | 0.60 to 5.36 | 0.29 | 3023/3010 | NA | NA | NA |
| SGLT2 inhibitor | 12317/7809 | 1.20 | 0.52 to 2.79 | 0.67 | 12200/10753 | -2.53 | -3.04 to -2.03 | <0.001 |
| **Colon/rectal neoplasm** | | | | | | | | |
| Total | 35231/29378 | 1.18 | 0.94 to 1.48 | 0.15 | 30581/29090 | -2.26 | -2.54 to -1.98 | <0.001 |
| GLP-1RA | 17819/17252 | 1.27 | 0.93 to 1.72 | 0.13 | 17628/17466 | -2.28 | -2.72 to -1.84 | <0.001 |
| DPP-4 inhibitor | 3023/3010 | 1.06 | 0.64 to 1.75 | 0.81 | 3023/3010 | NA | NA | NA |
| SGLT2 inhibitor | 13592/8321 | 1.15 | 0.72 to 1.84 | 0.56 | 12865/11437 | -2.39 | -2.78 to -2.01 | <0.001 |
| Insulin | 797/795 | 0.60 | 0.08 to 4.54 | 0.62 | 720/611 | -1.36 | -2.05 to -0.68 | <0.001 |
| **Lung neoplasm** | | | | | | | | |
| Total | 32172/19575 | 0.83 | 0.63 to 1.09 | 0.18 | 25567/24029 | -2.63 | -2.99 to -2.77 | <0.001 |
| GLP-1RA | 9447/9343 | 0.87 | 0.58 to 1.31 | 0.51 | 9350/9355 | -2.92 | -4.96 to -0.88 | 0.005 |
| SGLT2 inhibitor | 22005/9623 | 0.82 | 0.56 to 1.22 | 0.33 | 15226/13786 | -2.62 | -2.94 to -2.30 | <0.001 |
| Insulin | 720/609 | 0.33 | 0.05 to 2.21 | 0.25 | 720/611 | -1.36 | -2.05 to -0.68 | <0.001 |
| **Pancreatic neoplasm** | | | | | | | | |
| Total | 37172/32464 | 1.02 | 0.75 to 1.38 | 0.92 | 29205/27803 | -2.43 | -2.71 to -2.15 | <0.001 |
| GLP-1RA | 21740/21677 | 1.25 | 0.82 to 1.90 | 0.29 | 27482/26100 | -2.71 | -2.54 to -1.80 | <0.001 |
| DPP-4 inhibitor | 3023/3010 | 0.66 | 0.35 to 1.25 | 0.20 | 3023/3010 | NA | NA | NA |
| SGLT2 inhibitor | 12409/7959 | 0.97 | 0.50 to 1.86 | 0.92 | 7562/6124 | -2.48 | -3.17 to -1.80 | <0.001 |
| **Skin neoplasm** | | | | | | | | |
| Total | 32437/26767 | 1.12 | 0.91 to 1.37 | 0.28 | 31612/30021 | -2.51 | -2.88 to -2.26 | <0.001 |
| GLP-1RA | 17423/16937 | 1.17 | 0.92 to 1.48 | 0.21 | 17832/17657 | -2.38 | -2.83 to -1.94 | <0.001 |
| SGLT2 inhibitor | 14508/9326 | 1.07 | 0.72 to 1.58 | 0.73 | 13780/12364 | -2.57 | -3.21 to -1.93 | <0.001 |
| Insulin | 506/504 | 0.14 | 0.01 to 2.75 | 0.20 | 506/506 | -1.70 | -1.74 to -1.66 | <0.001 |
| **Prostate neoplasm** | | | | | | | | |
| Total | 34317/26773 | 0.77 | 0.61 to 0.97 | **0.02** | 30197/28398 | -2.60 | -2.83 to -2.37 | <0.001 |
| GLP-1RA | 14998/14220 | 0.68 | 0.49 to 0.95 | **0.02** | 14594/14432 | -2.73 | -3.78 to -1.67 | <0.001 |
| DPP-4 inhibitor | 3023/3010 | 0.86 | 0.53 to 1.38 | 0.53 | 3023/3010 | NA | NA | NA |
| SGLT2 inhibitor | 16296/9543 | 0.86 | 0.56 to 1.33 | 0.51 | 15603/13966 | -2.60 | -2.84 to -2.36 | <0.001 |
| **Breast neoplasm** | | | | | | | | |
| Total | 45043/38169 | 1.10 | 0.86 to 1.40 | 0.44 | 41567/40759 | -2.38 | -2.57 to -2.18 | <0.001 |
| GLP-1RA | 14694/14220 | 1.12 | 0.74 to 1.71 | 0.59 | 14594/14432 | -2.73 | -3.78 to -1.67 | <0.001 |
| DPP-4 inhibitor | 3023/3010 | 1.05 | 0.55 to 2.01 | 0.88 | 3023/3010 | NA | NA | NA |
| SGLT2 inhibitor | 26529/20144 | 1.12 | 0.80 to 1.57 | 0.51 | 26253/25716 | -2.45 | -2.59 to -2.31 | <0.001 |
| Insulin | 797/795 | 0.60 | 0.08 to 4.54 | 0.62 | 720/611 | -1.36 | -2.05 to -0.68 | <0.001 |
| **Bladder neoplasm** | | | | | | | | |
| Total | 37374/32045 | 0.72 | 0.56 to 0.95 | **0.02** | 29232/28431 | -2.57 | -2.86 to -2.27 | <0.001 |
| GLP-1RA | 10672/10163 | 0.88 | 0.51 to 1.52 | 0.65 | 10511/10201 | -2.66 | -4.10 to -1.21 | <0.001 |
| DPP-4 inhibitor | 3023/3010 | 0.44 | 0.19 to 1.02 | 0.05 | 3023/3010 | NA | NA | NA |
| SGLT2 inhibitor | 23970/19163 | 0.75 | 0.54 to 1.04 | 0.08 | 18812/18230 | -2.59 | -2.86 to -2.33 | <0.001 |
| insulin | 291/291 | 3.01 | 0.12 to 74.20 | 0.50 | 214/105 | -1.00 | -1.24 to -0.76 | <0.001 |
| **Brain neoplasm** | | | | | | | | |
| Total | 29308/24416 | 1.70 | 0.88 to 3.30 | 0.11 | 29071/27656 | -2.29 | -2.62 to -1.97 | <0.001 |
| GLP-1RA | 16791/16725 | 2.62 | 0.98 to 6.98 | 0.05 | 16694/16727 | -2.49 | -3.00 to -1.99 | <0.001 |
| SGLT2 inhibitor | 12517/7691 | 1.15 | 0.46 to 2.82 | 0.77 | 12377/10929 | -2.25 | -2.54 to -1.97 | <0.001 |
| **Hematologic neoplasm** | | | | | | | | |
| Total | 29630/24408 | 0.76 | 0.55 to 1.05 | 0.10 | 29206/27618 | -2.56 | -2.85 to -2.27 | <0.001 |
| GLP-1RA | 16791/16725 | 0.76 | 0.51 to 1.14 | 0.19 | 16694/16727 | -2.49 | -3.00 to -1.99 | <0.001 |
| SGLT2 inhibitor | 12839/7683 | 0.75 | 0.43 to 1.32 | 0.32 | 12512/10891 | -2.54 | -2.99 to -2.09 | <0.001 |
| **Thyroid neoplasm** | | | | | | | | |
| Total | 37574/33017 | 1.50 | 0.92 to 2.42 | 0.10 | 34029/32648 | -2.58 | -2.95 to -2.21 | <0.001 |
| GLP-1RA | 22648/22299 | 2.00 | 1.10 to 3.63 | **0.02** | 22260/22311 | -2.13 | -2.42 to -1.84 | <0.001 |
| DPP-4 inhibitor | 3023/3010 | 0.33 | 0.03 to 3.19 | 0.34 | 3023/3010 | NA | NA | NA |
| SGLT2 inhibitor | 11903/7708 | 0.97 | 0.37 to 2.50 | 0.94 | 11769/10337 | -2.79 | -4.10 to -1.48 | <0.001 |
| **Ovarian neoplasm** | | | | | | | | |
| Total | 23638/21840 | 1.30 | 0.59 to 2.88 | 0.52 | 23109/21730 | -2.25 | -2.66 to -1.84 | <0.001 |
| GLP-1RA | 16791/16725 | 2.18 | 0.76 to 6.29 | 0.15 | 16694/16727 | -2.49 | -3.00 to -1.99 | <0.001 |
| SGLT2 inhibitor | 6341/4611 | 0.44 | 0.10 to 2.05 | 0.30 | 5909/4497 | -2.00 | -2.00 to -2.00 | <0.001 |
| Insulin | 506/504 | 1.00 | 0.06 to 15.97 | 1.00 | 506/506 | -1.70 | -1.74 to -1.66 | <0.001 |
| **Renal neoplasm** | | | | | | | | |
| Total | 31843/27011 | 1.22 | 0.82 to 1.81 | 0.32 | 31595/30195 | -2.73 | -3.09 to -2.37 | <0.001 |
| GLP-1RA | 16791/16725 | 1.22 | 0.73 to 2.03 | 0.44 | 16694/16727 | -2.49 | -3.00 to -1.99 | <0.001 |
| SGLT2 inhibitor | 15052/10286 | 1.22 | 0.65 to 2.29 | 0.54 | 14901/13468 | -2.72 | -3.66 to -1.79 | <0.001 |
| **Hepatic neoplasm** | | | | | | | | |
| Total | 29428/24524 | 0.98 | 0.64 to 1.51 | 0.94 | 29182/27551 | -2.29 | -2.63 to -1.95 | <0.001 |
| GLP-1RA | 17423/16937 | 1.00 | 0.57 to 1.75 | 1.00 | 17326/17151 | -2.53 | -3.03 to -2.03 | <0.001 |
| SGLT2 inhibitor | 11499/7083 | 1.02 | 0.51 to 2.04 | 0.96 | 11350/9894 | -2.11 | -2.80 to -1.42 | <0.001 |
| Insulin | 506/504 | 0.33 | 0.01 to 8.15 | 0.50 | 506/506 | -1.70 | -1.74 to -1.66 | <0.01 |
| **Gallbladder/Bile duct neoplasm** | | | | | | | | |
| Total | 28437/23878 | 1.06 | 0.57 to 1.98 | 0.85 | 28241/26765 | -1.89 | -2.22 to -1.57 | <0.001 |
| GLP-1RA | 16791/16725 | 1.27 | 0.57 to 2.84 | 0.56 | 16694/16727 | -2.49 | -3.00 to -1.99 | <0.001 |
| SGLT2 inhibitor | 11646/7153 | 0.81 | 0.31 to 2.16 | 0.67 | 11547/10038 | -1.74 | -1.88 to -1.59 | <0.001 |
| **Uterine neoplasm** | | | | | | | | |
| Total | 31620/26386 | 1.41 | 0.72 to 1.79 | 0.58 | 31055/29507 | -2.72 | -3.08 to -2.36 | <0.001 |
| GLP-1RA | 17095/17040 | 1.31 | 0.68 to 2.50 | 0.42 | 16996/17042 | -2.25 | -2.69 to -1.80 | <0.001 |
| SGLT2 inhibitor | 14019/8842 | 1.05 | 0.55 to 2.02 | 0.88 | 13553/11959 | -2.87 | -3.83 to -1.91 | <0.001 |
| Insulin | 506/504 | 0.33 | 0.01 to 8.15 | 0.50 | 302/315 | -1.13 | -1.17 to -1.09 | <0.001 |
| **Head and neck neoplasm** | | | | | | | | |
| Total | 17929/16517 | 1.23 | 0.70 to 2.16 | 0.47 | 20690/19309 | -1.73 | -2.34 to -1.13 | <0.001 |
| GLP-1RA | 15043/15076 | 1.16 | 0.63 to 2.12 | 0.64 | 15046/15078 | -1.33 | -1.70 to -0.97 | <0.001 |
| SGLT2 inhibitor | 2886/1441 | 1.75 | 0.36 to 8.43 | 0.49 | 5644/4231 | -2.00 | -2.00 to -2.00 | <0.001 |

GLP-1RA, glucagon-like peptide-1 receptor agonist; DPP-4, dipeptidyl-peptidase-4; SGLT2, sodium glucose co-transporter 2; NA, not available. Font bold was used if *P*<0.05 was noticed in incidence of neoplasm.

**Figure S1. Funnel plot figure of publication bias for randomized clinical trials**

**
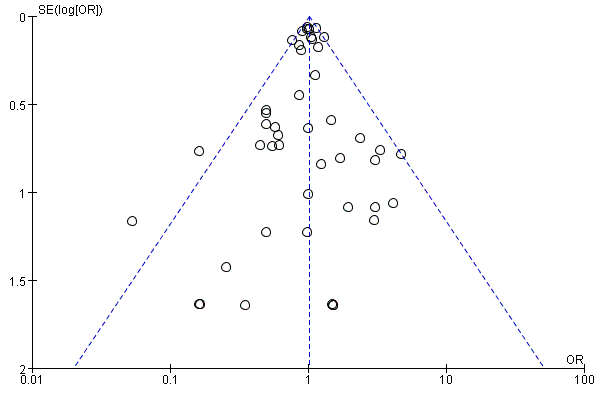
**

1. Trials with weight reduction difference

**
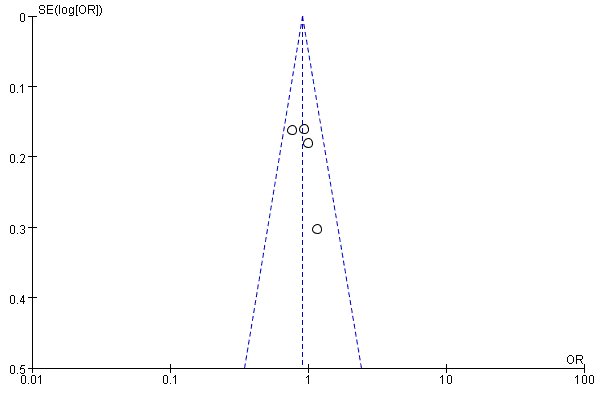
**

1. Trials with weight elevation change

**Figure S2. The association between weight reduction difference and the incidence of neoplasm stratified by drug types**


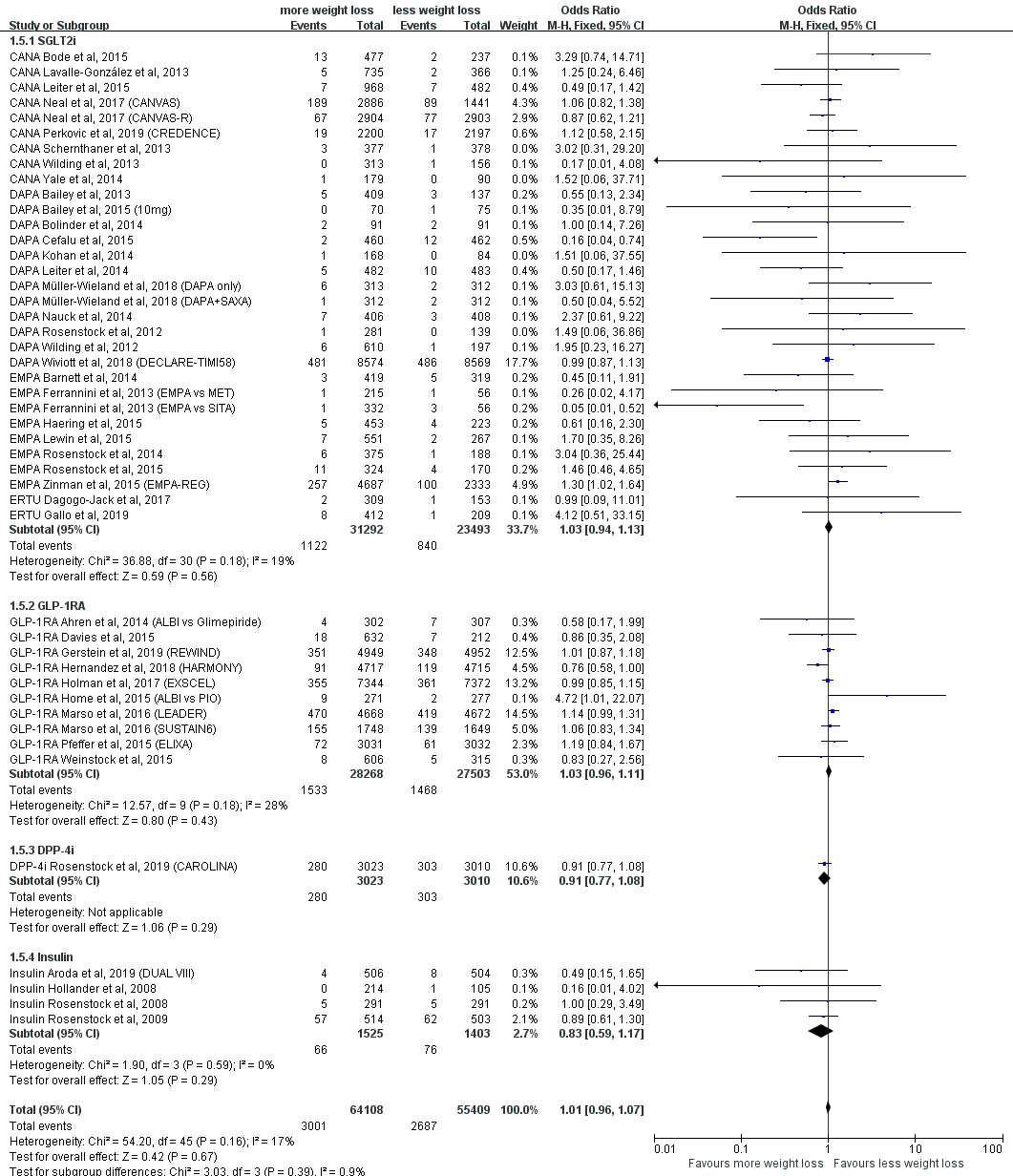


**Figure S3. The association between weight reduction difference and the incidence of neoplasm stratified by study types**


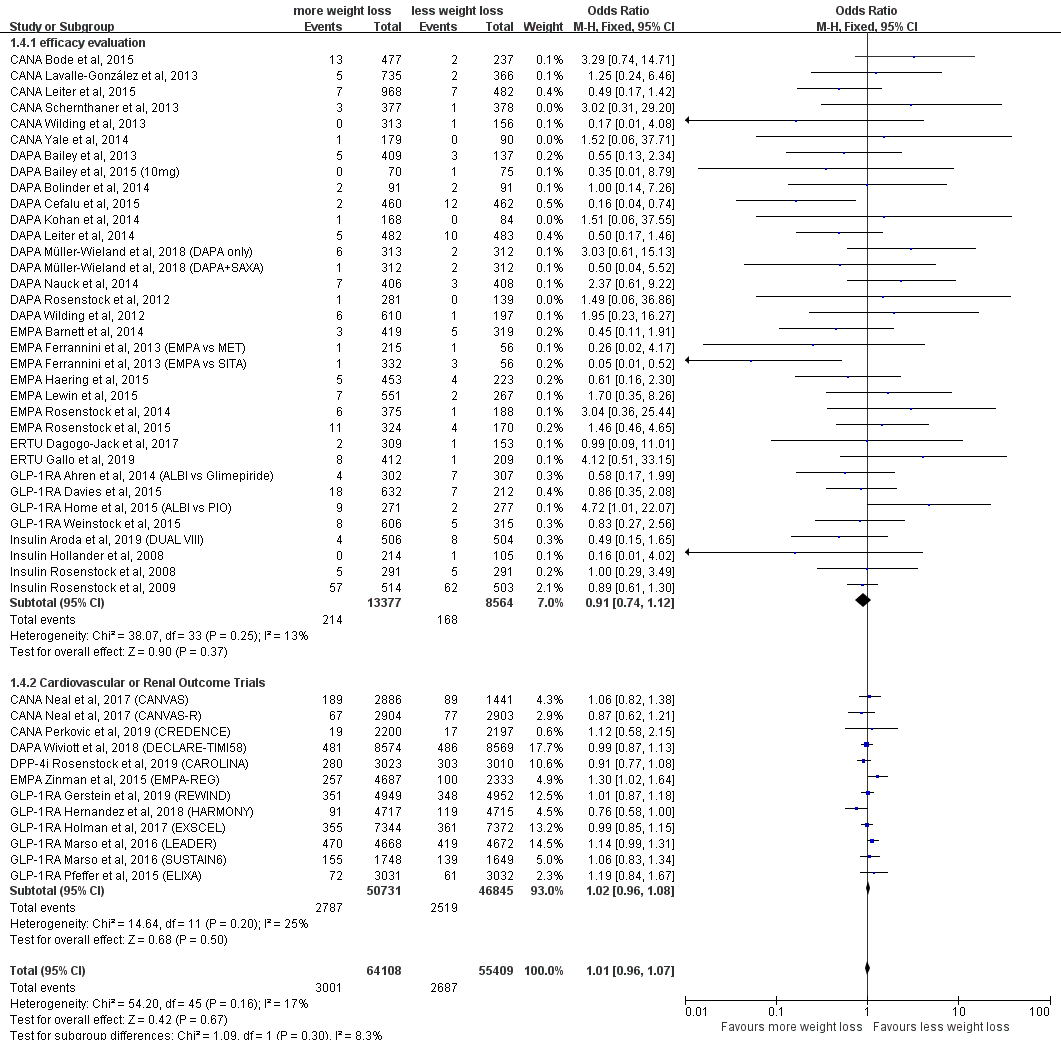


**Figure S4. The association between weight reduction difference and the incidence of neoplasm stratified by source of neoplasm events**


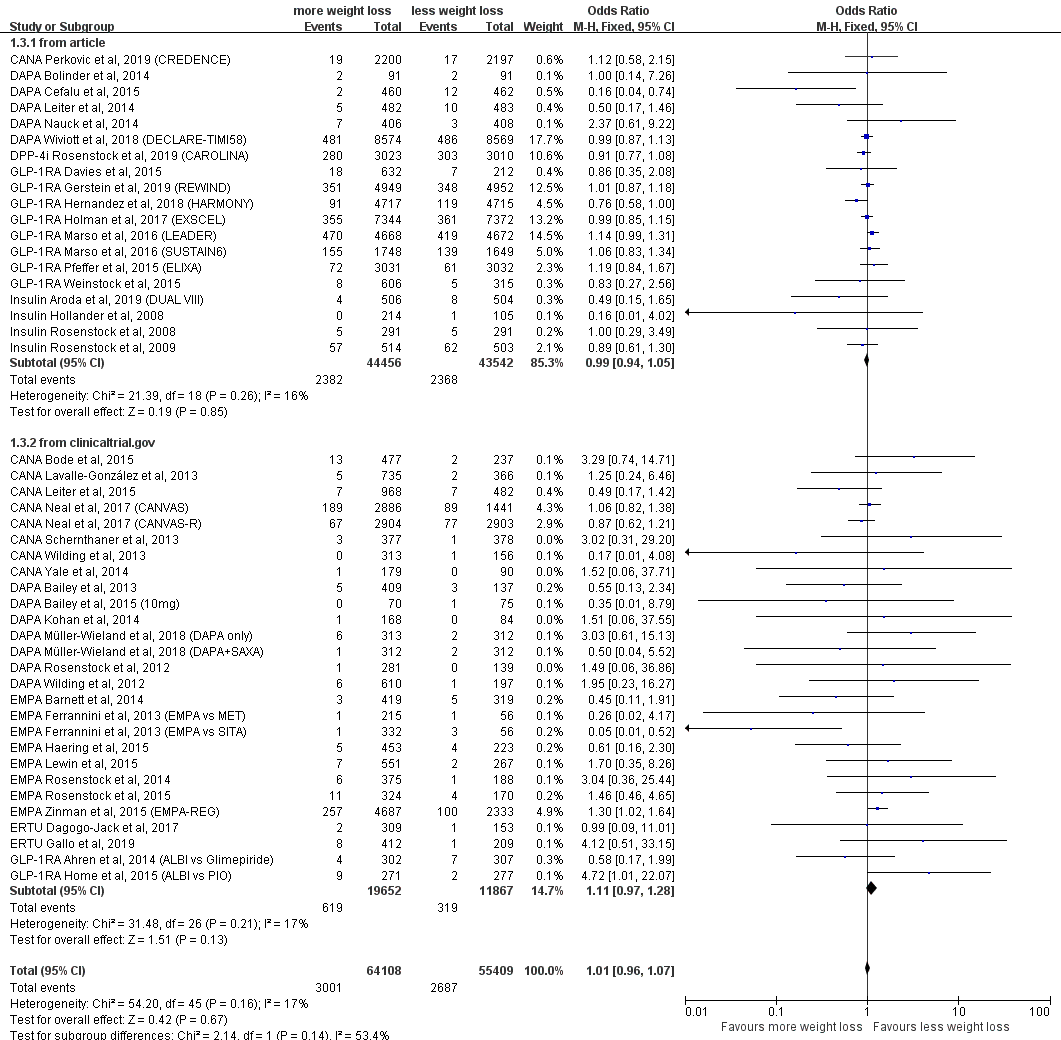


**Figure S5. The association between weight reduction difference and the incidence of neoplasm stratified by patient body mass index**


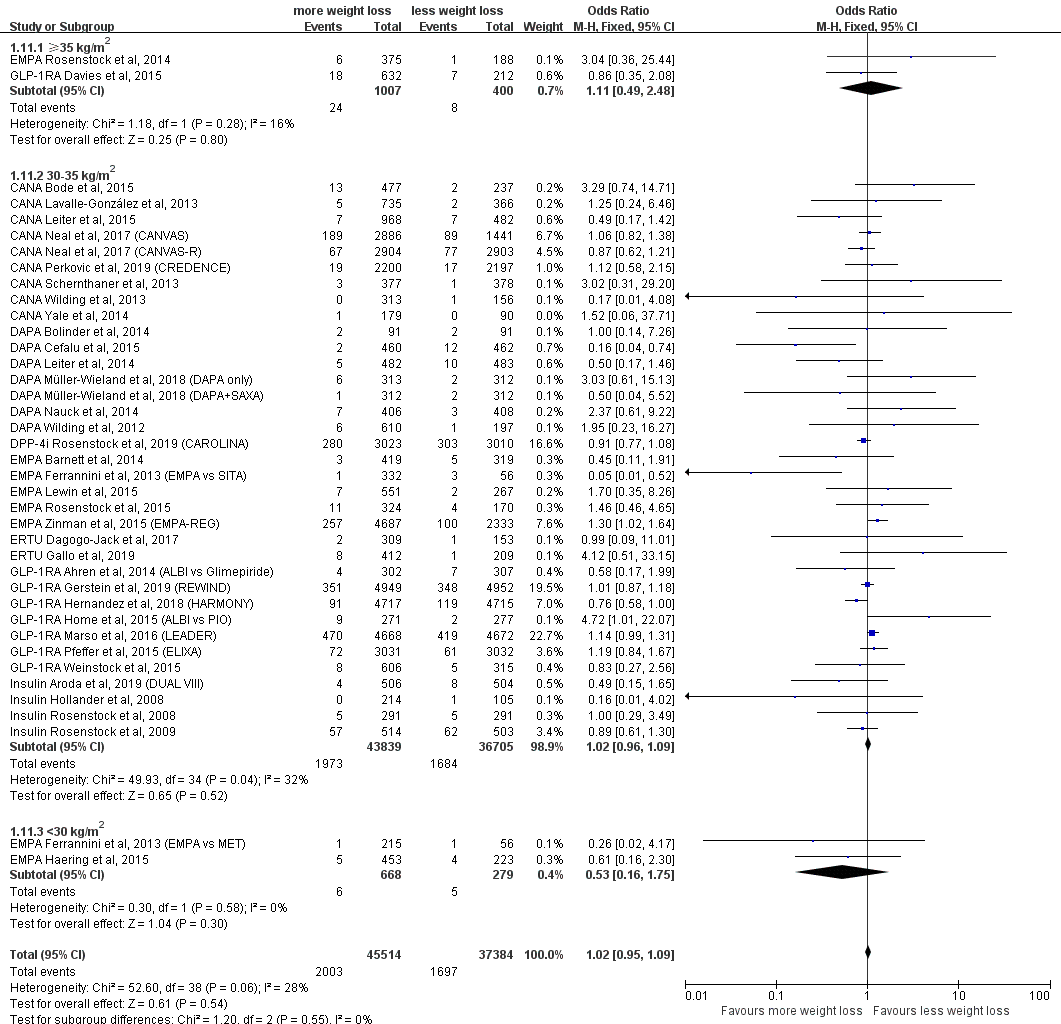


**Figure S6. The association between weight reduction difference and the incidence of neoplasm stratified by treatment design**

**
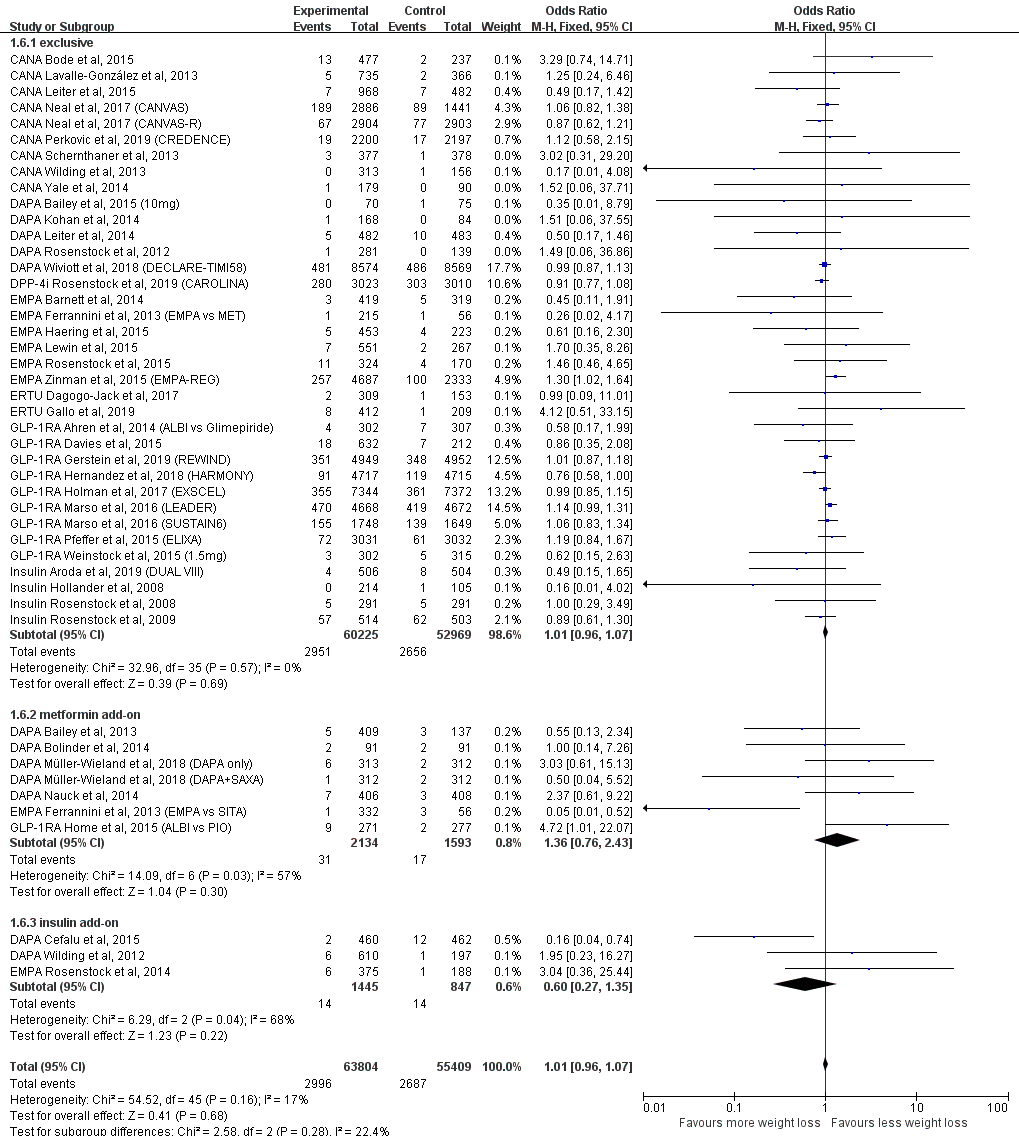
**

**Figure S7. The association between weight reduction difference and the incidence of neoplasm stratified by control agents**


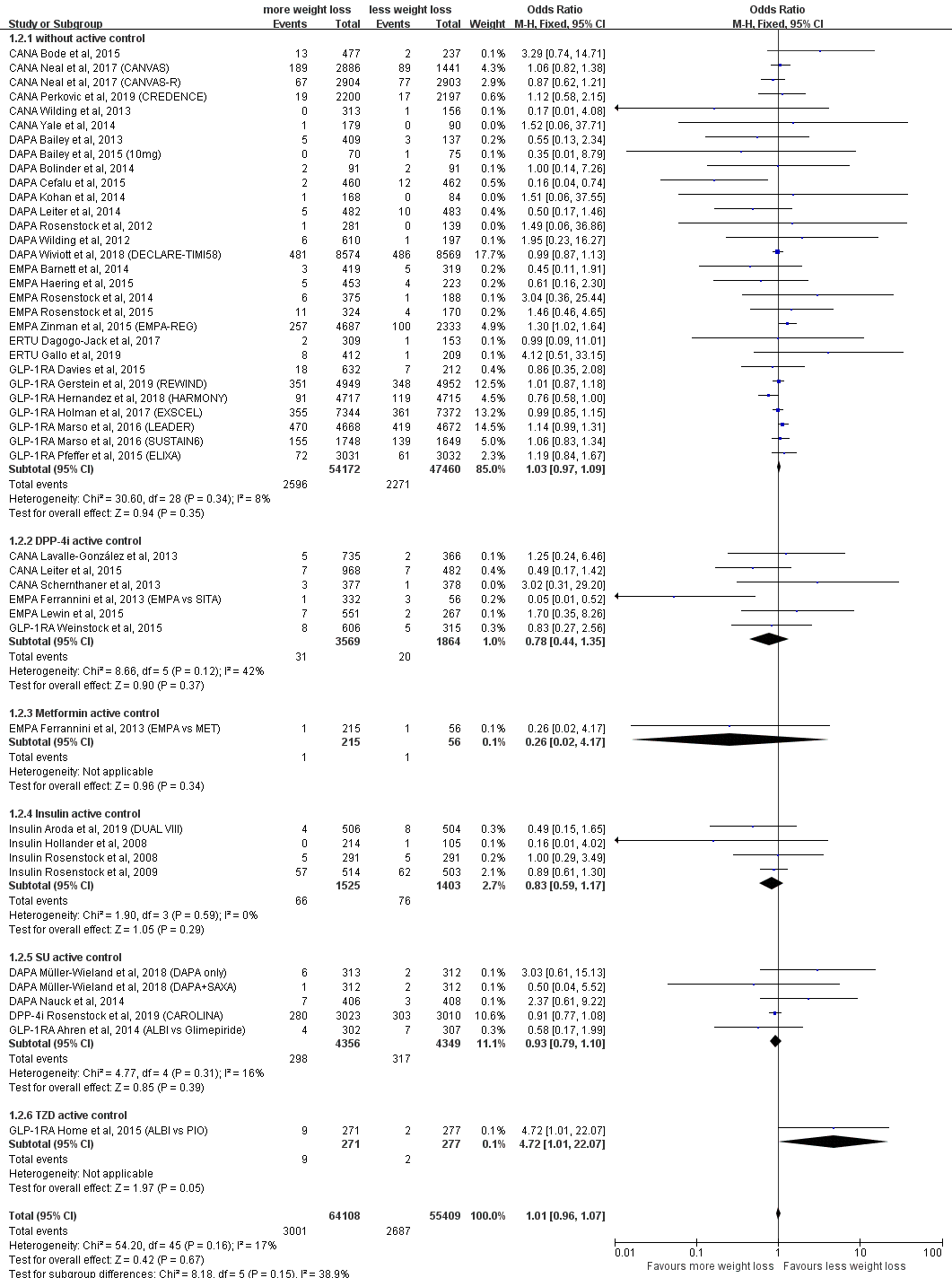


**Figure S8. The association between weight reduction difference and the incidence of neoplasm stratified by patient age**


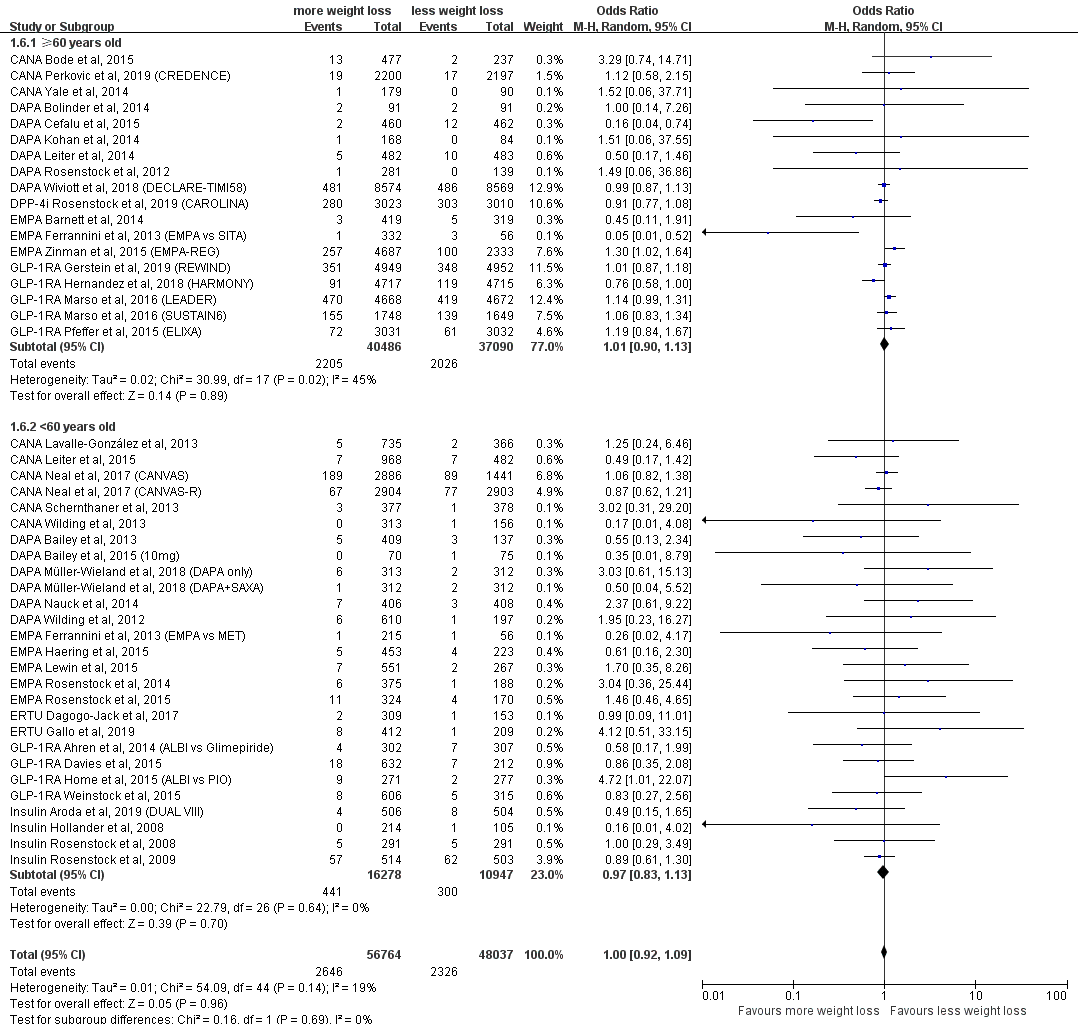


**Figure S9. The association between weight reduction difference and the incidence of neoplasm stratified by male percentage**


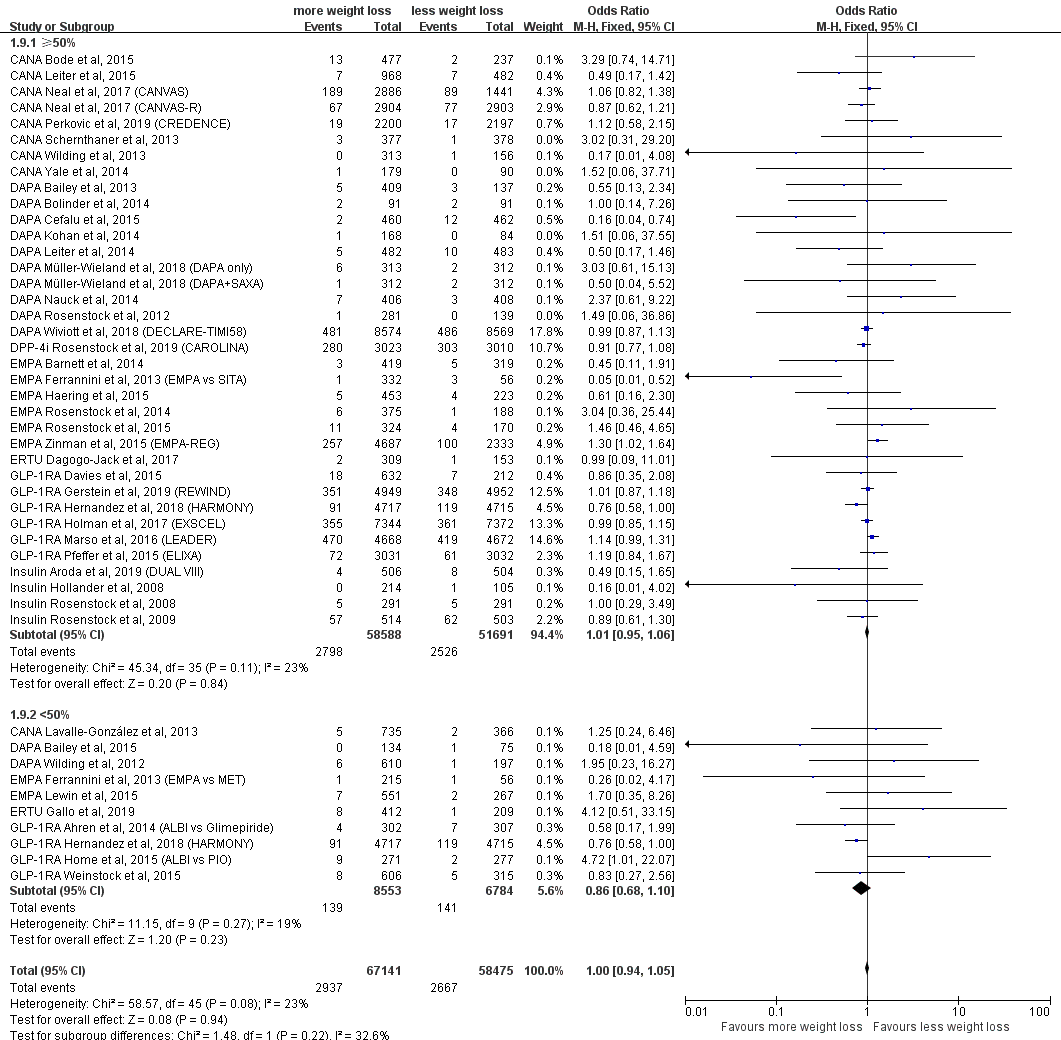


**Figure S10. The association between weight reduction difference and the incidence of neoplasm stratified by follow-up duration**

**
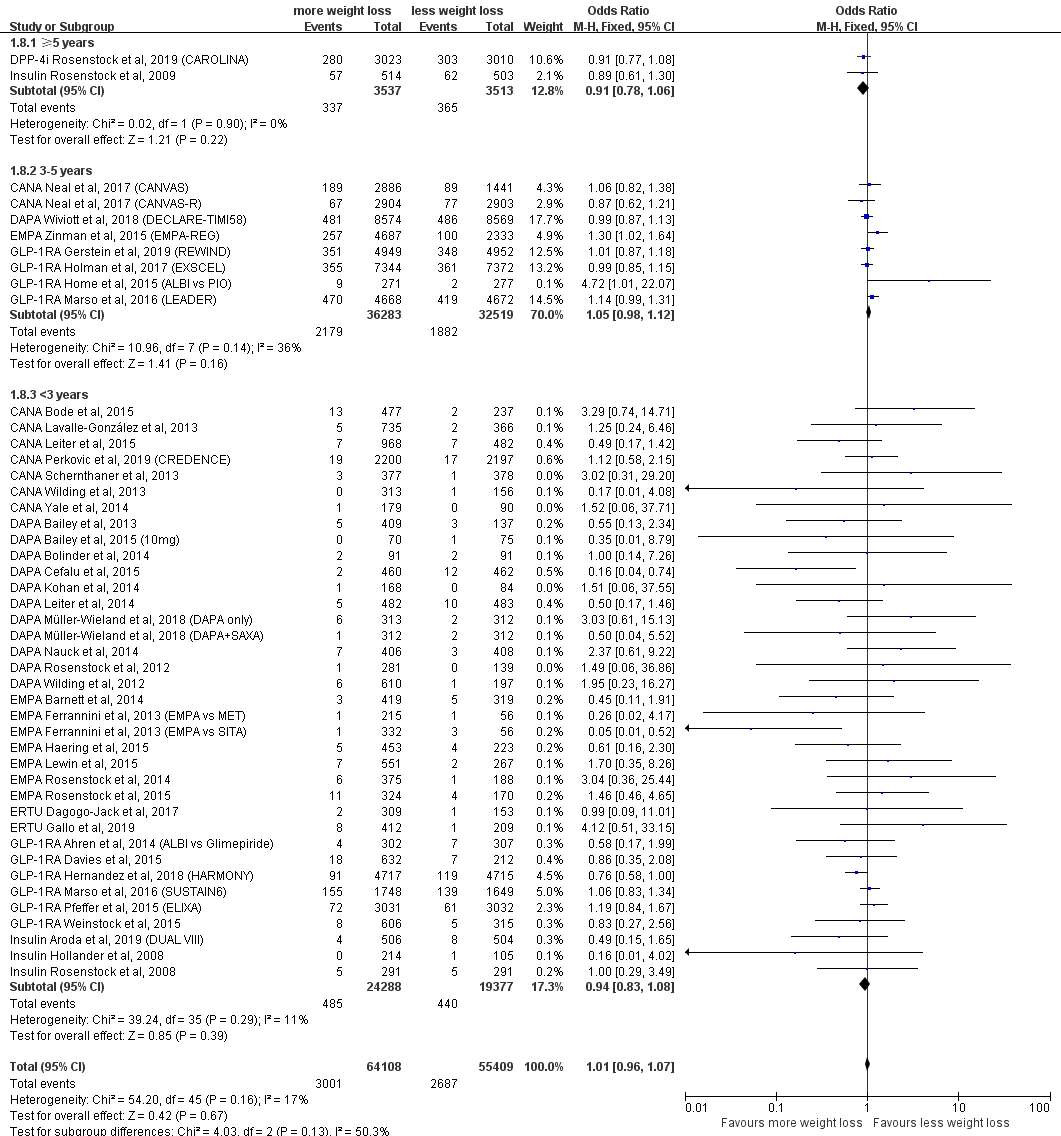
**

**Figure S11. The association between weight reduction difference and the incidence of neoplasm stratified by duration of diabetes**


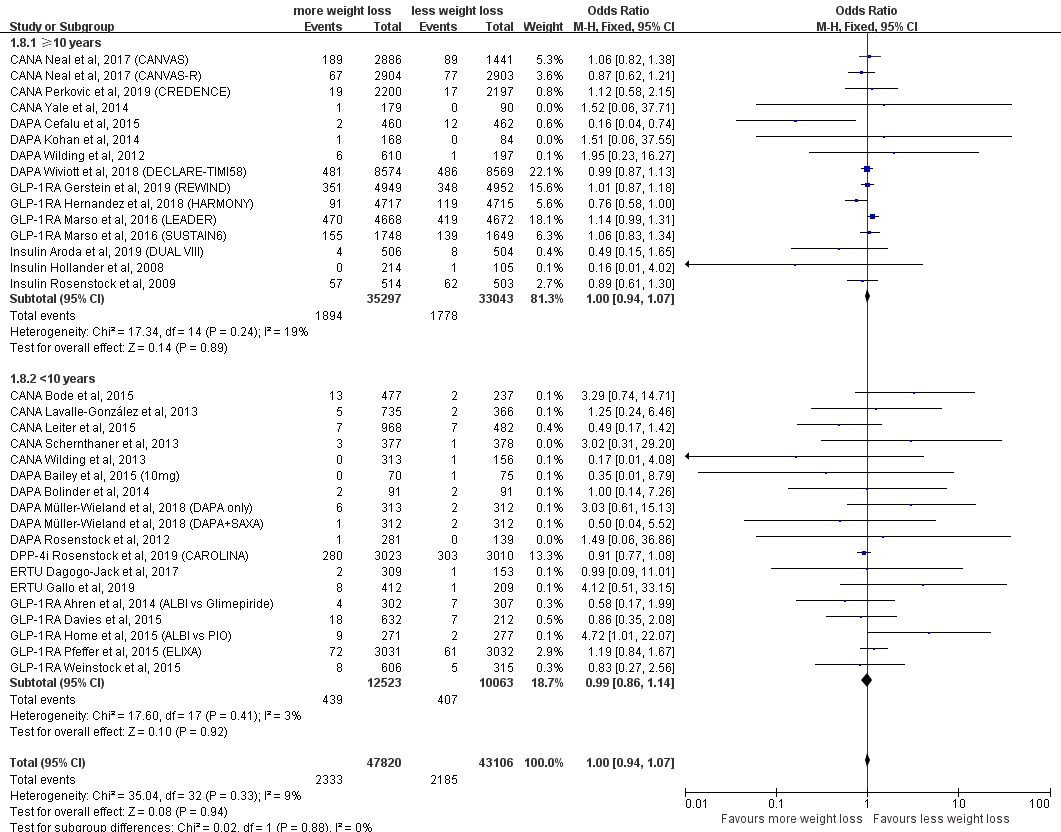


**Figure S12. The association between weight reduction difference or change rate and the incidence of gastric neoplasm**


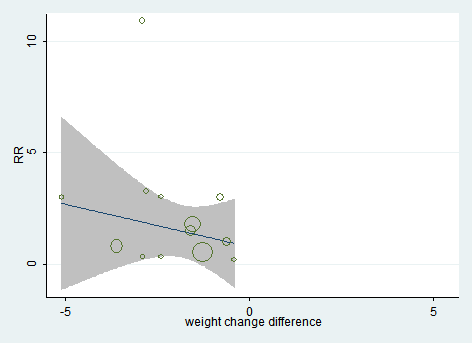


1. Weight reduction difference and incidence of gastric neoplasm (β=-0.5579, 95% CI, -1.9014 to 0.7856, *P*=0.380)


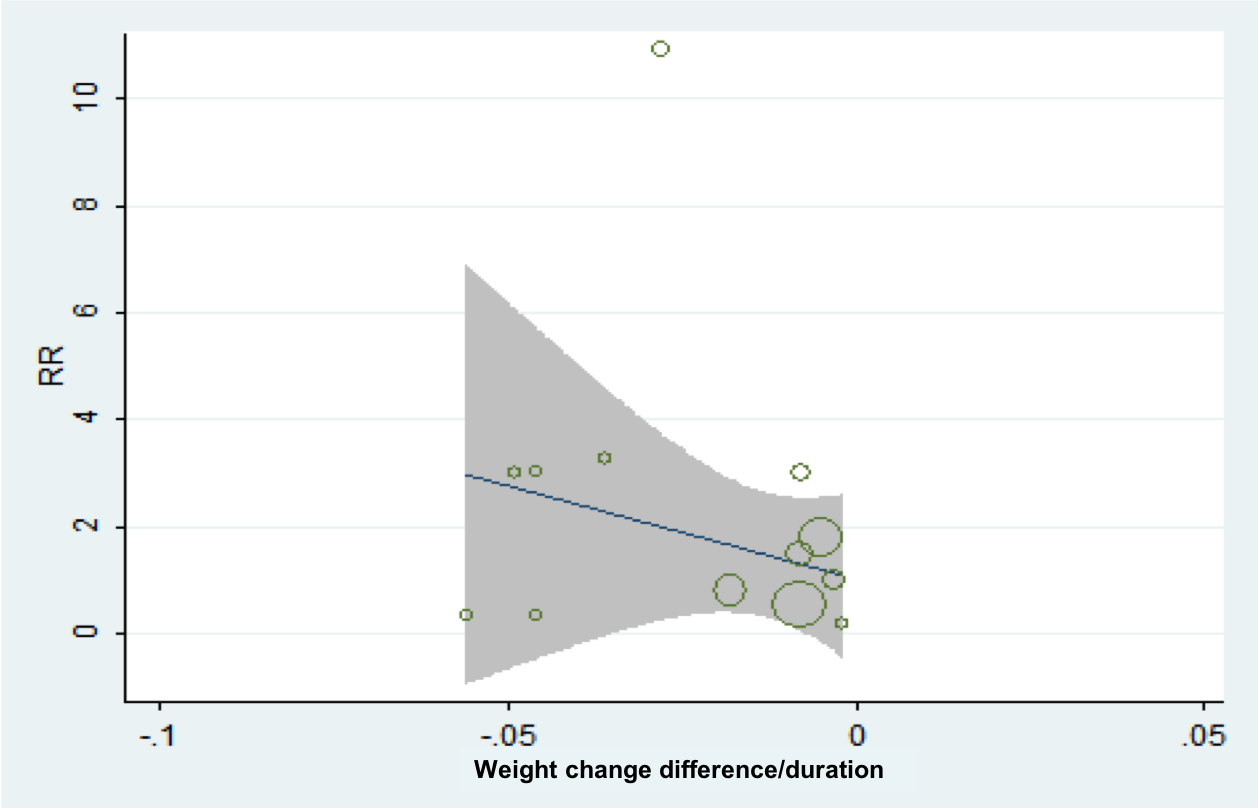


1. Weight reduction rate and incidence of gastric neoplasm (β=-23.0852, 95% CI, -114.5754 to 68.4049, *P*=0.590)

**Figure S13. The association between weight reduction difference or change rate and the incidence of colon/rectal neoplasm**


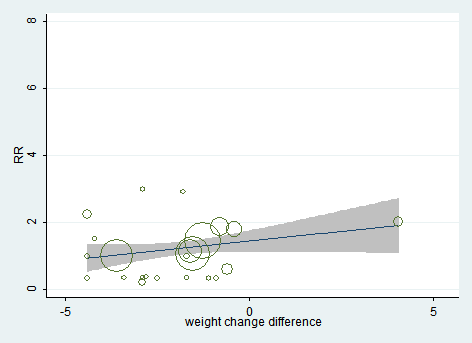


1. Weight reduction difference and incidence of colon/rectal neoplasm (β=0.1149, 95% CI, -0.0654 to 0.2951, *P*=0.199)


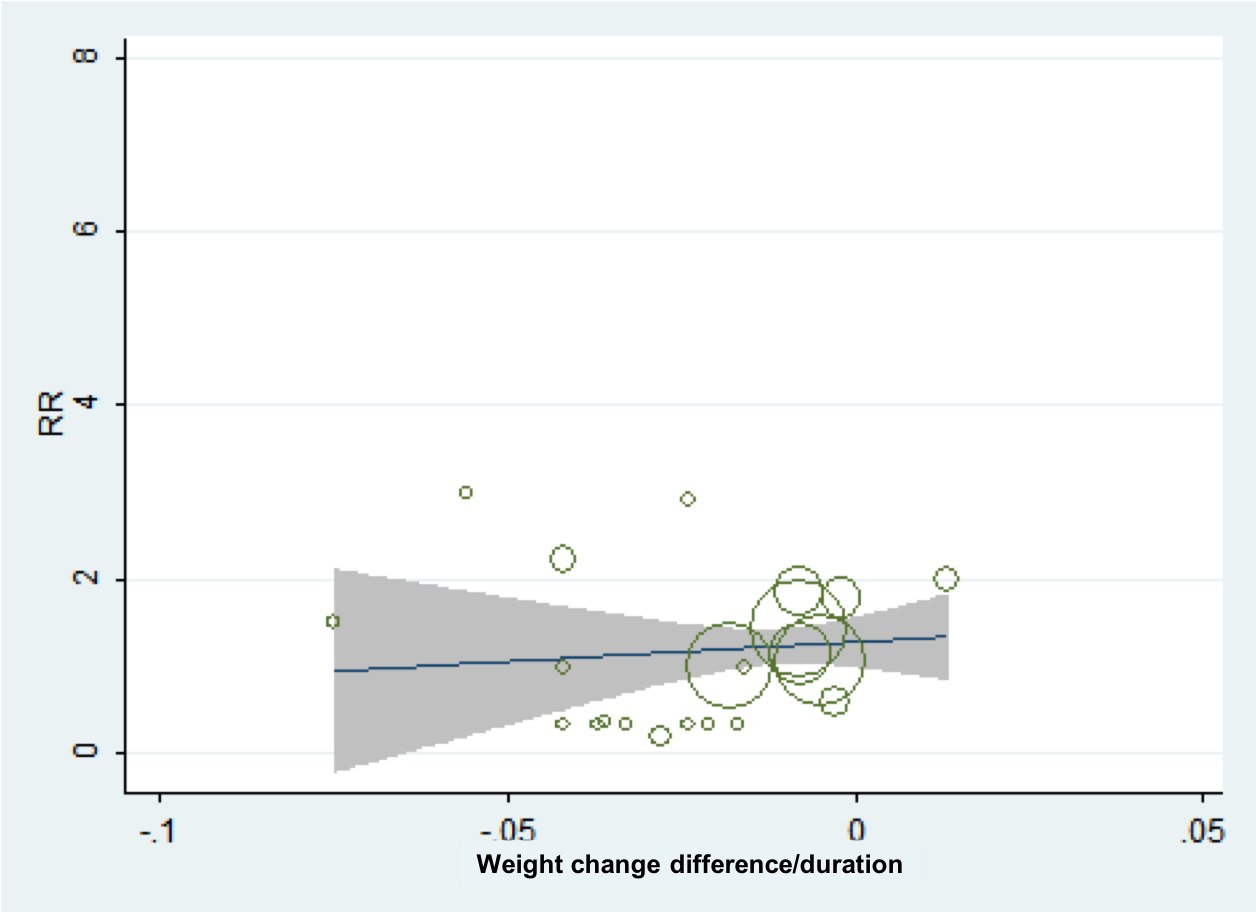


1. Weight reduction rate and incidence of colon/rectal neoplasm (β=4.4595, 95% CI, -18.1271 to 27.0462, *P*=0.686)

**Figure S14. The association between weight reduction difference or change rate and the incidence of lung neoplasm**


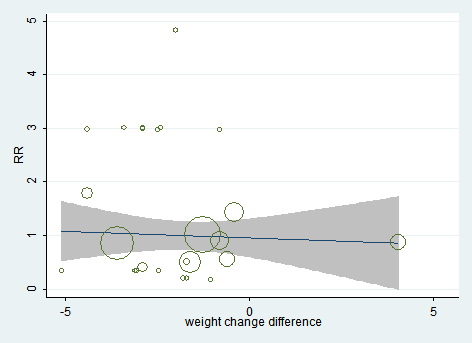


1. Weight reduction difference and incidence of lung neoplasm (β=-0.0243, 95% CI, -0.1680 to 0.1194, *P*=0.730)


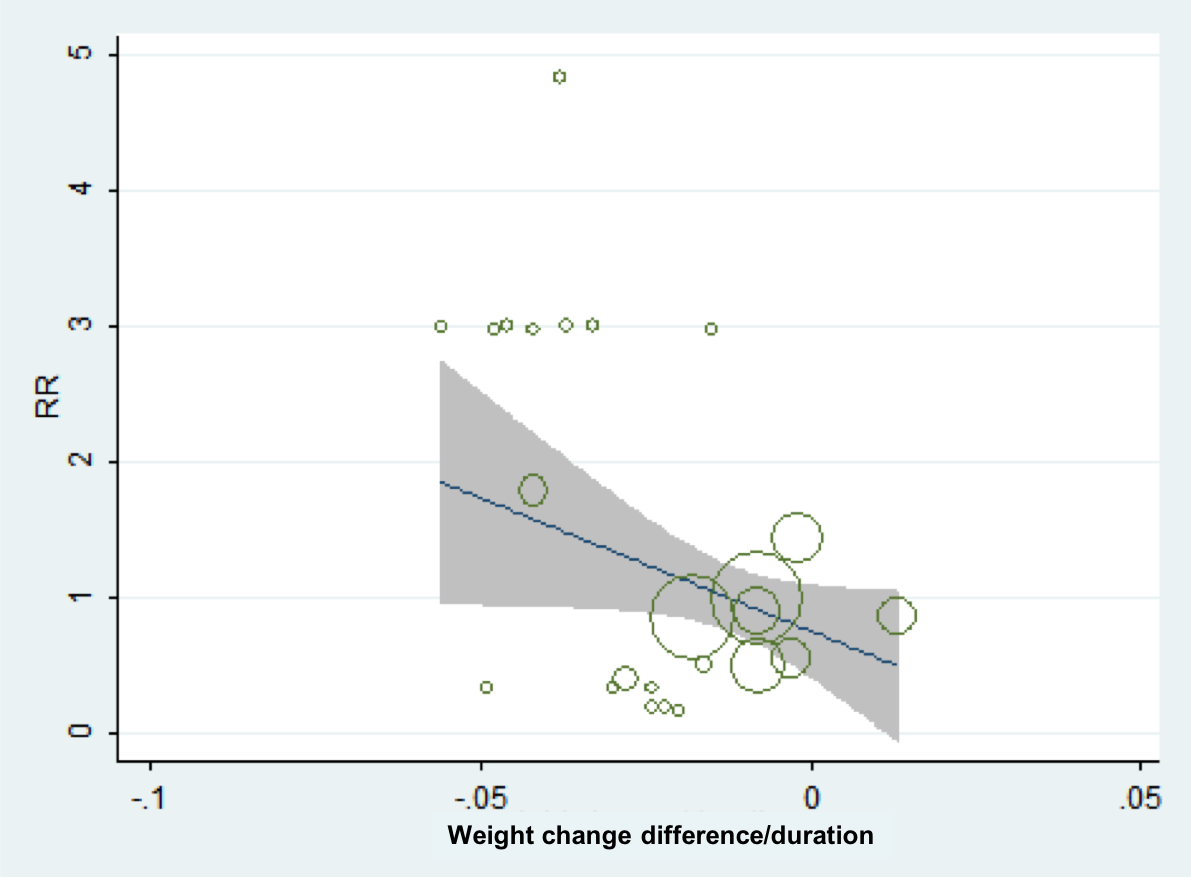


1. Weight reduction rate and incidence of lung neoplasm (β=-19.5552, 95% CI, -40.6248 to 1.5145, *P*=0.067)

**Figure S15. The association between weight reduction difference or change rate and the incidence of pancreatic neoplasm**


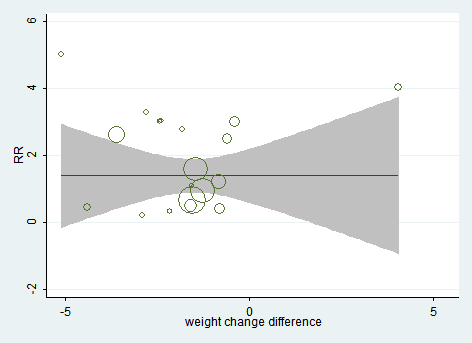


1. Weight reduction difference and incidence of pancreatic neoplasm (β=0.0776, 95% CI, -0.2979 to 0.4531, *P*=0.668)


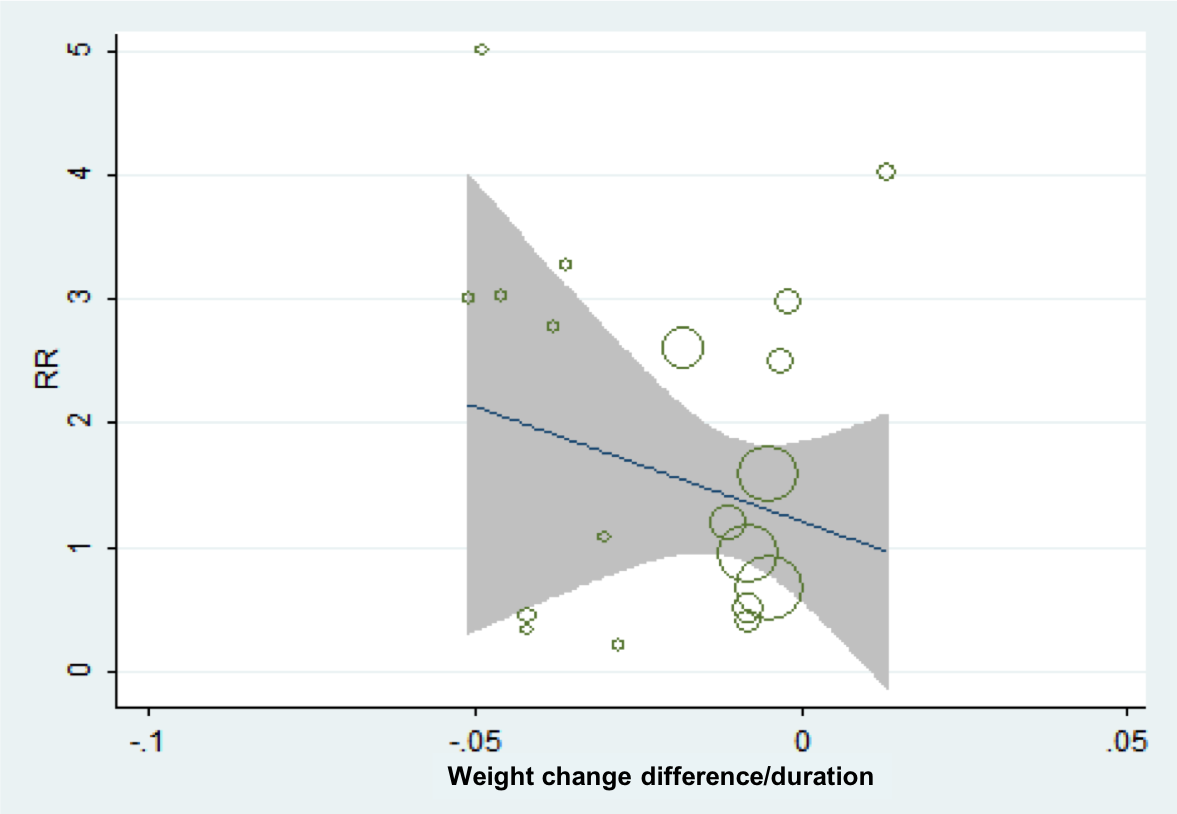


1. Weight reduction rate and incidence of pancreatic neoplasm (β=-10.2144, 95% CI, -49.0161 to 28.5872, *P*=0.586)

**Figure S16. The association between weight reduction difference or change rate and the incidence of skin neoplasm**


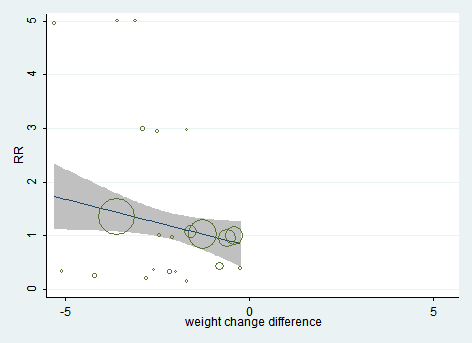


1. Weight reduction difference and incidence of skin neoplasm (β=-0.1742, 95% CI, -0.3508 to 0.0025, *P*=0.053)


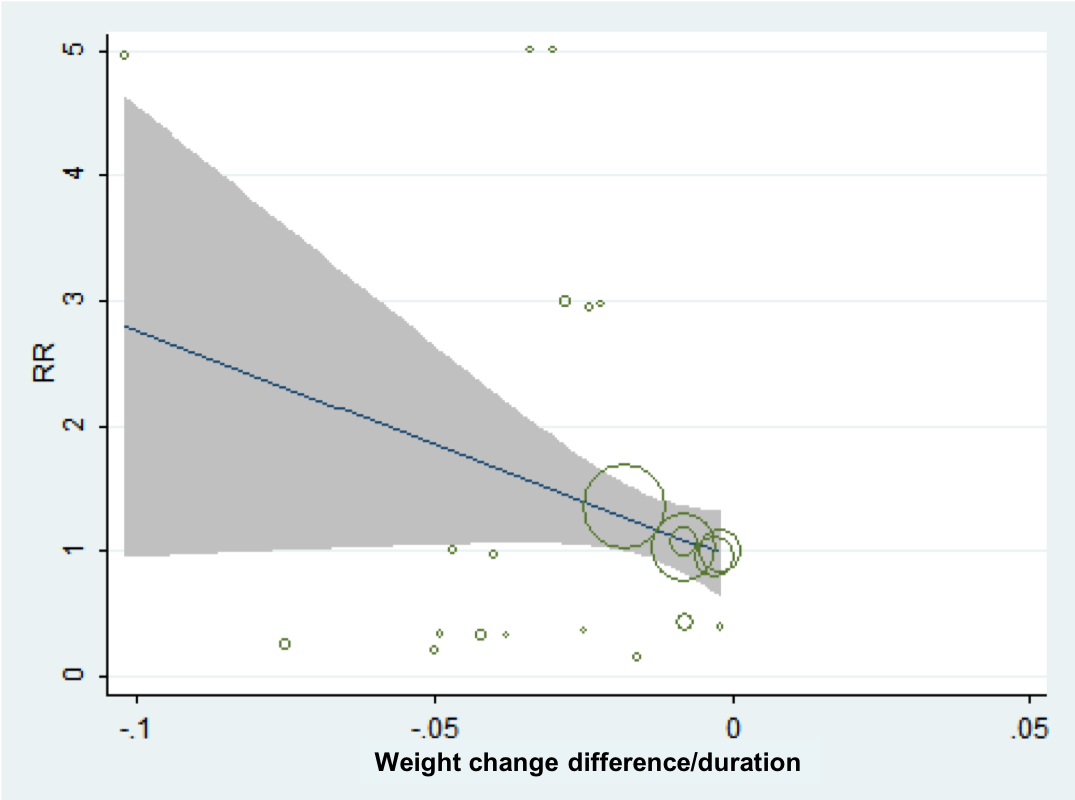


1. Weight reduction rate and incidence of skin neoplasm (β=-18.1167, 95% CI, -38.5991 to 2.3657, *P*=0.080)

**Figure S17. The association between weight reduction difference or change rate and the incidence of prostate neoplasm**


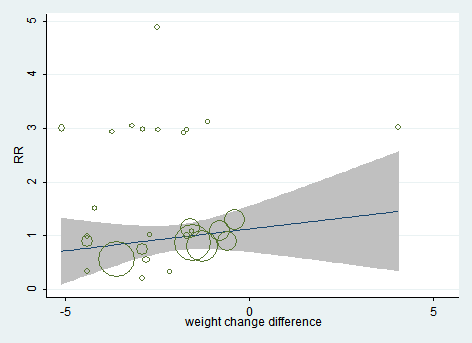


1. Weight reduction difference and incidence of prostate neoplasm (β=-0.0811, 95% CI, -0.0992 to 0.2612, *P*=0.364)


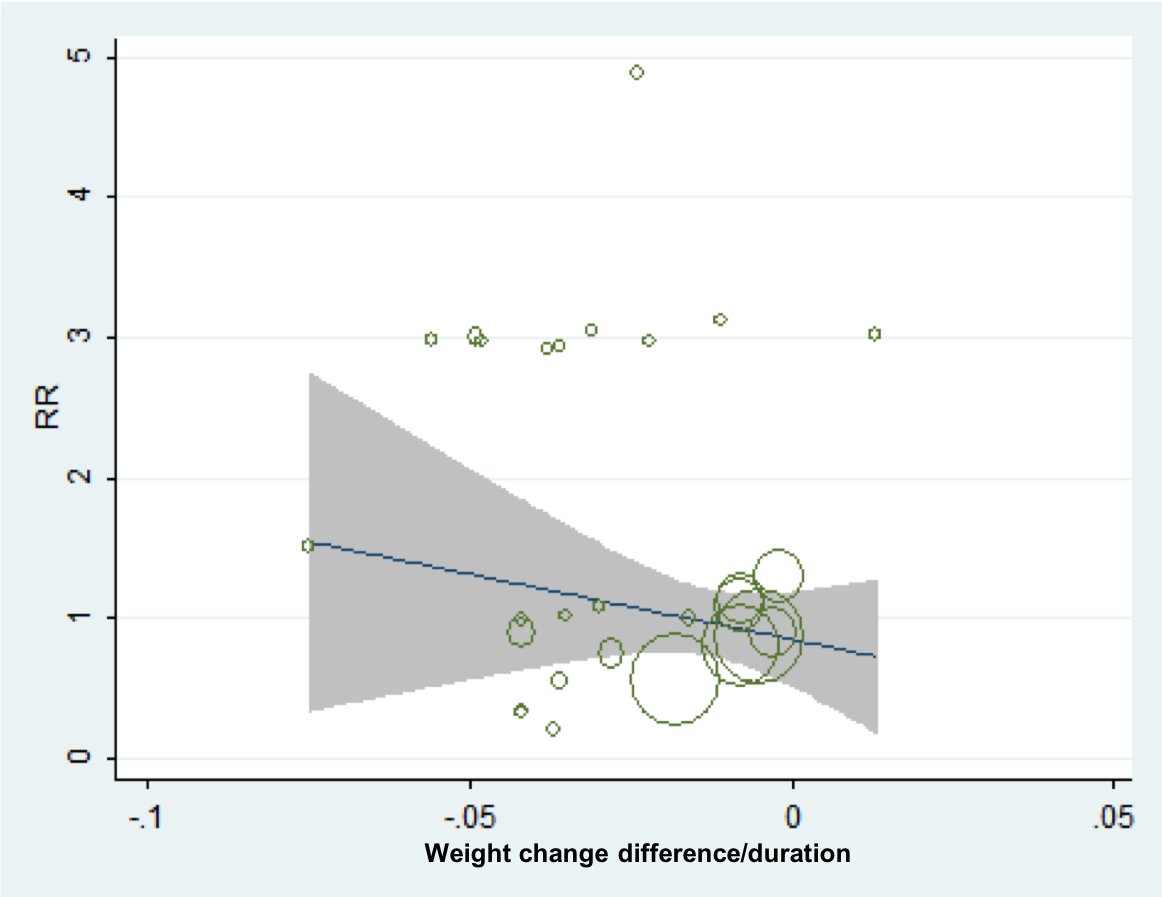


1. Weight reduction rate and incidence of prostate neoplasm (β=-10.2791, 95% CI, -29.9145 to 9.3564, *P*=0.292)

**Figure S18. The association between weight reduction difference or change rate and the incidence of breast neoplasm**


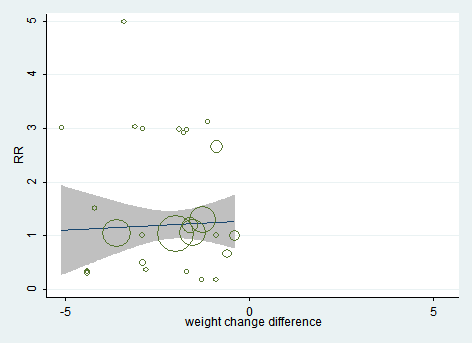


1. Weight reduction difference and incidence of breast neoplasm (β=0.0345, 95% CI, -0.2333 to 0.3023, *P*=0.793)


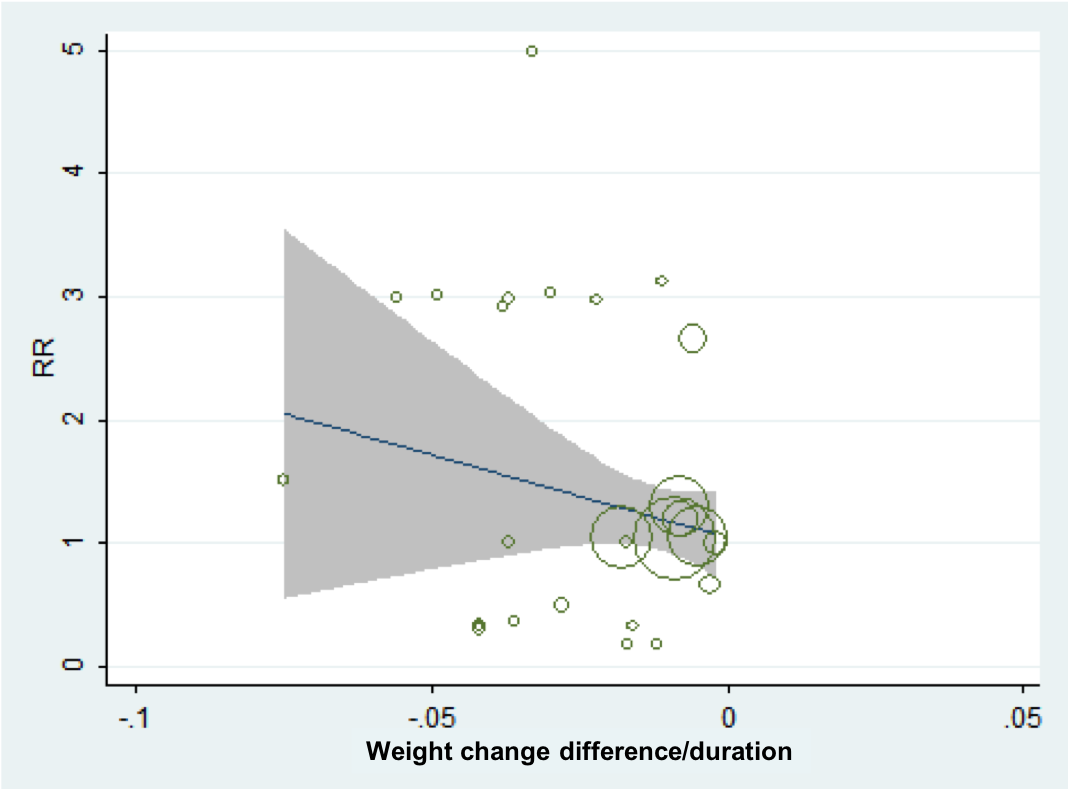


1. Weight reduction rate and incidence of breast neoplasm (β=-13.4092, 95% CI, -38.3263 to 11.5078, *P*=0.278)

**Figure S19. The association between weight reduction difference or change rate and the incidence of bladder neoplasm**


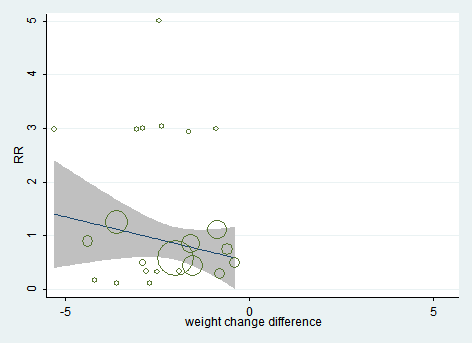


1. Weight reduction difference and incidence of bladder neoplasm (β=-0.1657, 95% CI, -0.4563 to 0.1248, *P*=0.380)


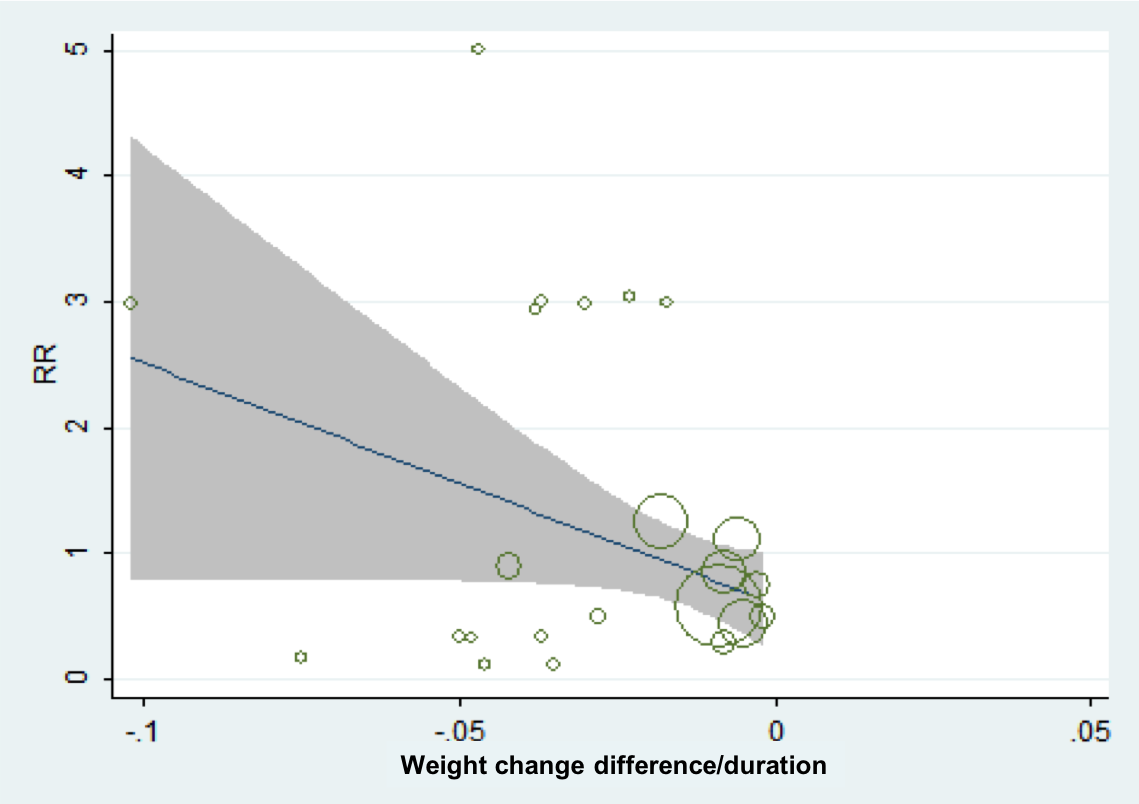


1. Weight reduction rate and incidence of bladder neoplasm (β=-19.0844, 95% CI, -39.6876 to 1.51882, *P*=0.068)

**Figure S20. The association between weight reduction difference or change rate and the incidence of brain neoplasm**


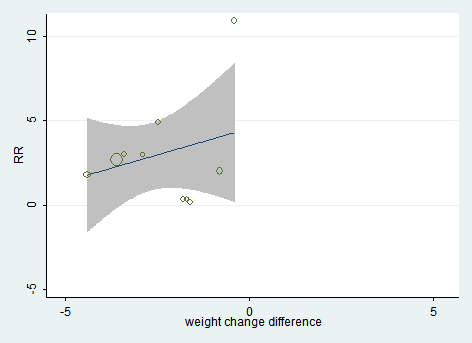


1. Weight reduction difference and incidence of brain neoplasm (β=-0.7577, 95% CI, -1.1206 to 2.6359, *P*=0.379)


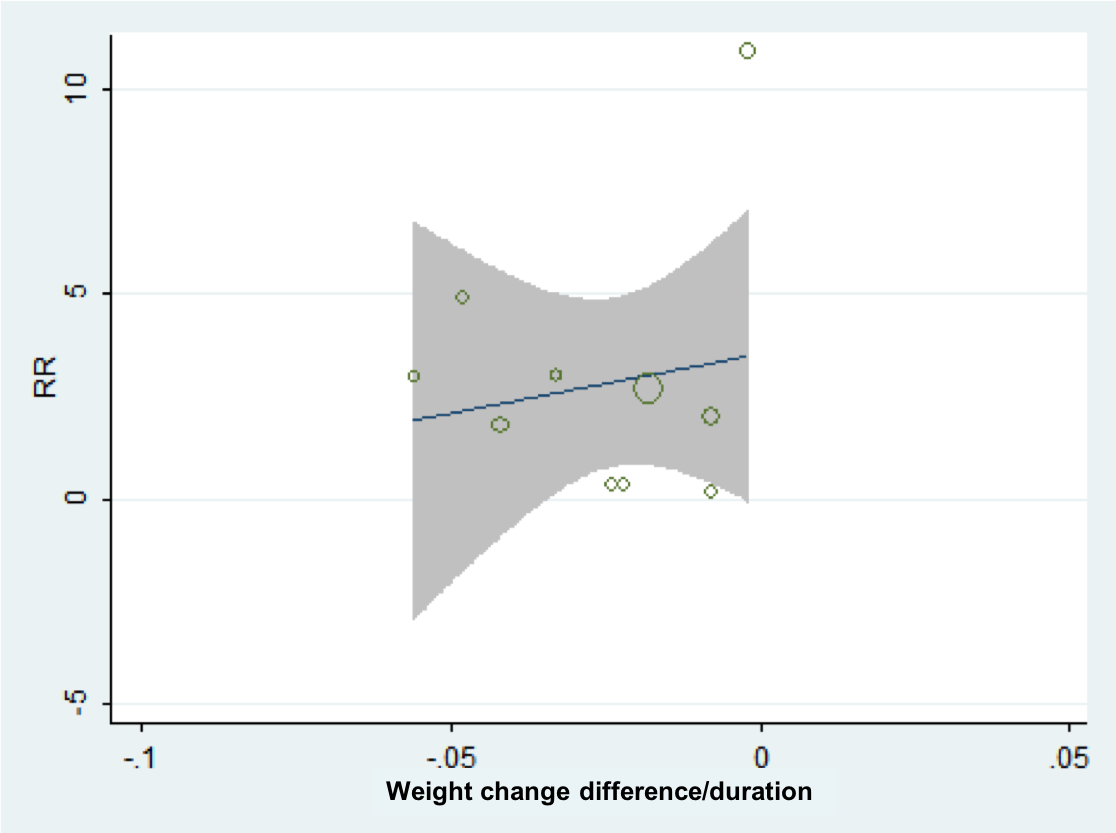


1. Weight reduction rate and incidence of brain neoplasm (β=27.9439, 95% CI, -112.1316 to 168.0193, *P*=0.658)

**Figure S21. The association between weight reduction difference or change rate and the incidence of hematologic neoplasm**


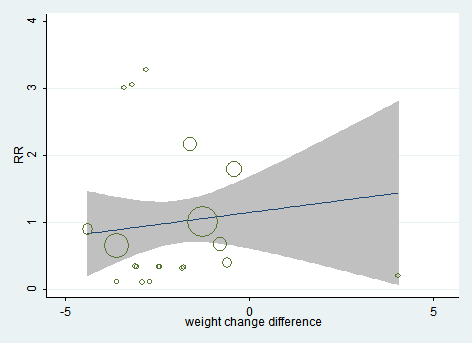


1. Weight reduction difference and incidence of hematologic neoplasm (β=0.0717, 95% CI, -0.1809 to 0.3243, *P*=0.558)


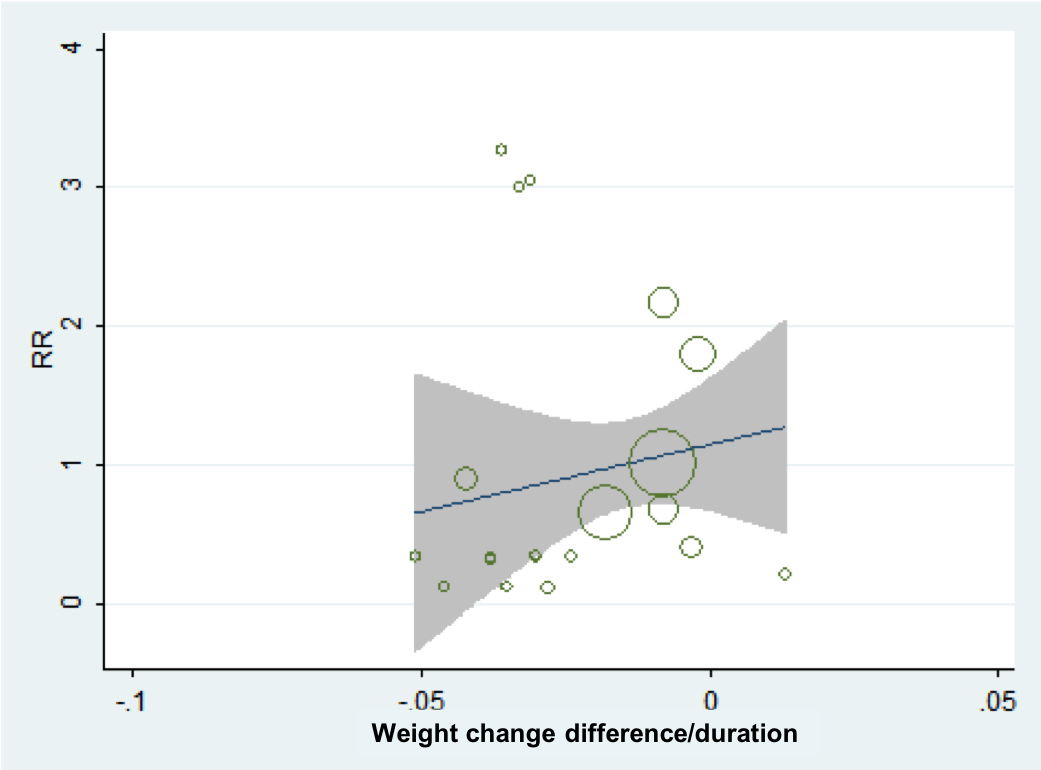


1. Weight reduction rate and incidence of hematologic neoplasm (β=9.6673, 95% CI, -19.7928 to 39.1275, *P*=0.499)

**Figure S22. The association between weight reduction difference or change rate and the incidence of thyroid neoplasm**


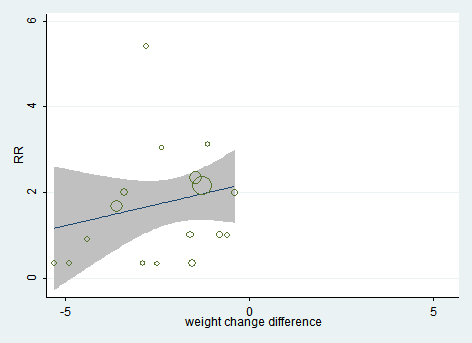


1. Weight reduction difference and incidence of thyroid neoplasm (β=0.1992, 95% CI, -0.2352 to 0.6336, *P*=0.344)


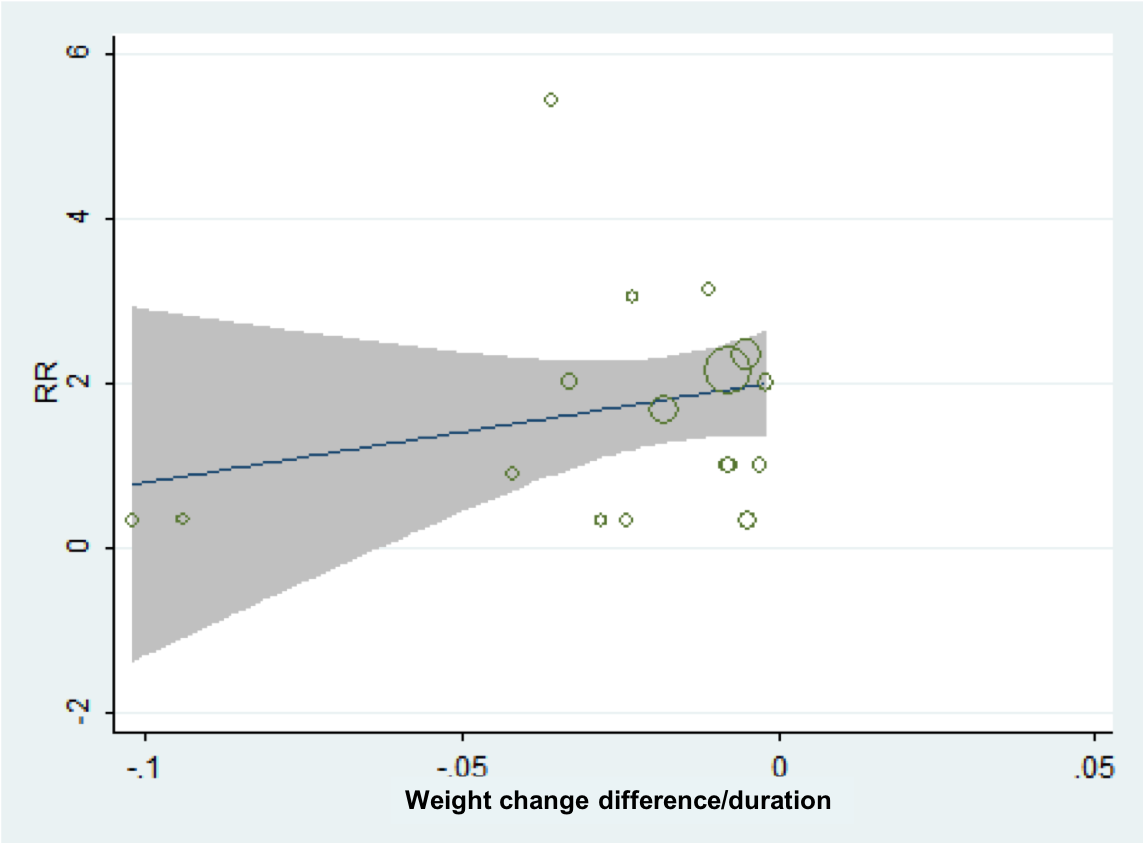


1. Weight reduction rate and incidence of thyroid neoplasm (β=12.1995, 95% CI, -13.6244 to 38.0234, *P*=0.330)

**Figure S23. The association between weight reduction difference or change rate and the incidence of ovarian neoplasm**


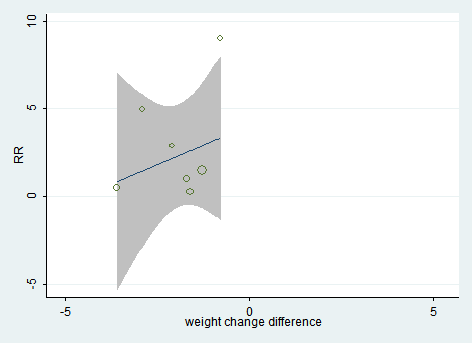


1. Weight reduction difference and incidence of ovarian neoplasm (β=1.1428, 95% CI, -2.3191 to 4.6047, *P*=0.435)


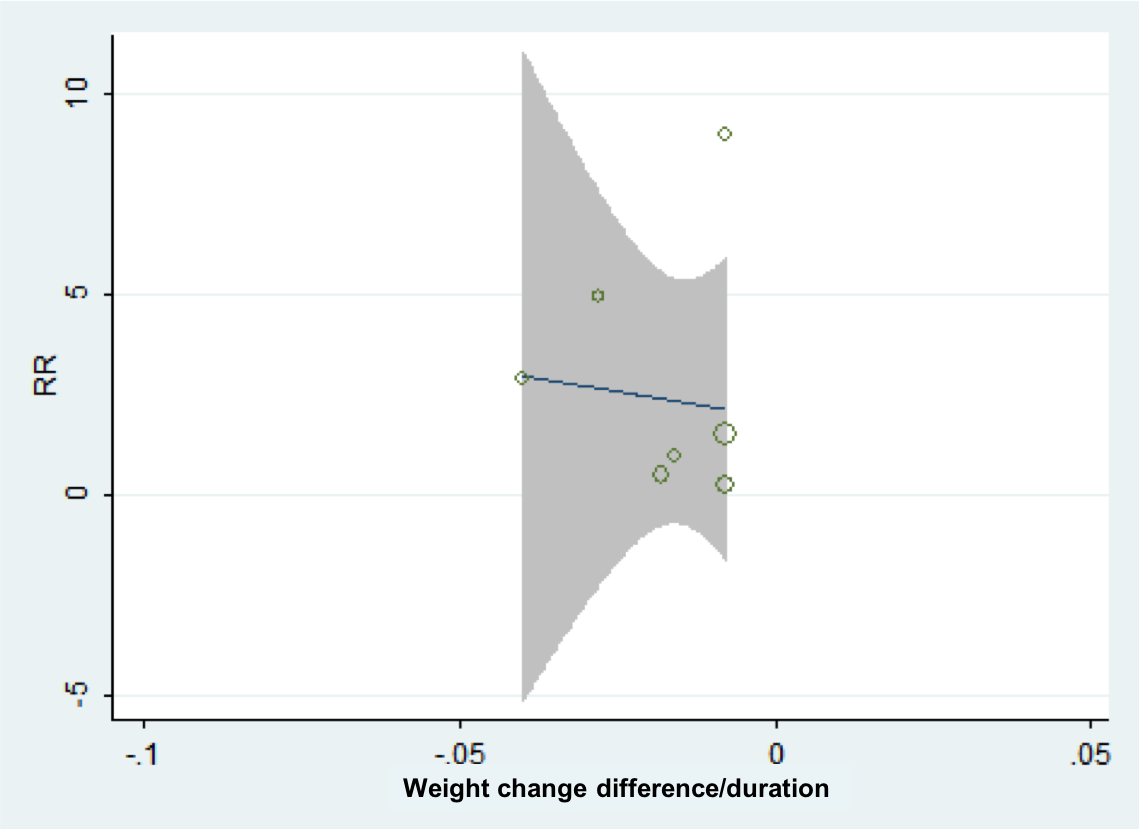


1. Weight reduction rate and incidence of ovarian neoplasm (β=-7.831, 95% CI, -308.4454 to 292.7841, *P*=0.949)

**Figure S24. The association between weight reduction difference or change rate and the incidence of renal neoplasm**


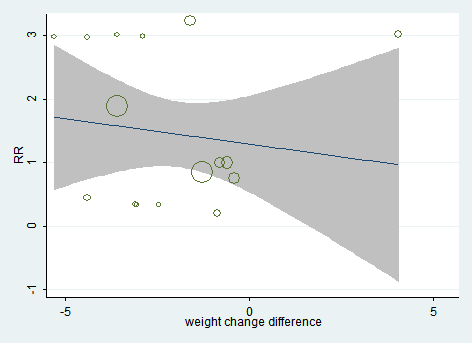


1. Weight reduction difference and incidence of renal neoplasm (β=-0.0282, 95% CI, -0.3426 to 0.2862, *P*=0.850)


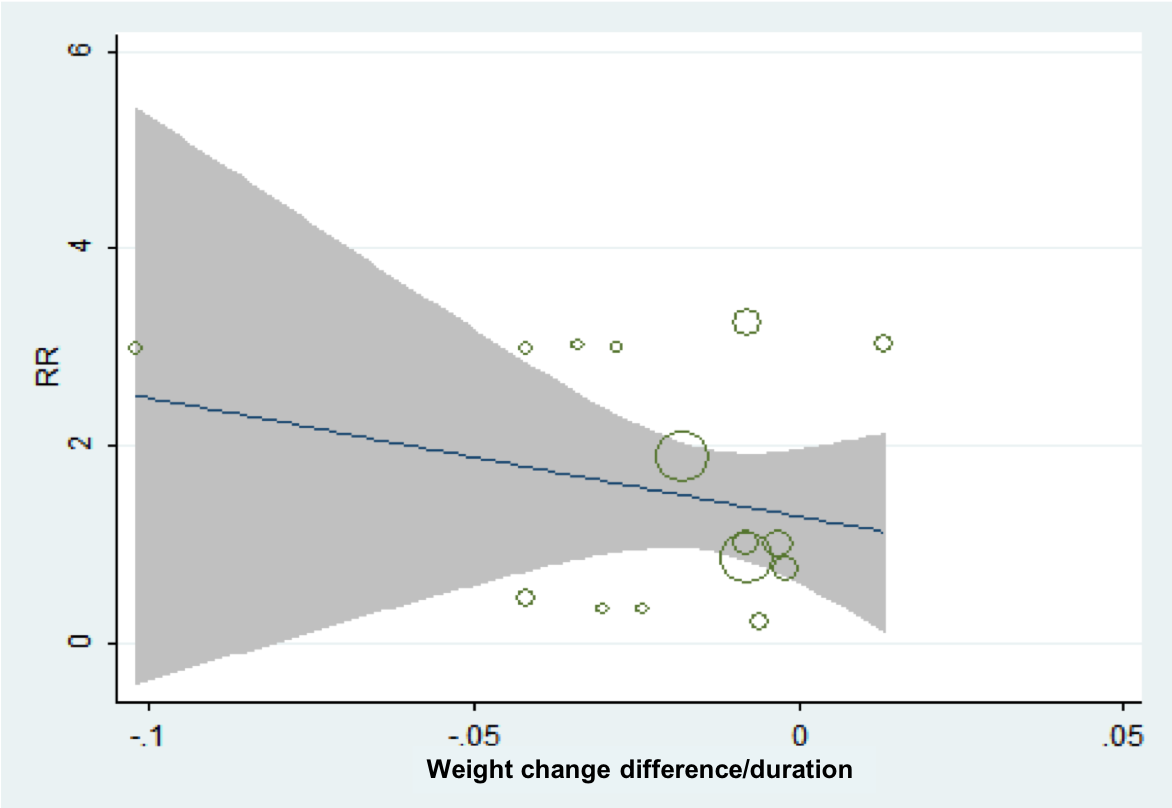


1. Weight reduction rate and incidence of renal neoplasm (β=-9.5417, 95% CI, -40.5564 to 21.4731, *P*=0.520)

**Figure S25. The association between weight reduction difference or change rate and the incidence of hepatic neoplasm**


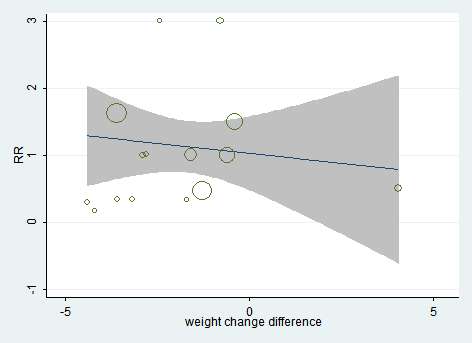


1. Weight reduction difference and incidence of hepatic neoplasm (β=-0.0551, 95% CI, -0.3393 to 0.2292, *P*=0.682)


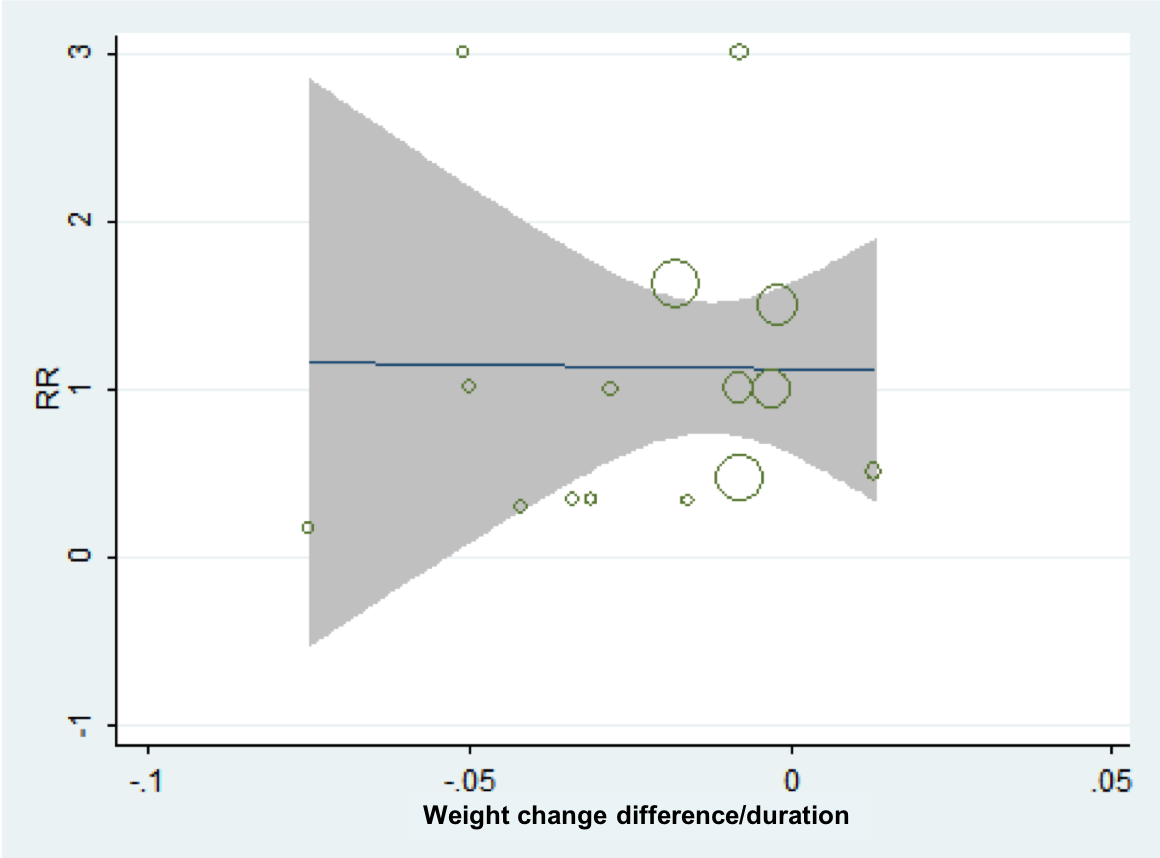


1. Weight reduction rate and incidence of hepatic neoplasm (β=-0.0914, 95% CI, -31.3743 to 31.1916, *P*=0.995)

**Figure S26. The association between weight reduction difference or change rate and the incidence of gallbladder/bile duct neoplasm**


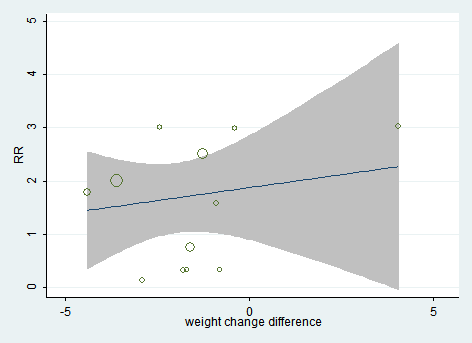


1. Weight reduction difference and incidence of gallbladder/bile duct neoplasm (β=-0.0971, 95% CI, -0.3391 to 0.5333, *P*=0.630)


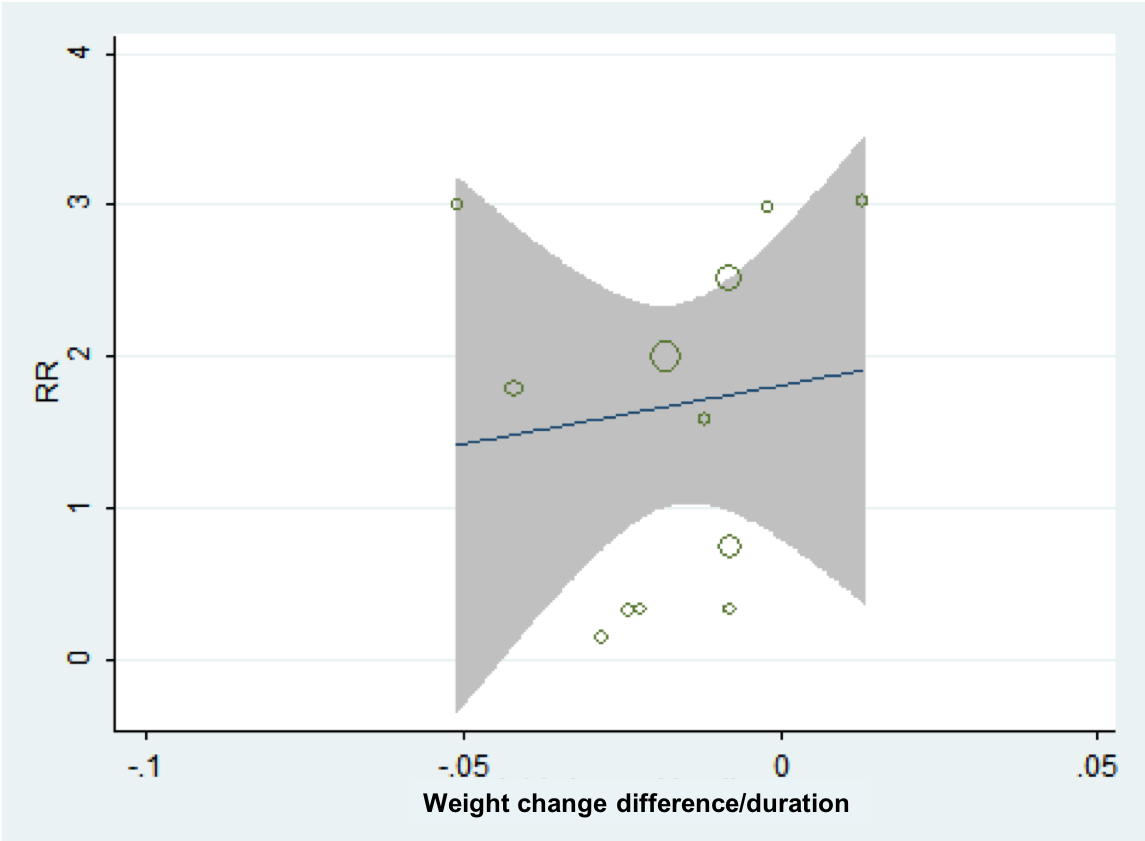


1. Weight reduction rate and incidence of gallbladder/bile duct neoplasm (β=7.7338, 95% CI, -47.8411 to 63.3086, *P*=0.763)

**Figure S27. The association between weight reduction difference or change rate and the incidence of head and neck neoplasm**


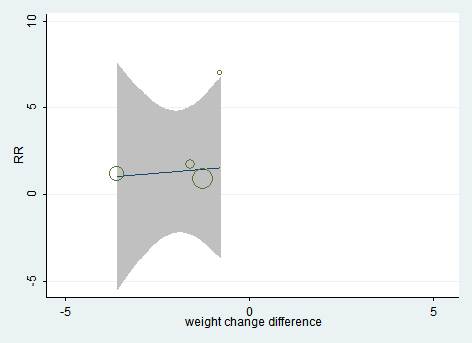


1. Weight reduction difference and incidence of head and neck neoplasm (β=1.1362, 95% CI, -4.5467 to 6.8190, *P*=0.480)


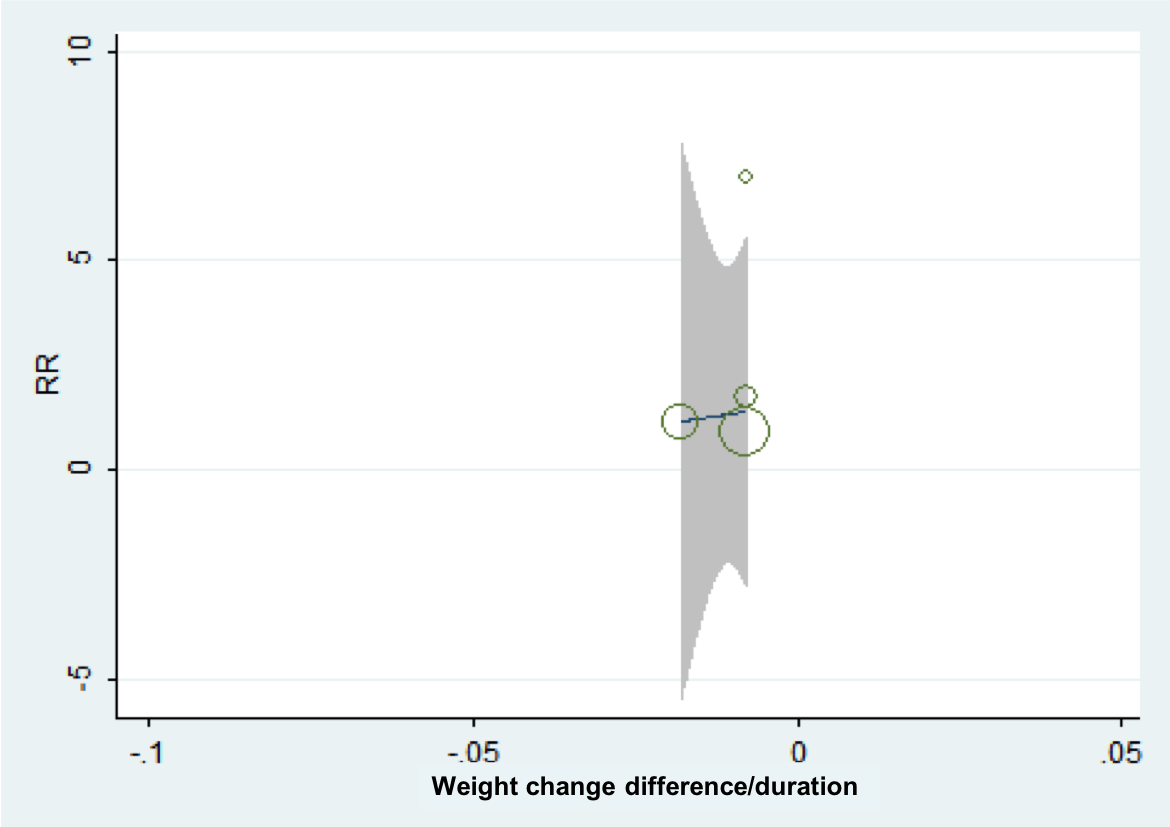


1. Weight reduction rate and incidence of head and neck neoplasm (β=181.5922, 95% CI, -1361.587 to 1724.771, p=0.663)

**REFERENCE**

1. UK Prospective Diabetes Study (UKPDS) Group. Intensive blood-glucose control with sulphonylureas or insulin compared with conventional treatment and risk of complications in patients with type 2 diabetes (UKPDS 33). Lancet 1998; 352: 837–53.
2. Duckworth W, Abraira C, Moritz T, et al, and the VADT Investigators. Glucose control and vascular complications in veterans with type 2 diabetes. N Engl J Med 2009; 360: 129–39.
3. Home PD, Pocock SJ, Beck-Nielsen H, et al, and the RECORD Study Team. Rosiglitazone evaluated for cardiovascular outcomes in oral agent combination therapy for type 2 diabetes (RECORD): a multicentre, randomised, open-label trial. Lancet 2009; 373: 2125–35.
4. Hollander P, Cooper J, Bregnhøj J, Pedersen CB. A 52-week, multinational, open-label, parallel-group, noninferiority, treat-to-target trial comparing insulin detemir with insulin glargine in a basal-bolus regimen with mealtime insulin aspart in patients with type 2 diabetes. Clinical Therapeutics 2008; 30: 1976-87.
5. Rosenstock J, Davies M, Home PD, et al. A randomised, 52-week, treat-to-target trial comparing insulin detemir with insulin glargine when administered as add-on to glucose-lowering drugs in insulin-naive people with type 2 diabetes. Diabetologia 2008; 51: 408-16.
6. Rosenstock J, Fonseca V, McGill JB, et al. Similar progression of diabetic retinopathy with insulin glargine and neutral protamine Hagedorn (NPH) insulin in patients with type 2 diabetes: a long-term, randomised, open-label study. Diabetologia 2009; 52: 1778-88.
7. Aroda VR, González-Galvez G, Grøn R, et al. Durability of insulin degludec plus liraglutide versus insulin glargine U100 as initial injectable therapy in type 2 diabetes (DUAL VIII): a multicentre, open-label, phase 3b, randomised controlled trial. Lancet Diabetes Endocrinol 2019;7:596-605.
8. Ahren B, Johnson SL, Stewart M, et al. HARMONY 3: 104-week randomized, double-blind, placebo- and active-controlled trial assessing the efficacy and safety of albiglutide compared with placebo, sitagliptin, and glimepiride in patients with type 2 diabetes taking metformin. Diabetes care 2014;37:2141-8.
9. Weinstock RS, Guerci B, Umpierrez G, Nauck MA, Skrivanek Z, Milicevic Z. Safety and efficacy of once-weekly dulaglutide versus sitagliptin after 2 years in metformin-treated patients with type 2 diabetes (AWARD-5): a randomized, phase III study. Diabetes ObesMetab 2015;17:849-58.
10. Davies MJ, Bergenstal R, Bode B, et al. Efficacy of Liraglutide for Weight Loss Among Patients with Type 2 Diabetes: The SCALE Diabetes Randomized Clinical Trial. JAMA 2015;314:687-99.
11. Home PD, Shamanna P, Stewart M, et al. Efficacy and tolerability of albiglutide versus placebo or pioglitazone over 1 year in people with type 2 diabetes currently taking metformin and glimepiride: HARMONY 5. Diabetes ObesMetab 2015;17:179-87.
12. Wilding JP, Woo V, Soler NG, et al. Long-term efficacy of dapagliflozin in patients with type 2 diabetes mellitus receiving high doses of insulin: a randomized trial. Annals of internal medicine 2012;156:405-15.
13. Rosenstock J, Vico M, Wei L, SalsaliA,etal. Effects of dapagliflozin, an SGLT2 inhibitor, on HbA(1c), body weight, and hypoglycemia risk in patients with type 2 diabetes inadequately controlled on pioglitazone monotherapy. Diabetes care 2012;35:1473-8.
14. SchernthanerG , Gross J L , Rosenstock J , et al. Canagliflozin Compared With Sitagliptin for Patients With Type 2 Diabetes Who Do Not Have Adequate Glycemic Control With Metformin Plus Sulfonylurea. Diabetes Care 2013; 36:2508-2515.
15. Wilding JPH, Charpentier G, Hollander P, et al. Efficacy and safety of canagliflozin in patients with type 2 diabetes mellitus inadequately controlled with metformin and sulphonylurea: a randomised trial. International Journal of Clinical Practice 2013;67:1267-82.
16. Lavalle-Gonzalez FJ, Januszewicz A, Davidson J, et al. Efficacy and safety of canagliflozin compared with placebo and sitagliptin in patients with type 2 diabetes on background metformin monotherapy: a randomised trial. Diabetologia2013;56:2582-92.
17. Bailey CJ, Gross JL, Hennicken D, etal. Dapagliflozin add-on to metformin in type 2 diabetes inadequately controlled with metformin: a randomized, double-blind, placebo-controlled 102-week trial. BMC Medicine 2013;11:43
18. Ferrannini E, Berk A, Hantel S, et al. Long-Term Safety and Efficacy of Empagliflozin, Sitagliptin, and Metformin: An active-controlled, parallel-group, randomized, 78-week open-label extension study in patients with type 2 diabetes. Diabetes Care 2013; 36:4015-4021.
19. Barnett AH, Mithal A, Manassie J, et al. Efficacy and safety of empagliflozin added to existing antidiabetes treatment in patients with type 2 diabetes and chronic kidney disease: a randomised, double-blind, placebo-controlled trial. Lancet Diabetes Endocrinol 2014;2:369-84.
20. Rosenstock J, Jelaska A, Frappin G, et al. Improved glucose control with weight loss, lower insulin doses, and no increased hypoglycemia with empagliflozin added to titrated multiple daily injections of insulin in obese inadequately controlled type 2 diabetes. Diabetes care 2014;37:1815-23.
21. Yale JF, Bakris G, Cariou B, et al. Efficacy and safety of canagliflozin over 52 weeks in patients with type 2 diabetes mellitus and chronic kidney disease. Diabetes ObesMetab2014;16:1016-27.
22. Bolinder J ,Ljunggren, Johansson L , et al. Dapagliflozin maintains glycaemic control while reducing weight and body fat mass over 2 years in patients with type 2 diabetes mellitus inadequately controlled on metformin. Diabetes ObesMetab 2014, 16:159-169.
23. Nauck M A , Del Prato S , Durán-García, S, et al. Durability of glycaemic efficacy over 2?years with dapagliflozin versus glipizide as add-on therapies in patients whose type 2 diabetes mellitus is inadequately controlled with metformin. Diabetes ObesMetab 2014;16:1111-1120.
24. Kohan DE, Fioretto P, Tang W, et al. Long-term study of patients with type 2 diabetes and moderate renal impairment shows that dapagliflozin reduces weight and blood pressure but does not improve glycemic control. Kidney Int;2014;85:962-71.
25. Leiter LA, Cefalu WT, de Bruin TW, etal. Dapagliflozin added to usual care in individuals with type 2 diabetes mellitus with preexisting cardiovascular disease: a 24-week, multicenter, randomized, double-blind, placebo-controlled study with a 28-week extension. J Am GeriatrSoc;2014;62:1252-62.
26. Bode B, Stenlof K, Harris S, et al. Long-term efficacy and safety of canagliflozin over 104 weeks in patients aged 55-80 years with type 2 diabetes. Diabetes ObesMetab;2015;17:294-303.
27. Cefalu WT, Leiter LA, de Bruin TW, Gause-Nilsson I, Sugg J, Parikh SJ. Dapagliflozin's Effects on Glycemia and Cardiovascular Risk Factors in High-Risk Patients with Type 2 Diabetes: A 24-Week, Multicenter, Randomized, Double-Blind, Placebo-Controlled Study With a 28-Week Extension. Diabetes care. 2015;38(7):1218-27.
28. Haering HU, Merker L, Christiansen AV, et al. Empagliflozin as add-on to metformin plus sulphonylurea in patients with type 2 diabetes. Diabetes Res ClinPract. 2015; 110(1):82-90.
29. Lewin A ,Defronzo R A , Patel S , et al. Erratum. Initial Combination of Empagliflozin and Linagliptin in Subjects with Type 2 Diabetes. Diabetes Care 2015;38:394–402.
30. Rosenstock J, Jelaska A, Zeller C, et al. Impact of empagliflozin added on to basal insulin in type 2 diabetes inadequately controlled on basal insulin: a 78-week randomized, double-blind, placebo-controlled trial. Diabetes ObesMetab2015;17:936-48.
31. Leiter LA, Yoon KH, Arias P, et al. Canagliflozin provides durable glycemic improvements and body weight reduction over 104 weeks versus glimepiride in patients with type 2 diabetes on metformin: a randomized, double-blind, phase 3 study. Diabetes Care 2015;38(3):355-64.
32. Bailey CJ, Morales Villegas EC, Woo V, Tang W, Ptaszynska A, List JF. Efficacy and safety of dapagliflozin monotherapy in people with Type 2 diabetes: a randomized double-blind placebo-controlled 102-week trial. Diabet Med 2015;32:531-41.
33. Dagogo-Jack S, Liu J, Eldor R, et al. Efficacy and safety of the addition of ertugliflozin in patients with type 2 diabetes mellitus inadequately controlled with metformin and sitagliptin: The VERTIS SITA2 placebo-controlled randomized study. Diabetes, obesMetab 2018; 20(3):530-540.
34. Müller‐Wieland D, Kellerer M, Cypryk K, et al. Efficacy and safety of dapagliflozin or dapagliflozin plus saxagliptin versus glimepiride as add‐on to metformin in patients with type 2 diabetes. Diabetes, obesMetab 2018;20:2598-607.
35. Gallo S, Charbonnel B, Goldman AA-Ohoo, et al. Long-term efficacy and safety of ertugliflozin in patients with type 2 diabetes mellitus inadequately controlled with metformin monotherapy: 104-week VERTIS MET trial. Diabetes ObesMetab 2019;7[Epub ahead of print].
36. Pfeffer MA, Claggett B, Diaz R, et al. Lixisenatide in Patients with Type 2 Diabetes and Acute Coronary Syndrome. N Engl J Med 2015; 373: 2247-2257.
37. Zinman B, Wanner C, Lachin JM, et al. Empagliflozin, Cardiovascular Outcomes, and Mortality in Type 2 Diabetes. N Engl J Med 2015; 373: 2117-2128.
38. Marso SP, Daniels GH, Brown-Frandsen K, et al. Liraglutide and Cardiovascular Outcomes in Type 2 Diabetes. N Engl J Med 2016; 375: 311-322.
39. [Marso SP](https://www.ncbi.nlm.nih.gov/pubmed/?term=Marso%20SP%5BAuthor%5D&cauthor=true&cauthor_uid=27633186), [Bain SC](https://www.ncbi.nlm.nih.gov/pubmed/?term=Bain%20SC%5BAuthor%5D&cauthor=true&cauthor_uid=27633186), [Consoli A](https://www.ncbi.nlm.nih.gov/pubmed/?term=Consoli%20A%5BAuthor%5D&cauthor=true&cauthor_uid=27633186), et al. Semaglutide and Cardiovascular Outcomes in Patients with Type 2 Diabetes. N Engl J Med 2016; 375: 1834-1844.
40. Holman R R, Bethel M A, Mentz R J , et al. Effect of once-weekly exenatide on cardiovascular outcomes in type 2 diabetes. N Engl J Med 2017, 377(13):1228.
41. [Neal B](https://www.ncbi.nlm.nih.gov/pubmed/?term=Neal%20B%5BAuthor%5D&cauthor=true&cauthor_uid=28605608), [Perkovic V](https://www.ncbi.nlm.nih.gov/pubmed/?term=Perkovic%20V%5BAuthor%5D&cauthor=true&cauthor_uid=28605608), [Mahaffey KW](https://www.ncbi.nlm.nih.gov/pubmed/?term=Mahaffey%20KW%5BAuthor%5D&cauthor=true&cauthor_uid=28605608), et al. Canagliflozin and Cardiovascular and Renal Events in Type 2 Diabetes. N Engl J Med 2017; 377(7): 644-657.
42. Hernandez AF, Green JB, Janmohamed S, et al. Albiglutide and cardiovascular outcomes in patients with type 2 diabetes and cardiovascular disease (Harmony Outcomes): a double-blind, randomised placebo-controlled trial. Lancet (London, England) 2018; 392(10157):1519-29.
43. Wiviott SD, Raz I, Bonaca MP, et al. Dapagliflozin and Cardiovascular Outcomes in Type 2 Diabetes. N Engl J Med 2018;380(4):347-57.
44. Perkovic V, Jardine MJ, Neal B, et al. Canagliflozin and Renal Outcomes in Type 2 Diabetes and Nephropathy. N Engl J Med 2019;380:2295-306.
45. Gerstein HC, Colhoun HM, Dagenais GR, et al. Dulaglutide and cardiovascular outcomes in type 2 diabetes (REWIND): a double-blind, randomised placebo-controlled trial.Lancet 2019;394:121-130.
46. Rosenstock J, Kahn SE, Johansen OE, et al. Effect of Linagliptin vs Glimepiride on Major Adverse Cardiovascular Outcomes in Patients With Type 2 Diabetes: The CAROLINA Randomized Clinical Trial. JAMA 2019;322(12):1155–1166.
